# HCVerso3: An Open-Label, Phase IIb Study of Faldaprevir and Deleobuvir with Ribavirin in Hepatitis C Virus Genotype-1b-Infected Patients with Cirrhosis and Moderate Hepatic Impairment

**DOI:** 10.1371/journal.pone.0168544

**Published:** 2016-12-28

**Authors:** Christoph Sarrazin, Michael Manns, Jose Luis Calleja, Javier Garcia-Samaniego, Xavier Forns, Renee Kaste, Xiaofei Bai, Jing Wu, Jerry O. Stern

**Affiliations:** 1 J.W. Goethe-University Hospital, Frankfurt am Main, Germany; 2 Hannover Medical School, Hannover, Germany; 3 Hospital Universitario Puerta de Hierro, Universidad Autonomy de Madrid. CIBERehd, Madrid, Spain; 4 Hospital Universitario La Paz/Carlos III, CIBERehd, IdiPAZ, Madrid, Spain; 5 Hospital Clínic, CIBERehd, IDIBAPS, Barcelona, Spain; 6 Boehringer Ingelheim Pharmaceuticals, Inc., Ridgefield, CT, United States of America; Taipei Veterans General Hospital, TAIWAN

## Abstract

This study evaluated the interferon-free, oral combination of deleobuvir (non-nucleoside HCV NS5-RNA-polymerase inhibitor) and faldaprevir (HCV NS3/4A-protease inhibitor) with ribavirin in patients with HCV genotype-1b and moderate (Child-Pugh B [CPB], n = 17) or mild hepatic impairment (Child-Pugh A [CPA], n = 18). Patients received faldaprevir 120 mg and deleobuvir (600 mg [CPA], 400 mg [CPB]) twice-daily with weight-based ribavirin for 24 weeks. Baseline characteristics were similar between groups. Among CPA patients, 13/18 completed treatment; discontinuations were for adverse events (AEs, n = 1), lack of efficacy (n = 3) and withdrawal (n = 1). Among CPB patients, 8/17 completed treatment; discontinuations were for AEs (n = 6), withdrawal (n = 1) and ‘other’ (n = 2). Sustained virologic response at post-treatment Week 12 (SVR12) was achieved by 11 (61%) CPA patients (95% confidence interval: 38.6%–83.6%) and 9 (53%) CPB patients (95% confidence interval: 29.2%–76.7%), including most CPA (11/16) patients with Week 4 HCV RNA <25 IU.mL^-1^ (target detected or not detected) and most CPB (8/9) patients with Week 4 HCV RNA <25 IU.mL^-1^ (target not detected); 0/4 CPB patients with Week 4 HCV RNA <25 IU.mL^-1^ (target detected) achieved SVR12. The most common AEs in both groups were nausea, diarrhoea and vomiting. Serious AEs were observed in 9 (53%) CPB patients and 1 (6%) CPA patient. Plasma trough concentrations of deleobuvir and faldaprevir were not substantially different between the CPA and CPB groups. In conclusion, in this small study the safety and efficacy profiles for 24 weeks of treatment with faldaprevir+deleobuvir+ribavirin in patients with mild or moderate hepatic impairment were consistent with the safety and efficacy profile of this regimen in non-cirrhotic patients. Faldaprevir+deleobuvir+ribavirin resulted in SVR12 in 53–61% of patients: proportions achieving SVR4 but not SVR12 were higher than in non-cirrhotic patients and overall response rates were lower than rates reported with other all-oral regimens in patients with cirrhosis.

***Trial Registration:*** ClinicalTrials.gov NCT01830127.

## Introduction

Chronic infection with hepatitis C virus (HCV) is a major cause of morbidity and mortality worldwide [1–3]. The most prevalent HCV genotype in North America and Europe, HCV genotype-1 (GT-1), has historically been difficult to treat with what was for many years the standard-of-care: peginterferon-α (PegIFN) and ribavirin [2, 4, 5]. In addition to poor efficacy against HCV GT-1, PegIFN is also poorly tolerated by many patients and has several contraindications such as neuropsychiatric disorders, low white blood cell count or platelet count and autoimmune disease [2]. Over the past decade, improvements in the understanding of the HCV lifecycle have led to the development of numerous oral direct-acting antiviral agents (DAAs) that act on several different viral targets, including NS3/4A serine protease, NS5B RNA polymerase and the multifunctional NS5A protein [2]. Initially treatment regimens using DAAs continued to rely on combination with PegIFN [6, 7]. More recently, recognising the limitations of PegIFN and the substantial improvements in simplicity, tolerability and efficacy of DAA treatment, attention is increasingly focusing on combining DAAs in IFN-free treatment strategies [8]. This field continues to rapidly evolve, with many novel agents and combination therapies now approved or in advanced stages of development [6, 7, 8].

Faldaprevir is an HCV NS3/4A protease inhibitor with a pharmacokinetic profile that supports once-daily dosing and potent *in vitro* activity against HCV genotype subtypes 1a and 1b [9, 10]. Deleobuvir is a non-nucleoside inhibitor of HCV NS5B RNA polymerase, which binds reversibly to the thumb-pocket I of NS5B resulting in potent and specific antiviral activity [11, 12]. *In vitro* and Phase 1 clinical study data show that deleobuvir is more active against GT-1b than against GT-1a [11, 12]. The IFN-free combination of once-daily faldaprevir, twice-daily deleobuvir and ribavirin, has been investigated in phase 2 and Phase 3 clinical studies in treatment-naïve patients with chronic HCV GT-1 infection [13–16]. Phase 3 studies of this combination assessed 16 or 24 weeks’ treatment in HCV genotype-1b-infected, treatment-naïve patients, including patients with compensated cirrhosis. After 24 weeks’ treatment, adjusted rates of sustained virologic response at 12 weeks post treatment (SVR12) were 81% among patients without cirrhosis and 72–74% among patients with cirrhosis (significantly higher than historical controls in both cases) [16]. Treatment of patients without cirrhosis for 16 weeks resulted in high relapse rates and lower SVR rates (72%-76%) [16].

In patients with chronic HCV infection and decompensated cirrhosis, anti-HCV treatment is important since achieving an SVR reduces the risk of clinical decompensation and hepatocellular carcinoma and can prevent otherwise certain reinfection following liver transplant [2, 17]. The objectives of the current study were to evaluate the pharmacokinetic profile and safety of deleobuvir in combination with once-daily faldaprevir and weight-based ribavirin in a small group of patients with moderate hepatic impairment (Child-Pugh B [CPB]) compared with patients with mild hepatic impairment (Child-Pugh A [CPA]). Based on the results of phase 3 trials in treatment-naïve patients [16], the development of faldaprevir in combination with deleobuvir has been halted. However, faldaprevir has been licensed to another company and continues development, with phase 2 clinical studies of faldaprevir in combination with other novel DAAs initiated (ClinicalTrials.gov NCT02716428 and NCT02593162).

## Materials and Methods

### Study design and patient population

This was an open-label, phase 2b study recruiting both treatment-naïve and treatment-experienced HCV genotype-1b-infected patients at multiple clinical centres in Europe and North America (ClinicalTrials.gov NCT01830127). The key inclusion criteria were: age 18–75 years; HCV RNA ≥1000 IU.mL^-1^ at screening; liver cirrhosis with Metavir Grade 4 or Ishak ≥5 on biopsy, or liver stiffness of ≥13 kPa on fibroscan; patients who were pegylated IFN/ribavirin treatment-experienced must have experienced relapse, partial response, or intolerance before week 12 of treatment. The key exclusion criteria were: HIV or HBV co-infection; confirmed or suspected malignancy (current or within 5 years); total bilirubin >3 mg/dL, serum albumin <2.4 g.dL^-1^ and international normalized ratio >2.3; Child-Pugh C.

The protocol was approved by the Institutional Review Board or Independent Ethics Committee of all participating sites (Ethikkommission der Medizinischen Hochschule Hanover; Hospital Universitario Puerta de Hierro de Majadahonda, CEIC; CEIC Hospital Clínic de Barcelona, Agencia de Ensayos Clínicos, Servicio de Farmacia; Hospital Carlos III Comité Ético de Investigación Clínica, CEIC Hospital Universitario la Paz, Paseo de la Castellana; CEIC Hospital Universitari Vall d´Hebrón, Unidad de Soporte al CEIC (SCEI), Vall d´Hebrón Institut de Recerca (VHIR); Consorcio Hospital General, Universitario de Valencia Comité, Ético de Investigación Clínica; NRES Committee; Chesapeake IRB). All patients provided written informed consent. The trial took place between 24 April 2013 and 21 October 2014 and was carried out in accordance with the principles of the Declaration of Helsinki.

### Treatment

All patients received faldaprevir 120 mg (once-daily, with an additional 120 mg loading dose on Day 1) and weight-based ribavirin (twice-daily). In addition, patients were assigned to two different doses of deleobuvir based on their hepatic impairment status: CPA patients were treated with deleobuvir 600 mg twice-daily and CPB patients were treated with deleobuvir 400 mg twice-daily. All treatments were taken orally and were self-administered by the patients. Faldaprevir was taken in the morning together with deleobuvir and ribavirin, and together with food. The evening doses of deleobuvir and ribavirin were taken together with food 12 hours after the morning dose. All patients were treated for 24 weeks and followed up for 12 weeks after the end of treatment.

### Assessments

The primary efficacy endpoint was SVR12, defined as plasma HCV RNA level <25 IU.mL^-1^ 12 weeks after end of treatment. SVR4 was a secondary endpoint. Plasma HCV RNA level was measured using the quantitative Roche COBAS® Taqman HCV/HPS assay (Version 2), with a limit of detection between 10 and 20 IU/mL and a linear range of 25 IU/mL to 3.91 x 10^8^ IU/mL. Safety was assessed by monitoring adverse events (AEs, reported using the MedDRA coding dictionary version 17.1 and the NIH NIAID Division of AIDS [DAIDS] grading system), AEs leading to treatment discontinuation, serious AEs (SAEs), laboratory test abnormalities, and changes in laboratory test values. Pre-dose plasma trough concentrations for deleobuvir and faldaprevir were measured at Week 1 through Week 4. A validated high-performance liquid chromatography–tandem mass spectrometry (HPLC-MS/MS) assay was used to analyze the plasma samples (Tandem Labs, Salt Lake City, UT, USA). The faldaprevir and deleobuvir methods were validated for a range of 10.0 to 10,000 ng/ml and 23.0 to 23,000 nmol/L respectively; analyte quantitation in both methods was performed using a weighted (1/x^2^) linear least squares regression analysis generated from calibration standards.

### Statistical analysis

All efficacy, safety and pharmacokinetic endpoints were analyzed in a descriptive manner using SAS® Version 9.2. The primary efficacy analysis was carried out on an intent-to-treat basis. Secondary efficacy and safety analyses were carried out for patients who received at least one dose of study treatment. Patients who discontinued from the trial without reaching the SVR time points were counted as treatment failures. Patients with missing SVR4 were imputed by SVR12, if available. Missing SVR12 data were treated as failure.

Pharmacokinetic results are provided as geometric means. The coefficient of variation of the geometric mean was calculated using Phoenix WinNonLin 6.3 using the equation:

$$\sqrt{\exp\left({\left(SD\_log\right)}^{2}\right)-1}\times 100$$

The objective of the pharmacokinetic analysis was to determine if the doses of faldaprevir and deleobuvir administered to CPB patients provided trough levels equal to those seen in CPA patients. For this analysis a sample size of 15 patients per arm was sufficient to provide 67% power (with a coefficient of variation of 100%) to detect differences when alpha = 0.1 and the lower equivalence bound of the trough ratio was set at 0.5 and upper bound set at 2.

## Results

**Fig 1:**
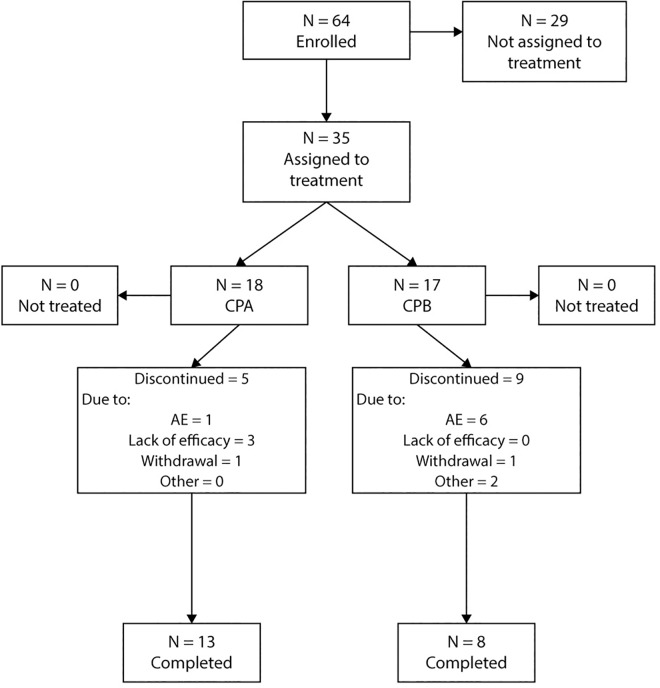
Patient disposition. AE, adverse event; CPA, Child-Pugh A group; CPB, Child-Pugh B group.

Of the 64 patients who were enrolled, 35 were assigned to treatment: 18 in the CPA group and 17 in the CPB group. Of the 29 enrolled patients who were never treated, 24 were screening failures and 5 withdrew from the study for unspecified reasons. Of the 18 treated patients in the CPA group, 13 completed treatment. Reasons for discontinuation were: 1 AE, 3 lack of efficacy and 1 withdrawal. Of the 17 treated patients in the CPB group, 8 completed treatment (Fig 1). Reasons for discontinuation were: 6 AEs, 1 withdrawal and 2 ‘other’. Efficacy and safety analyses were carried out for all 35 patients who were assigned to treatment. A total of 6 patients had at least one important protocol violation: 3 patients did not receive the additional faldaprevir loading dose on Day 1; a further 3 patients were assigned to the wrong dose of deleobuvir (based on their hepatic impairment status) and were excluded from the pharmacokinetic analysis population.

Most patients were male (20/35), all were white, the mean age was 57.2 (standard deviation [SD] 9.1) years and the mean BMI was 28.8 (SD 5.6) kg.m^-2^. Baseline characteristics of these patients were similar between groups (Table 1).

**Table 1:** Patient baseline characteristics.

	CPA (N = 18)	CPB (N = 17)
Male, n (%)	10 (56)	10 (59)
Mean age, years (SD)	57.8 (8.8)	56.6 (9.7)
Race: white, n (%)	18 (100)	17 (100)
Mean BMI, kg.m^-2^ (SD)	28.6 (6.1)	29 (5.1)
*IL-28B* genotype (rs12979860), n (%) • CC • CT • TT • Missing	• 2 (11) • 10 (56) • 1 (5.6) • 5 (28)	• 6 (35) • 7 (41) • 3 (18) • 1 (6)
Mean HCV RNA, log_10_(IU).mL^-1^, (SD)	6.5 (0.6)	6.2 (0.4)
HCV RNA ≥800,000 IU.mL^-1^, n (%)	14 (78)	12 (71)

CPA, Child-Pugh A; CPB, Child-Pugh B; SD, standard deviation.

Mean treatment exposures were 141 (SD 49) days for CPA patients and 114 (SD 65) days for CPB patients. The majority of CPA patients (12/18) had ≥24 weeks of exposure compared with 5/17 CPB patients; the majority of CPB patients (10/17) had ≥16 weeks of exposure.

SVR12 was achieved by comparable proportions of CPA and CPB patients (Table 2). The majority of CPA (11/16) and CPB (8/13) patients with Week 4 HCV RNA levels <25 IU.mL^-1^ achieved SVR12. Of 9 and 7 CPA patients who had a Week 4 HCV RNA levels <25 IU.mL^-1^ (undetected and detected, respectively), the majority (66.7% [n = 6] and 71.4% [n = 5], respectively) achieved SVR12. Of the 9 CPB patients who had Week 4 HCV RNA <25 IU.mL^-1^ (undetected), 88.9% (n = 8) achieved SVR12. None of the 4 CPB patients with Week 4 HCV RNA levels <25 IU.mL^-1^ (detected) achieved SVR12.

**Table 2:** Efficacy results.

Efficacy parameter	CPA (N = 18)		CPB (N = 17)	
	n/N (%)	95% CI	n/N (%)	95% CI
Patients achieving SVR				
SVR12	11/18 (61)	38.6–83.6	9/17 (53)	29.2–76.7
SVR4	13/18 (72)	51.5–92.9	13/17 (77)	56.3–96.6
SVR12 by Week 4 HCV RNA				
<25 IU.mL^-1^, target not detected	6/9 (67)		8/9 (89)	
<25 IU.mL-1, target detected	5/7 (71)		0/4 (0)	
≥25 IU.mL-1	0/1 (0)		1/1 (100)	
Missing	0/1 (0)		0/3 (0)	

CPA, Child-Pugh A; CPB, Child-Pugh B; SD, standard deviation; SVR, sustained virologic response.

AE data are summarized in Table 3. The most common AEs in both groups were gastrointestinal: nausea, diarrhoea and vomiting. The majority of gastrointestinal events were mild or moderate with only two DAIDS grade 3 events (upper abdominal pain, leading to treatment discontinuation, in one CPB patient; diarrhoea and vomiting in a second patient). AE data are summarized in Table 3.

**Table 3:** Summary of treatment-emergent AEs.

Patients with an event, n (%)		CPA (N = 18)	CPB (N = 17)
Any AE		17 (94)	17 (100)
DAIDS Grade ≥3 AEs		5 (28)	9 (53)
Drug-related AEs^a^		16 (89)	16 (94)
AEs leading to discontinuation	of ribavirin	0 (0)	2 (12)
	of all study treatments	0^b^ (0)	7 (41)^c^
Serious AEs		1 (6)	9 (53)
Common AEs^d^	Nausea	13 (72)	13 (76.5)
	Diarrhoea	9 (50)	10 (59)
	Vomiting	9 (50)	7 (41)
	Ascites	0	8 (47)
	Abdominal distension	5 (28)	1 (6)
	Dizziness	1 (6)	5 (29)
	Hepatic encephalopathy	0	5 (29)
	Asthenia	7 (39)	7 (41)
	Oedemia peripheral	4 (22)	5 (29)
	Fatigue	4 (22)	3 (18)
	Pruritus	8 (44)	5 (29)
	Jaundice	5 (28)	8 (47)
	Hyperbilirubinaemia	8 (44)	4 (24)
	Anaemia	6 (33)	9 (53)
	Insomnia	3 (17)	4 (24)
	Ocular icterus	6 (33)	3 (18)
	Decreased appetite	5 (28)	2 (12)
	Urinary tract infection	0	4 (24)

AE, adverse event; CPA, Child-Pugh A; CPB, Child-Pugh B; DAIDS, NIH NIAID division of AIDS.

^a^Investigator-assigned.

^b^One CPA patient reported an adverse event of nausea that started 32 days before treatment and led to treatment discontinuation.

^c^One CPB patient who was included in the database as having discontinued due to “other”, actually discontinued because of an adverse event.

^d^Occurring in at least 20% of patients in either group.

Compared with CPA patients, a higher proportion of CPB patients reported serious AEs (SAE), AEs leading to discontinuation of study treatment and DAIDS Grade 3/4 AEs. There was one SAE in the CPA group, an upper gastrointestinal haemorrhage, which was considered related to study treatment. In the CPB group, there were 9 SAEs; those that occurred in more than 1 patient were: hepatic encephalopathy (4 patients, 2 related to treatment); and hepatic cirrhosis (3 patients, all related to treatment). Other SAEs considered to be related to treatment included diarrhoea, vomiting, and general physical health deterioration in one patient, and hyponatraemia in a second patient. No patients died during the treatment period or through the 28-day follow-up.

Plasma deleobuvir and faldaprevir trough concentrations are summarized in Table 4. Overall, after multiple oral doses, both deleobuvir and faldaprevir pre-dose plasma concentrations were not substantially different in CPA and CPB patients up to Week 4. Variability (gCV%) was generally high, particularly for the CPA group.

**Table 4:** Plasma deleobuvir and faldaprevir trough concentrations.

		CPA (N = 18; DBV 600 mg BID				CPB (N = 17; DBV 400 mg BID)			
		n	gMean (nmol/L)	gCV %	gMean/D^a^ (nmol/L/mg)	n	gMean (nmol/L)	gCV %	gMean/D^a^ (nmol/L/mg)
DBV	Wk 1	16	12,700	120	21.2	15	9230	64	23.1
	Wk 2	14	12,000	122	20.0	15	10,700	71	26.8
	Wk 3	15	7100	196	11.8	14	9460	76	23.7
	Wk 4	15	8390	109	14.0	11	12,500	36	31.3
			gMean (ng/mL)	gCV %			gMean (ng/mL)	gCV %	
FDV	Wk 1	16	8270	80		14	5660	56	
	Wk 2	14	8810	114		13	7840	64	
	Wk 3	15	6190	157		14	8850	61	
	Wk 4	14	5390	123		11	8590	43	

DBV, deleobuvir; FDV, faldaprevir; gCV, coefficient of variation of the geometric mean; gMean, geometric mean; Wk, week

^a^Dose normalized gMean trough concentrations.

## Discussion

In this small study in treatment-naïve and treatment-experienced patients with chronic HCV genotype-1b infection and mild or moderate hepatic impairment, the overall safety and efficacy profiles for 24 weeks of treatment with deleobuvir and faldaprevir in combination with ribavirin were generally consistent with the safety and efficacy profiles observed in non-cirrhotic patients [16]. Overall the majority of patients achieved SVR4 (74%) and SVR12 (57%); however, response rates were lower than rates achieved with other all-oral DAA regimen in HCV-infected patients with cirrhosis (>90% SVR12) [18, 19]. Notably, the trough concentrations of deleobuvir and faldaprevir over 4 weeks of treatment were not substantially different between the CPA and CPB groups and similar proportions of patients achieved SVR4 and SVR12. The proportion of patients who were SVR4 but not SVR12 responders was higher than reported in phase 3 studies in non-cirrhotic patients. In non-cirrhotic patients, 95% of patients achieving SVR4 went on to achieve SVR12 [16]; whereas, in the present study, only 77% (20/26) of patients with SVR4 also achieved SVR12 (Table 2). This is consistent with data from other all-oral combinations that require longer treatment durations (24 weeks rather than 12 weeks) to achieve SVR in patients with cirrhosis than in those without cirrhosis [2, 18, 19]. Response at treatment Week 4 appeared to predict SVR12 after 24 weeks of treatment, although the small number of patients precluded statistical analysis. This was particularly notable in patients with moderate hepatic impairment (CPB), where none of the 4 patients having Week 4 HCV RNA <25 IU.mL^-1^, but with target detected, achieved SVR12, whereas 8/9 (89%) of those with undetectable HCV RNA at Week 4 achieved SVR12. It is conceivable that the combination of faldaprevir and deleobuvir with ribavirin is not sufficient to prevent on-going viral replication in patients where residual virus is detected at Week 4. Of note, with more potent DAA combinations, detectable Week 4 HCV RNA is not predictive of treatment failure [2, 18, 19]. Consistent with a more severe disease state, discontinuations, AEs and SAEs were more common in patients with moderate hepatic impairment than in those with mild hepatic impairment. The higher rates of AEs in CPB patients is likely related to these patients having more severe liver disease and a more unstable condition. SAEs reported in CPB patients were primarily related to worsening of the underlying disease (including hepatic cirrhosis, acute hepatic failure, hepatic encephalopathy, ascites, haemorrhage and general physical health deterioration).

In conclusion, in this small study in treatment-naïve and treatment-experienced patients with chronic HCV genotype-1b infection and mild or moderate hepatic impairment, the response to 24 weeks of treatment with faldaprevir, deleobuvir and ribavirin was not durable, with 74% of patients achieving SVR4 but only 57% achieving SVR12. Since this study was initiated, the HCV field has changed rapidly with the advent of new DAAs and all-oral DAA combinations. Because of this and based on the results of the phase 3 HCVerso1 and HCVerso2 trials [16], the development of the faldaprevir–deleobuvir combination has been terminated.

## Supporting Information

S1 Appendix HCVerso3 Trial Protocol. (PDF)

S2 Appendix TREND checklist. (PDF)
