# Supplementary material for: HCVerso3: An Open-Label, Phase IIb Study of Faldaprevir and Deleobuvir with Ribavirin in Hepatitis C Virus Genotype-1b-Infected Patients with Cirrhosis and Moderate Hepatic Impairment
Source: PLoS One. 2016 Dec 28;11(12):e0168544. doi: 10.1371/journal.pone.0168544 (PMC5193411; doi:10.1371/journal.pone.0168544)
Supplement: S1 Appendix — (PDF) [file pone.0168544.s001.pdf]

Proprietary confidential information © 2015 Boehringer Ingelheim International GmbH or one or more of its affiliated companies

16. APPENDICES **1**
16.1 TRIAL INFORMATION **1**
16.1.1 Protocol and amendments **1**
16.1.1.1 Protocol **2**
16.1.1.2 Revised protocol and protocol amendments **83**

Proprietary confidential information © 2015 Boehringer Ingelheim International GmbH or one or more of its affiliated companies

16.1.1.1 Protocol 2
clinical-trial-protocol 3
ctp-signature-ci-manns-2012-12-13 82

Proprietary confidential information © 2015 Boehringer Ingelheim International GmbH or one or more of its affiliated companies

SIGNATURE INFORMATION

Document: 1241-0030--protocol

Document No.: U12-3886-01

Title A phase II randomised, double-blind and placebo-controlled study of BI 207127 in combination with faldaprevir and ribavirin in patients with moderate hepatic impairment (Child-Pugh B) with genotype 1b chronic hepatitis C infection

SIGNATURES (ELECTRONICALLY OBTAINED)

Meaning of Signature:	Signed by:	Date signed: (GMT)
Author-Trial Clinical Monitor	Kaste,Dr.,Renee	11/30/2012 22:19:07
Author-Trial Statistician	Guo,Dr.,Junhai	12/3/2012 19:07:21
Author-Trial Clinical Pharmacokineticist - On behalf of Sabo,John	Wu,Jing	12/5/2012 16:16:20
Approval-Team Member Medicine	Mensa,Dr.,Federico	12/5/2012 16:18:01
Approval-Therapeutic Area Head	Stern,Dr.,Jerry	12/10/2012 16:14:25

Proprietary confidential information © 2015 Boehringer Ingelheim International GmbH or one or more of its affiliated companies

Boehringer Ingelheim

SIGNATURE INFORMATION (continued)

Document 1241-0030--protocol

Document No.: U12-3886-01

Title A phase II randomised, double-blind and placebo-controlled study of BI 207127 in combination with faldaprevir and ribavirin in patients with moderate hepatic impairment (Child-Pugh B) with genotype 1b chronic hepatitis C infection

SIGNATURES (ELECTRONICALLY OBTAINED)
(There are no entries on this page if there are up to seven signatures.)

Meaning of Signature:	Signed by:	Date signed: (GMT)

Proprietary confidential information © 2015 Boehringer Ingelheim International GmbH or one or more of its affiliated companies

Boehringer Ingelheim

Clinical Trial Protocol

Doc. No.: U12-3886-01

EudraCT No.:	2012-003534-17
**BI Trial No.:**	1241.30
**BI Investigational Products:**	BI 207127 in combination with faldaprevir
**Title:**	A phase II randomised, double-blind and placebo-controlled study of BI 207127 in combination with faldaprevir and ribavirin in patients with moderate hepatic impairment (Child-Pugh B) with genotype 1b chronic hepatitis C infection
**Clinical Phase:**	Ib/IIb
**Trial Clinical Monitor:**	Renee Kaste, Ph.D.<br>Boehringer Ingelheim Pharmaceuticals, Inc.<br>900 Ridgebury Road<br>Ridgefield, Connecticut 06877<br>Phone: (203) 791-6626<br>Fax: (203) 798-5433
**Co-ordinating Investigator:**	Michael P. Manns, Ph.D.,M.D.<br>Zentrum Innere Medizin<br>Medizinische Hochschule Hannover<br>Carl-Neuberg-Straße 1<br>30625 Hannover<br>Phone: +49 (0) 5 11 - 5 32 33 05<br>Fax: +49 (0) 5 11 - 5 32 48 96
**Status:**	Final Protocol
**Version and Date:**	**Version: 1.0** **Date: 30 November 2012**

Page 1 of 77

Proprietary confidential information.
© 2012 Boehringer Ingelheim International GmbH or one or more of its affiliated companies. All rights reserved.
This document may not - in full or in part - be passed on, reproduced, published or otherwise used without prior written permission.

Proprietary confidential information © 2015 Boehringer Ingelheim International GmbH or one or more of its affiliated companies

CLINICAL TRIAL PROTOCOL SYNOPSIS

**Name of company:**<br><br>Boehringer Ingelheim		**Tabulated Trial Protocol**	
**Name of finished product:**<br><br>NA			
**Name of active ingredient:**<br><br>BI 207127 in combination with faldaprevir			
**Protocol date:**<br>30 Nov 2012	**Trial number:**<br>1241.30		**Revision date:**

**Title of trial:**	A phase II randomised, double-blind and placebo-controlled study of BI 207127 in combination with faldaprevir and ribavirin in patients with moderate hepatic impairment (Child-Pugh B) with genotype 1b chronic hepatitis C infection
**Co-ordinating:**	Michael P. Manns, Ph.D., M.D.
**Trial sites:**	Multi-centre trial
**Clinical phase:**	Ib/IIb
**Objectives:**	The objective of Cohort A is to evaluate the safety and pharmacokinetic (PK) profile of BI 207127 (potentially two doses) in combination with 120 mg once daily (q.d.) FDV and weight-based RBV in a small group of patients with moderate hepatic impairment (Child-Pugh B [CPB]) compared to patients with mild hepatic impairment (Child-Pugh A [CPA]) to define the BI 207127 dose to be used in Cohort B.<br>The objective of Cohort B is to assess efficacy, safety, and pharmacokinetics of 24-week treatment of the BI 207127 dose selected in Cohort A in combination with 120 mg once daily (q.d.) FDV and weight –based RBV in a larger group of chronically infected HCV GT1b patients with moderate hepatic impairment (CPB).
**Methodology:**	Cohort A: Open label; Cohort B: Randomised, double-blind, placebo-controlled
**No. of patients:**	
**total entered:**	Approximately 165
**each treatment:**	Cohort A (15 patients per arm)<br>Arm 1 CPA 600 mg BI 207127 b.i.d. + 120 mg FDV q.d. + RBV b.i.d.<br>Arm 2 CPB 400 mg BI 207127 b.i.d. + 120 mg FDV q.d. + RBV b.i.d.<br>Arm 3 CPB 600 mg BI 207127 b.i.d. + 120 mg FDV q.d. + RBV b.i.d.<br>Cohort B (90 active treatment, 30 placebo)<br>Arm 4 CPB BI 207127 placebo b.i.d. + FDV placebo q.d. + RBV placebo b.i.d<br>Arm 5 CPB 400 or 600 mg (based on outcome of cohort A) BI 207127 b.i.d. + 120 mg FDV q.d. + RBV b.i.d
**Diagnosis:**	Chronic HCV infection
**Main criteria for inclusion:**	Treatment naïve and experienced patients (prior relapse, interferon intolerant, and [allowed in Cohort A only] prior partial response).<br>Chronic HCV infection of genotype 1 (GT1), sub-GT1b virus only.<br>Liver cirrhosis defined as Metavir Grade=4 or Ishak Grade ≥5 on liver biopsy or liver stiffness of ≥13 kPa on fibroscan.

Proprietary confidential information.
© 2012 Boehringer Ingelheim International GmbH or one or more of its affiliated companies. All rights reserved.
This document may not - in full or in part - be passed on, reproduced, published or otherwise used without prior written permission.

Proprietary confidential information © 2015 Boehringer Ingelheim International GmbH or one or more of its affiliated companies

**Name of company:**<br><br>Boehringer Ingelheim		**Tabulated Trial Protocol**	
**Name of finished product:**<br><br>NA			
**Name of active ingredient:**<br><br>BI 207127 in combination with faldaprevir			
**Protocol date:**<br>30 Nov 2012	**Trial number:**<br>1241.30		**Revision date:**

**Test product:**	Faldaprevir (FDV)
**dose:**	120 mg q.d. (additional 120 mg loading dose for a total of 240 mg q.d. on Day 1)
**mode of admin.:**	Per os
**Test product:**	BI 207127
**dose:**	600 mg b.i.d.<br>400 mg b.i.d.
**mode of admin.:**	Per os
**Test product:**	Ribavirin
**dose:**	1000 or 1200 mg daily (weight-based b.i.d. dosing)
**mode of admin.:**	Per os
**Comparator products:**	BI 207127-matching placebo; FDV-matching placebo; ribavirin-matching placebo
**dose:**	NA
**mode of admin.:**	Per os
**Duration of treatment:**	24 weeks
**Criteria for efficacy:**	The primary endpoint is Sustained Virologic Response at Week 12 post-treatment (SVR12): Plasma HCV RNA level <25 IU/mL at 12 weeks after EOT.<br>The secondary efficacy endpoints are:<br>• SVR4: Plasma HCV RNA level <25 IU/mL at 4 weeks after EOT<br>• SVR24: Plasma HCV RNA level <25 IU/mL at 24 weeks after EOT
**Criteria for pharmacokinetics:**	Pharmacokinetic parameters for BI 207127 (and metabolites), FDV and ribavirin<br>Primary endpoints: Cmax, AUC_{0-12}, AUC_{0-24}, C12, C24
**Criteria for safety:**	Safety endpoints to be assessed in this trial include: AEs, including Division of Acquired Immunodeficiency Syndrome (DAIDS) grades, AEs leading to treatment discontinuation of all trial medications, SAEs, Events of liver disease progression, Laboratory test abnormalities by DAIDS grades, Change in laboratory test values over time.
**Statistical methods:**	A null hypothesis of equal response rate of SVR12 between the treatment and placebo group will be tested by Fisher's exact test. Time to achieving HCV RNA undetected and time to virologic failure will be analyzed using the Cox model.

Proprietary confidential information.
© 2012 Boehringer Ingelheim International GmbH or one or more of its affiliated companies. All rights reserved.
This document may not - in full or in part - be passed on, reproduced, published or otherwise used without prior written permission.

Proprietary confidential information © 2015 Boehringer Ingelheim International GmbH or one or more of its affiliated companies

FLOW CHART

Study period	Screening	Treatment												Follow-up (FU)[13]					
Visit	1	2	3	4	5	6	7	8	9	10	11	12	EOT[12]	FU1	FU2	FU3	FU4	FU5	EOO[14]
Week	-6 to -1	0	1	2	3	4	6	8	10	12	16	20	24	EOT +4	EOT +12	EOT +24	EOT +48	EOT +72	EOT +96
Day	-42 to -7	1	8 ±2	15±2	22±2	29±2	43±2	57±2	71±2	85±2	113±2	141 ±2	169 ±2	EOT+28 ±7	EOT+84 -2/+7	EOT+168 -2/+7	EOT+336 ±14	EOT+504 ±14	EOT+672 ±21
Informed consent	X																		
Demographics, medical history and baseline conditions	X																		
HCV, HBV, HIV serology	X																		
Drug screening (urine)	X																		
Eligibility criteria	X	X																	
Randomisation via IRT		X																	
Pharmacogenomic sample[1]		X																	
Pregnancy screening[2]	X	X	X	X	X	X	X	X	X	X	X	X	X	X	X	X			
Physical examination[3]	X[C]	X[T]	X[T]	X[T]	X[T]	X[T]	X[T]	X[T]	X[T]	X[T]	X[T]	X[T]	X[C]	X[C]	X[T]	X[T]	X[T]	X[T]	X[T]
12-lead ECG	X									X			X	X					
HCV RNA	X	X	X	X	X	X	X	X	X	X	X	X	X	X	X	X	X	X	X
Virology samples[4]	X	X	X	X	X	X	X	X	X	X	X	X	X	X	X	X	X	X	X
Laboratory tests[5]	X	X	X	X	X	X	X	X	X	X	X	X	X	X	X	X	X	X	X
Pre-dose PK sample[6]		X	X	X	X	X	X	X	X	X	X	X	X						
Intensive PK[7,8]		X	X			X					X								
Assess liver disease progression[9]	X	X				X		X		X			X	X	X	X	X	X	X
FibroSURE[TM]		X								X			X		X	X	X	X	X
EQ-5D and HCRU[10]		X				X		X			X		X			X			
AEs	X	X	X	X	X	X	X	X	X	X	X	X	X	X	X[16]	X[16]	X[16]	X[16]	X[16]
Concomitant medication	X	X	X	X	X	X	X	X	X	X	X	X	X	X	X	X	X	X	X
Dispense trial medication		X[11]				X		X		X	X	X							
Compliance			X	X	X	X	X	X	X	X	X	X	X						
Termination of trial medication													X[15]						
Trial completion form																			X

Proprietary confidential information.
© 2012 Boehringer Ingelheim International GmbH or one or more of its affiliated companies. All rights reserved.
This document may not - in full or in part - be passed on, reproduced, published or otherwise used without prior written permission.

Proprietary confidential information © 2015 Boehringer Ingelheim International GmbH or one or more of its affiliated companies

Footnotes:

1. One mandatory and one optional pharmacogenomic (PG) samples will be collected at Visit 2.
2. Serum pregnancy tests will be performed at all study visits. At Visit 2, an additional urine (dipstick) pregnancy test will be performed and only those female patients with a negative result will receive study medication. When visits are more than 4 weeks apart, female patients of childbearing potential will be provided with urine pregnancy tests to perform monthly pregnancy tests at home until 7 months after the last dose of ribavirin. Urine pregnancy test for male participants' female partners of child-bearing potential will be provided to perform monthly testing until 7 months after the last dose of ribavirin. Male patients will provide and ensure pregnancy testing of his female partner. It is the responsibility of the investigator to remind the male patients of this.
3. "X^C" is a complete physical examination. "X^T" is a targeted physical examination including measurements of vital signs and evaluation of organ systems particularly associated with AE(s) symptoms.
4. Plasma will be stored for analysis of genotypic and phenotypic viral resistance and other HCV viral tests.
5. For laboratory test details refer to Section 5.2.3. Patients should be fasting for at least 6 hours prior to the blood sample being taken (except screening visit).
6. Patients should not take study medication the mornings of study visits. Pre-dose PK samples for FDV, BI 207127 (and metabolites) and ribavirin will be collected during the clinic visit. Time and date of the last drug intake will be recorded for the purpose of using in PK analysis. If patient is subjected to intensive PK on same visit day, only collect intensive PK samples. For details refer to Section 5.5.2.1.
7. For all of Cohort A and Cohort B PK sub-study participants: intensive PK samples for FDV, BI 207127 (and metabolites) and ribavirin will be collected at multiple times after dosing. Refer to Appendix 10.1.2, Table 10.1.2: 1 for Cohort A and Table 10.1.2: 2 for Cohort B PK sub-study.
8. Only for Cohort A, blood samples will be collected at 2.5 hours post dose at Day 1, Day 8, Week 4, and Week 16 for determination of protein binding. Refer to Appendix 10.1.2, Table 10.1.2: 1.
9. For details on assessment of liver disease progression refer to Section 5.3.2.2.
10. EQ-5D questionnaires: patient reported outcomes. Healthcare Resource Use (HCRU): Patient outpatient visits due to AEs, hospital visits (other than study scheduled visits) due to AEs, and hospitalizations due to AEs. Refer to Section 5.3.1.2.
11. Includes an additional 120 mg loading dose of FDV (or matching placebo) for a total of 240 mg taken at the site and observed by research staff on Day 1.
12. End of Treatment (EOT). Patients who discontinue treatment prematurely must perform an EOT visit within 2 weeks of last dose of study drug. Patients who discontinue treatment due to lack of efficacy will perform the EOT visit when the lack of efficacy criteria is confirmed. Patients must proceed according to the visit schedule with the subsequent follow up visits after EOT even if they had an early treatment discontinuation.
13. FU visits are counted from day after last intake of study medication (EOT), which in most cases will be EOT visit.
14. End of Observation (EOO).
15. Termination of trial medication may be earlier if the patient does not complete the full treatment course. Each trial medication termination will be recorded separately.
16. For AE assessment after FU1, related or fatal SAEs only (cf. Section 5.2.2.2).

Proprietary confidential information.
© 2012 Boehringer Ingelheim International GmbH or one or more of its affiliated companies. All rights reserved.
This document may not - in full or in part - be passed on, reproduced, published or otherwise used without prior written permission.

Proprietary confidential information © 2015 Boehringer Ingelheim International GmbH or one or more of its affiliated companies

TABLE OF CONTENTS

TITLE PAGE ... 1
CLINICAL TRIAL PROTOCOL SYNOPSIS ... 2
FLOW CHART ... 4
TABLE OF CONTENTS ... 6
ABBREVIATIONS ... 10
1. INTRODUCTION ... 12
1.1 MEDICAL BACKGROUND ... 12
1.2 DRUG PROFILE ... 13
2. RATIONALE, OBJECTIVES, AND BENEFIT - RISK ASSESSMENT ... 16
2.1 RATIONALE FOR PERFORMING THE TRIAL ... 16
2.2 TRIAL OBJECTIVES ... 16
2.3 BENEFIT - RISK ASSESSMENT ... 16
3. DESCRIPTION OF DESIGN AND TRIAL POPULATION ... 19
3.1 OVERALL TRIAL DESIGN AND PLAN ... 19
3.1.1 Administrative structure of the trial ... 20
3.1.1.1 Data Monitoring Committee (DMC) ... 21
3.1.1.2 Rash and Photosensitivity Adjudication Committee (RPAC) 22
3.1.1.3 Internal independent data review committee ... 22
3.2 DISCUSSION OF TRIAL DESIGN, INCLUDING THE CHOICE OF CONTROL GROUPS ... 22
3.3 SELECTION OF TRIAL POPULATION ... 23
3.3.1 Main diagnosis for study entry ... 24
3.3.2 Inclusion criteria ... 24
3.3.3 Exclusion criteria ... 25
3.3.4 Removal of patients from therapy or assessments ... 27
3.3.4.1 Removal of individual patients ... 27
3.3.4.2 Discontinuation of the trial by the sponsor ... 28
4. TREATMENTS ... 29
4.1 TREATMENTS TO BE ADMINISTERED ... 29
4.1.1 Identity of BI investigational products and comparator products ... 29
4.1.2 Method of assigning patients to treatment groups ... 29
4.1.3 Selection of doses in the trial ... 30

Proprietary confidential information.
© 2012 Boehringer Ingelheim International GmbH or one or more of its affiliated companies. All rights reserved.
This document may not - in full or in part - be passed on, reproduced, published or otherwise used without prior written permission.

Proprietary confidential information © 2015 Boehringer Ingelheim International GmbH or one or more of its affiliated companies

4.1.4 Drug assignment and administration of doses for each patient 30
4.1.5 Blinding and procedures for unblinding 31
4.1.5.1 Blinding 31
4.1.5.2 Procedures for emergency unblinding...................................33
4.1.6 Packaging, labelling, and re-supply 33
4.1.7 Storage conditions...33
4.1.8 Drug accountability 34
4.2 CONCOMITANT THERAPY AND RESTRICTIONS, AND RESCUE TREATMENT...34
4.2.1 Rescue medication, emergency procedures, and additional treatments 34
4.2.2 Restrictions 37
4.2.2.1 Restrictions regarding concomitant treatment........................37
4.2.2.2 Restrictions on diet and life style 38
4.3 TREATMENT COMPLIANCE 38
5. VARIABLES AND THEIR ASSESSMENT 40
5.1 EFFICACY - CLINICAL PHARMACOLOGY..40
5.1.1 Endpoints of efficacy..40
5.1.2 Assessment of efficacy..41
5.2 SAFETY..41
5.2.1 Endpoints of safety...41
5.2.2 Assessment of adverse events..41
5.2.2.1 Definitions of adverse events 41
5.2.2.2 Adverse event and serious adverse event reporting 43
5.2.3 Assessment of safety laboratory parameters.....................................45
5.2.3.1 Pregnancy tests 48
5.2.4 Electrocardiogram 49
5.2.5 Physical examination 49
5.2.6 FibroSURE™ 49
5.3 OTHER 50
5.3.1 Other endpoints..50
5.3.1.1 Health Care Resource Utilization (HCRU) and Work Productivity 50
5.3.1.2 EQ-5D 50
5.3.2 Other assessments 51
5.3.2.1 Genotypic and Phenotypic Resistance 51
5.3.2.1.1 Genotypic Resistance 51
5.3.2.1.2 Phenotypic Resistance...51
5.3.2.1.3 Methods and timing of sample collection 51
5.3.2.2 Assessment of liver disease progression 51

Proprietary confidential information.
© 2012 Boehringer Ingelheim International GmbH or one or more of its affiliated companies. All rights reserved.
This document may not - in full or in part - be passed on, reproduced, published or otherwise used without prior written permission.

Proprietary confidential information © 2015 Boehringer Ingelheim International GmbH or one or more of its affiliated companies

5.3.3 Pharmacogenomic evaluation 52
5.3.3.1 Methods and timing of sample collection 52
5.3.3.2 Analytical determinations 53
5.4 APPROPRIATENESS OF MEASUREMENTS 53
5.5 DRUG CONCENTRATION MEASUREMENTS AND PHARMACOKINETICS 53
5.5.1 Pharmacokinetic endpoints 53
5.5.2 Methods of sample collection 54
5.5.2.1 Pharmacokinetic samples 54
5.5.2.2 Protein binding 55
5.5.3 Analytical determinations 55
5.5.3.1 Pharmacokinetics 55
6. INVESTIGATIONAL PLAN 56
6.1 VISIT SCHEDULE 56
6.2 DETAILS OF TRIAL PROCEDURES AT SELECTED VISITS 56
6.2.1 Screening and run-in periods 56
6.2.2 Treatment period 56
6.2.2.1 Randomization/treatment assignment visit (Visit 2) 57
6.2.2.2 Visit 3 to Visit 12 57
6.2.2.3 EOT Visit 57
6.2.3 End of trial and follow-up period 57
6.2.3.1 Follow-up (FU) 57
6.2.3.2 End of Observation 58
6.2.3.3 Treatment Option for Arm 4 58
7. STATISTICAL METHODS AND DETERMINATION OF SAMPLE SIZE 59
7.1 STATISTICAL DESIGN - MODEL 59
7.1.1 Design 59
7.2 NULL AND ALTERNATIVE HYPOTHESES 59
7.3 PLANNED ANALYSES 59
7.3.1 Primary analyses 60
7.3.2 Secondary analyses 60
7.3.3 Safety analyses 60
7.3.4 Interim analyses 61
7.3.5 Pharmacokinetic analyses 61
7.3.6 Other analyses 62
7.4 HANDLING OF MISSING DATA 62
7.5 RANDOMISATION 62
7.6 DETERMINATION OF SAMPLE SIZE 63

Proprietary confidential information.
© 2012 Boehringer Ingelheim International GmbH or one or more of its affiliated companies. All rights reserved.
This document may not - in full or in part - be passed on, reproduced, published or otherwise used without prior written permission.

Proprietary confidential information © 2015 Boehringer Ingelheim International GmbH or one or more of its affiliated companies

8. INFORMED CONSENT, DATA PROTECTION, TRIAL RECORDS 64
8.1 STUDY APPROVAL, PATIENT INFORMATION, AND INFORMED CONSENT 64
8.2 DATA QUALITY ASSURANCE 64
8.3 RECORDS 65
8.3.1 Source documents 65
8.3.2 Direct access to source data and documents 65
8.4 LISTEDNESS AND EXPEDITED REPORTING OF ADVERSE EVENTS 65
8.4.1 Listedness 65
8.4.2 Expedited reporting to health authorities and IECs / IRBs 65
8.5 STATEMENT OF CONFIDENTIALITY 66
9. REFERENCES 67
9.1 PUBLISHED REFERENCES 67
9.2 UNPUBLISHED REFERENCES 67
10. APPENDICES 68
10.1 PHARMACOKINETIC METHODS 68
10.1.1 Evaluation of Pharmacokinetic Parameters 68
10.1.2 Blood sampling scheme for Pharmacokinetics 70
10.2 DERMATOLOGY MANAGEMENT PLAN 72
10.2.1 Introduction and scope 72
10.2.2 General measures for skin protection 72
10.2.3 Drug-induced skin rash 72
10.2.3.1 Definition of severity for skin rash* 72
10.2.3.2 Management guidelines of skin rash 73
10.2.4 Photosensitivity reactions 74
10.2.4.1 Definition of severity of photosensitivity reactions 74
10.2.4.2 Management guidelines for photosensitivity reactions 74
10.3 CHILD-TURCOTTE-PUGH CLASSIFICATION 76
11. DESCRIPTION OF GLOBAL AMENDMENTS 77

Proprietary confidential information.
© 2012 Boehringer Ingelheim International GmbH or one or more of its affiliated companies. All rights reserved.
This document may not - in full or in part - be passed on, reproduced, published or otherwise used without prior written permission.

Proprietary confidential information © 2015 Boehringer Ingelheim International GmbH or one or more of its affiliated companies

ABBREVIATIONS

AE	Adverse Event
ALT	Alanine Aminotransferase
AST	Aspartate Aminotransferase
AUC_{0-12}	Area under the concentration-time curve in plasma over the time interval from 0 to 12 hour
AUC_{0-24}	Area under the concentration-time curve in plasma over the time interval from 0 to 24 hour
BI	Boehringer Ingelheim
b.i.d.	bis in die (twice daily)
CA	Competent Authority
CI	Confidence Interval
C_{max}	Maximum concentration of an analyte in plasma or serum at steady state
CML	Local Clinical Monitor
CPA	Child-Pugh A
CPB	Child-Pugh B
CRA	Clinical Research Associate
CRF	Case Report Form
CTMF	Clinical Trial Master File
CTP	Clinical Trial Protocol
CTR	Clinical Trial Report
CYP	Cytochrome P450
DAA	Direct Acting Antiviral
DAIDS	Division of Acquired Immunodeficiency Syndrome
DRESS	Drug Rash with Eosinophilia and Systemic Symptoms
DMC	Data Monitoring Committee
eCRF	Electronic Case Report Form
EOO	End of Observation
EOT	End of Treatment
ESA	Erythropoiesis Stimulating Agent
$ETR_{\mathbf{TND}}$	End of Treatment Response
EudraCT	European Clinical Trials Database
FDV	Faldaprevir
FU	Follow-up
GCP	Good Clinical Practice
G-CSF	Granulocyte-Colony Stimulating Factor
GI	Gastrointestinal
GT1	Genotype 1
Hb	Haemoglobin
HCV	Hepatitis C Virus
HCRU	Health Care Resource Utilization
IB	Investigator’s Brochure
IEC	Independent Ethics Committee
INR	International Normalized Ratio
IRB	Institutional Review Board

Proprietary confidential information.
© 2012 Boehringer Ingelheim International GmbH or one or more of its affiliated companies. All rights reserved.
This document may not - in full or in part - be passed on, reproduced, published or otherwise used without prior written permission.

Proprietary confidential information © 2015 Boehringer Ingelheim International GmbH or one or more of its affiliated companies

IRT	Interactive Response Tool
ISF	Investigator Site File
LFT	Liver function test
MedDRA	Medical Dictionary for Drug Regulatory Activities
MRI	Magnetic resonance imaging
OPU	Operative Unit
PD	Pharmacodynamics
PegIFN	Pegylated alfa interferon
P-gp	P-glycoprotein
PI	Principal Investigator
PK	Pharmacokinetic
q.d.	quaque die (once daily)
RBC	Red blood cell
RBV	Ribavirin
RNA	Ribonucleic acid
RPAC	Rash and Photosensitivity Adjudication Committee
SAE	Serious Adverse Event
SOC	Standard of Care
SOP	Standard Operating Procedure
SUSAR	Suspected Unexpected Serious Adverse Reaction
SVR	Sustained virological response
$t_{1/2}$	Terminal half-life of the analyte in plasma/serum
TCM	Trial Clinical Monitor
TEN	Toxic epidermal necrolysis
t.i.d.	ter in die (three times daily)
t_{max}	Time to peak concentration
TSAP	Trial Statistical Analysis Plan
ULN	Upper limit normal
UV	Ultraviolet
WHO	World Health Organization
$W12U_{TND}$	Plasma HCV undetected at Week 12
W4U	Plasma HCV RNA level <25 IU/mL at Week 4
$W4U_{TND}$	Plasma HCV RNA undetected at Week 4

Proprietary confidential information.
© 2012 Boehringer Ingelheim International GmbH or one or more of its affiliated companies. All rights reserved.
This document may not - in full or in part - be passed on, reproduced, published or otherwise used without prior written permission.

Proprietary confidential information © 2015 Boehringer Ingelheim International GmbH or one or more of its affiliated companies

1. INTRODUCTION

1.1 MEDICAL BACKGROUND

Chronic infection with hepatitis C virus (HCV) represents a major public health problem worldwide. The World Health Organization (WHO) estimates that approximately 3% of the world population are infected. Approximately one third of those infected will develop cirrhosis in less than 20 years, and as many as 7% may develop hepatocellular carcinoma (P00-15027).

Different HCV genotypes prevail in different geographic regions, with genotype 1 (GT1) being the predominant genotype (70%) in United States, Asia, and Europe. Sub-GT1a is more prevalent in North and South America and common in Australia, while sub-GT1b is more prevalent in Europe and Asia.

Currently, two direct acting antiviral agents (DAAs) (HCV protease inhibitors, telaprevir and boceprevir) have been approved for HCV treatment in selected countries. Treatment with 12 weeks of telaprevir in combination with pegylated alfa interferon (PegIFN) and ribavirin (RBV) for either 24 or 48 weeks demonstrated sustained virologic response (SVR) rate of 74% or 79% at Week 24 (ILLUMINATE or ADVANCE studies, R12-0262). Boceprevir given for 24 or 44 weeks in combination with PegIFN/RBV achieved SVR rates of 63% to 66%, respectively (SPRINT-2 study, R12-0263). These combination therapies represent the new Standard of Care (SOC) for HCV patients with chronic GT1 infection with compensated liver disease (mild hepatic impairment or CPA). However, both these compounds are given three times daily (t.i.d.), are associated with substantial side effects, and are not approved regimens for patients with moderate hepatic impairment (CPB).

Despite the improved virologic response rates in patients with HCV GT1 infection following the recent approvals of new DAAs, the increasingly aging population with co-morbidities and contraindications to PegIFN, and the limiting side effects of this current SOC underscores the remaining urgent need for more effective, less toxic, shorter, more convenient and less invasive therapy for these patients. Effective antiviral therapy without PegIFN would constitute a major breakthrough in HCV treatment. PegIFN is contraindicated in CPB patients since it is poorly tolerated and associated with serious adverse events, high discontinuation rates and a faster liver progression in this population. Therefore, this patient population has a high unmet medical need and an interferon-free regimen may represent the only treatment option for patients with progressive liver cirrhosis or with HCV re-infection after liver transplantation.

BI is investigating the safety and efficacy of BI 207127, an oral, specific and reversible non-nucleoside HCV specific ribonucleic acid (RNA)-dependent RNA polymerase inhibitor, in combination with faldaprevir (FDV), a potent, oral HCV NS3/4a protease inhibitor, and RBV in patients with moderate hepatic impairment.

Proprietary confidential information.
© 2012 Boehringer Ingelheim International GmbH or one or more of its affiliated companies. All rights reserved.
This document may not - in full or in part - be passed on, reproduced, published or otherwise used without prior written permission.

Proprietary confidential information © 2015 Boehringer Ingelheim International GmbH or one or more of its affiliated companies

1.2 DRUG PROFILE

Detailed descriptions of drug substance, pharmacology, safety, pharmacology, toxicology and pharmacokinetics from preclinical and clinical studies are available in the current version of the Investigator's Brochures (IB) for BI 207127 (U06-1740) and FDV (U04-3332).

Faldaprevir (FDV)

FDV (also known as BI 201335) is a potent and specific HCV NS3/4A protease inhibitor. FDV is currently being evaluated in combination with PegIFN and RBV in Phase 3 of clinical development.

Clinical drug interaction studies of FDV with CYP3A4/5, CYP2C9 and P-gp have shown moderate inhibition of CYP3A4 and mild inhibition of CYP2C9. These drug interactions can be effectively managed. Specific recommendations for co-medications, including substrates of CYP3A4/5 or CYP2C9 which have a narrow therapeutic range, were developed with external clinical pharmacology experts and are provided in the Investigator Site File (ISF) (lists of restricted concomitant drugs and of drugs that need to be used with caution). For more information refer to the current IB version (U04-3332).

BI 207127

BI 207127 is a specific and reversible non-nucleoside inhibitor of HCV specific RNA-dependent RNA polymerase and is being developed in combination with FDV. Phase 2b evaluation of BI 207127 is ongoing in trial 1241.21 (SOUND-C studies) with Parts 1 and 2 complete and Part 3 in progress.

1241.21 Part 2 (SOUND-C 2) is a 5-arm, open-label, randomized, phase 2b study evaluating efficacy and safety of interferon-free oral combination regimens of BI 207127 and FDV with and without RBV for up to 40 weeks of treatment. A total of 362 treatment-naïve HCV GT1 patients were randomized and treated in 5 treatment arms. In the combined interim analysis by viral subtype (1a *vs.* 1b), the highest SVR12 (85%) was achieved with the twice daily (BID) 28-week regimen in GT1b infected patients (Table 1.2: 1). This is the dose and target population to be studied in the phase 3 program starting in parallel. In contrast, GT1a, and especially the sub-population of 1a_non-CC achieved sub-optimal SVR12 rates ranging from 42% to 0% (Table 1.2: 1). Among GT1b patients with compensated liver cirrhosis in SOUND-C2 randomized to RBV-containing regimens, the SVR rate was between 57% and 80%.

Proprietary confidential information.
© 2012 Boehringer Ingelheim International GmbH or one or more of its affiliated companies. All rights reserved.
This document may not - in full or in part - be passed on, reproduced, published or otherwise used without prior written permission.

Proprietary confidential information © 2015 Boehringer Ingelheim International GmbH or one or more of its affiliated companies

Table 1.2: 1 1241.21 SOUND-C2 SVR12 rate by patient sub-population

	GT1b	GT1a
16-week BI 207127 TID with FDV and RBV	35/47 (75%)	13/34 (38%)
28-week BI 207127 TID with FDV and RBV	33/48 (69%)	14/32 (44%)
40-week BI 207127 TID with FDV and RBV	24/43 (56%)	16/34 (47%)
28-week BI 207127 BID with FDV and RBV	41/48 (85%)	13/30 (43%)
28-week BI 207127 TID FDV without RBV	16/28 (57%)	2/18 (11%)

Among patients assigned to a BI 207127 600 mg t.i.d. regimen, patients randomised to 40 weeks experienced the highest rate of discontinuation due to adverse events (AEs). There was a 5%, 13% and 25% rate of early discontinuation due to AEs in the 16-week, 28-week and 40-week treatment arms, respectively. In the BI 207127 600 mg b.i.d. 28-week regimen, 8% patients discontinued due to AEs. As expected, the most frequent AEs reported on the FDV/ BI 207127 combination therapy are skin (rash, photosensitivity reactions) and gastrointestinal (GI) (nausea, vomiting and diarrhoea) events and indirect hyperbilirubinemia. Photosensitivity reactions and vomiting occurring during the first 10 days of treatment have been statistically associated with the extent of the exposure of the BI drugs.

BI 207127 and FDV plasma concentrations were higher when the two drugs are used in combination compared to when there are used as monotherapy or when combined with PegIFN and RBV. On-treatment, pre-dose plasma concentrations of both appeared to steadily decrease over the first few weeks of treatment reaching stable trough exposure approximately at treatment Week 4-6. In addition, a 2-fold increase in exposure of both FDV and BI 207127 in cirrhotic patients compared to non-cirrhotic patients was observed.

Drug metabolism, transport, and drug-drug interactions

Two major metabolites of BI 207127 were identified in humans, CD 6168 (alkene reduction) and BI 208333 (BI 207127-acyl glucuronide), present in plasma at ~ 32 and 23% of BI 207127 related material. CD 6168 is formed in the GI tract, likely by gut bacteria and BI 208333 is formed by UGT1A1, 1A3, 1A7 and 1A8, of which UGT1A1 contribution appears to be predominant.

BI 207127 is a substrate of hepatic uptake transporters OATP1B1, OATP1B3 and OATP2B1. BI 207127 is also a substrate of hepatic and intestinal efflux transporters P-glycoprotein (P-gp) and BCRP.

BI 201335 is a substrate of CYP3A4, which metabolizes it to two hydroxy metabolites M2a and M2b. While together these were found to constitute up to 40% of the dose, M2A and M2b were predominantly found only in feces. The exposure of these metabolites in plasma was very low (<1% of total BI 201335 related material). BI 201335 is a substrate of hepatic uptake transporters OATP1B1, OATP1B3 and OATP2B1.

Proprietary confidential information.
© 2012 Boehringer Ingelheim International GmbH or one or more of its affiliated companies. All rights reserved.
This document may not - in full or in part - be passed on, reproduced, published or otherwise used without prior written permission.

Proprietary confidential information © 2015 Boehringer Ingelheim International GmbH or one or more of its affiliated companies

BI 201335is also a substrate of hepatic and intestinal efflux transporters P-gp and MRP2.

Drug-drug interactions are predicted (based on in vitro DDI data) to be likely if BI 207127 is co-administered with drugs that are substrates of Cytochrome P450 (CYP)1A2, CYP2C8 and P-gp. BI 207127 is also a substrate and an inhibitor of OATP1B1, OATP1B3 and OATP2B1. Until the clinical relevance of these *in vitro* findings are confirmed *in vivo*, patients taking drugs that are CYP1A2 or CYP2C8 substrates with narrow therapeutic indices or sensitive OATP substrates (such as certain statins) are either restricted from concomitant use or recommended for use at the lowest effective doses. The clinical drug interaction study results (1241.18) indicate that inhibition of CYP2C9 and CYP3A4 observed in vitro is not clinically significant.

Proprietary confidential information.
© 2012 Boehringer Ingelheim International GmbH or one or more of its affiliated companies. All rights reserved.
This document may not - in full or in part - be passed on, reproduced, published or otherwise used without prior written permission.

Proprietary confidential information © 2015 Boehringer Ingelheim International GmbH or one or more of its affiliated companies

2. RATIONALE, OBJECTIVES, AND BENEFIT - RISK ASSESSMENT

2.1 RATIONALE FOR PERFORMING THE TRIAL

Patients with compensated liver cirrhosis have been included and successfully treated in SOUND-C2 with BI 207127 in combination with FDV and RBV. Trial 1241.30 will assess this combination treatment in patients with decompensated cirrhosis, a patient population with a high unmet medical need and no available antiviral treatment options, as PegIFN is contraindicated. This study will aim to define an interferon-free regimen that may offer an antiviral treatment option for patients chronically infected HCV GT1b with moderate hepatic impairment (CPB).

2.2 TRIAL OBJECTIVES

The objective of Cohort A is to evaluate the safety and pharmacokinetic (PK) profile of BI 207127 (potentially two doses) in combination with 120 mg once daily (q.d.) FDV and weight-based RBV in a small group of patients with moderate hepatic impairment (CPB) compared to patients with mild hepatic impairment (CPA) to define the BI 207127 dose to be used in Cohort B.

The objective of Cohort B is to assess efficacy, safety, and pharmacokinetics of 24-week treatment of the BI 207127 dose selected in Cohort A in combination with 120 mg once daily (q.d.) FDV and weight –based RBV in a larger group of chronically infected HCV GT1b patients with moderate hepatic impairment (CPB).

2.3 BENEFIT - RISK ASSESSMENT

Patients participating in this study have a high unmet medical need with their only available treatment option for decompensated cirrhosis being liver transplantation. Once cirrhosis has developed, mortality is approximately 10% over the subsequent 10 years. Clinical hepatic decompensation occurs in 30% of patients with cirrhosis and 5-year survival declines to about 50% (P97-2059).

The inclusion of treatment naïve patients is supported by data from the SOUND-C2 trial, the largest phase 2 trials with all-oral HCV treatment performed to date. Efficacy and safety in patients with compensated liver cirrhosis was studied in SOUND-C2 and the BI 207127 600 mg b.i.d. dose regimen demonstrated the best benefit/risk ratio in this population. Among GT1b patients with cirrhosis randomised to the b.i.d. group, the SVR12 rate was 80% and the rate of AEs leading to discontinuation of treatment was similar to the overall patients in same arm (approximately 8%). Most AEs were of mild intensity; the most common AEs were rash, photosensitivity reactions, asthenia, fatigue, jaundice (isolated indirect hyperbilirubinemia), nausea, vomiting and diarrhoea.

Many patients with progressive liver cirrhosis have been exposed to PegIFN/RBV in the past. Thus, to secure sufficient enrolment in this important study for a high-medical need

Proprietary confidential information.
© 2012 Boehringer Ingelheim International GmbH or one or more of its affiliated companies. All rights reserved.
This document may not - in full or in part - be passed on, reproduced, published or otherwise used without prior written permission.

Proprietary confidential information © 2015 Boehringer Ingelheim International GmbH or one or more of its affiliated companies

population treatment experienced patients (prior relapse, interferon intolerant, and [allowed in Cohort A only] prior partial response) will also be included (cf. Section 3.3.2 for definitions).

This trial will include a PK sub-study for Cohort B to further assess the BI 207127 b.i.d. dose selected in Cohort A in a larger group of CPB patients. Refer to Section 4.1.3 for selection of dose rationale. Rash and photosensitivity reaction were reported in 20-30% of patients. Rash was commonly reported as maculo-papular skin reaction on the trunk and extremities, while photosensitivity reactions were described as exaggerated sunburns in hands, face or any other sun-exposed skin area. Among patients who received 600 mg b.i.d. BI 207127 in combination with FDV and RBV, all skin events were mild. There were no moderate, severe, or serious skin events or discontinuation of treatment due to skin events reported. Serious rash and photosensitivity reactions were only reported with higher BI 207127 dose (600 mg tid) and none of the cases were considered life-threatening. There were no cases of Steven-Johnson, erythema multiforme, toxic epidermal necrolysis (TEN) or drug rash with eosinophilia and systemic symptoms (DRESS) reported in any of the BI 207127-FDV combination trials to date. Rash and photosensitivity reactions will be carefully monitored and managed with a detailed dermatologic management plan (cf. Appendix 10.2). The dermatologic plan includes precautionary measures to restrict exposure to sun and artificial ultraviolet (UV) light.

GI events such as nausea, vomiting and diarrhoea were reported in BI 207127-FDV combination trials. The highest risk of vomiting in previous trials was observed during the first 2 days of treatment (17% cumulative risk of vomiting during the first 2 days) (U11-3471-01). There was an association between the high BI 207127 exposure at Day 1 and the risk of GI events (U11-3472-01). This may have been due to the administration of an induction dose of BI 207127 (1200 mg, 2 times the individual dose) on the morning of first day of dosing. Since there was no virologic benefit associated with the induction dose (no association between BI 207127 exposure and the initial HCV RNA decay kinetics), this induction dose will not be used in this trial. As such, the risk of vomiting during the first 2 days of treatment in this trial may be lower than in previous trials. PegIFN-free regimens of BI 207127 in Trial 1241.21 Parts 1 and 2 did not cause any relevant changes from baseline in laboratory parameters, except for a continuous drop in alanine aminotransferase (ALT) in all patients, a decrease in haemoglobin (Hb, consistent with RBV treatment) and increase in total bilirubin due primarily to increases in the unconjugated fraction (a known clinically benign effect of FDV treatment). Up to 25% of patients experienced jaundice due to increases in unconjugated bilirubin without concomitant increases in liver function tests (LFTs) or any sign of liver toxicity.

Of note, only approximately 10% of patients experienced anaemia (defined as Hb > 10g/dL) receiving the oral combination in SOUND-C2 compared to 36% of receiving telaprevir with PegIFN/RBV (R12-0262) or 49% of receiving boceprevir with PegIFN/RBV (R12-0263). This may be explained by the lack of PegIFN and its myelosuppressive effect which enhances the negative effect of RBV in the regimen.

Based on the current understanding that cirrhotic patients require longer treatment durations and the lack of substantial clinical data in cirrhotic patients treated for 16 weeks in phase 2, patients with compensated cirrhosis will receive treatment for 24 weeks.

Proprietary confidential information.
© 2012 Boehringer Ingelheim International GmbH or one or more of its affiliated companies. All rights reserved.
This document may not - in full or in part - be passed on, reproduced, published or otherwise used without prior written permission.

Proprietary confidential information © 2015 Boehringer Ingelheim International GmbH or one or more of its affiliated companies

A potential risk with a PegIFN-free regimen is the selection of mutations conferring resistance to both compound classes which may limit future treatments options. However, it is currently not known if HCV resistant variants are archived and persist. Therefore it might be possible to retreat patients that had selected for resistance mutation on a given DAA(s) with the same regiment once the variants are no longer detectable. Additionally, major resistant variants associated with the use of BI 207127, such as P495L, would only confer resistance to another thumb site-1 nonnucleoside NS5B inhibitor. There are currently a number of DAAs with different MOA in development (including: thumb site-2 NS5B inhibitors, NS5A inhibitors, nuclotide NS5B inhibitors, cyclophlin inhibitors, etc.). Future treatment options will be expanded once some of these agents become available to patients in the near future. To maximize the chance to achieve SVR and reduce the chance of emergence of resistance only GT1b patients (which have the highest rate of response to this regimen) will be included.

Patients with decompensated cirrhosis randomised to placebo will not receive benefit from participating in the trial. However, the knowledge gained from this treatment group will be invaluable in understanding the emergence of AEs in decompensated cirrhotic patients during placebo treatment, and thus understanding of the safety profile of the tested regimen in this population. These patients currently have no HCV treatment options and participation in this trial will not compromise future treatment options for these patients. Patients randomised to receive placebo will be given the option of receiving Arm 5 treatment after completion of 24 weeks, if still eligible for treatment (cf. Section 3.1). In addition, an independent Data Monitoring Committee (DMC) will be established (cf. Section 3.1.1.1) and all patients will be frequently monitored. If the risk-benefit ratio changes upon favourable on-treatment clinical data in the active treatment arm 5 of Cohort B, the DMC may recommend that placebo arm patients are offered the option of receiving active treatment prior to completion of 24 weeks of placebo.

Other risks to the patients are the risks inherent to any clinical trial, such as unexpected adverse clinical or laboratory events. Patients will be closely monitored for AEs and rebounds of plasma HCV RNA.

Although rare, a potential for drug-induced liver injury (DILI) is under constant surveillance by sponsors and regulators. Therefore, this study requires timely detection, evaluation, and follow-up of laboratory abnormalities of selected liver laboratory parameters to ensure patients' safety.

Given the good safety profile of this PegIFN-free combination treatment, coupled with rapid and potent antiviral activity observed so far, the sponsor assesses benefit risk relationship in this study as positive, and the knowledge gained will outweigh the potential risk for this patient population with an unmet medical need.

Proprietary confidential information.
© 2012 Boehringer Ingelheim International GmbH or one or more of its affiliated companies. All rights reserved.
This document may not - in full or in part - be passed on, reproduced, published or otherwise used without prior written permission.

Proprietary confidential information © 2015 Boehringer Ingelheim International GmbH or one or more of its affiliated companies

3. DESCRIPTION OF DESIGN AND TRIAL POPULATION

3.1 OVERALL TRIAL DESIGN AND PLAN

This is a multi-centre and multi-staged trial. Interim data from Cohort A (open label) will be used to select the BI 207127 dose to be assessed in Cohort B (double-blind, and placebo controlled, randomised with a 1:3 ratio for Arm 4 and Arm 5) CPB patients.

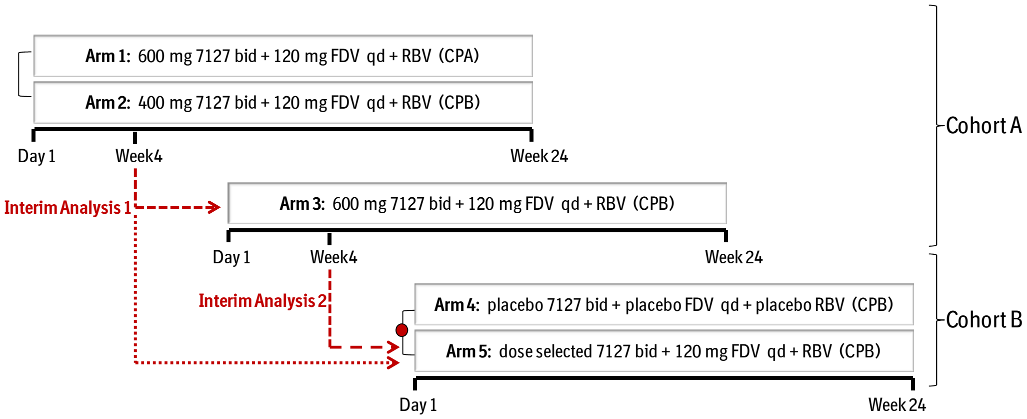

Figure 3.1: 1 Trial design

In total, approximately 165 patients are planned for inclusion in this trial. Refer to Table 3.1: 1 for details of HCV sub-type and Child Pugh score for each treatment arm.

Table 3.1: 1 Treatment arm descriptions

		HCV sub-type	Child Pugh Score	Blinding	n	Treatment*
**Cohort A**	**Arm 1**	GT1b	CPA	open label	15	600 mg BI 207127 b.i.d. + 120 mg FDV q.d. + RBV
	**Arm 2**	GT1b	CPB	open label	15	400 mg BI 207127 b.i.d. + 120 mg FDV q.d. + RBV
	**Arm 3**	GT1b	CPB	open label	15	600 mg BI 207127 b.i.d. + 120 mg FDV q.d. + RBV
**Cohort B**	**Arm 4**	GT1b	CPB	double-blind	30	placebo BI 207127 b.i.d. + placebo FDV q.d. + placebo RBV
	**Arm 5**	GT1b	CPB	double-blind	90	dose selected of BI 207127 b.i.d. + 120 mg FDV q.d. + RBV

* FDV loading dose of 240 mg total on Day 1

Patients are included in the study once they have signed the informed consent. Patients suitable after screening will be eligible to participate in the 24 week treatment period and, according to Figure 3.1: 2 will be assigned to an arm open to enrolment depending on the current screening stage of the trial.

Proprietary confidential information.
© 2012 Boehringer Ingelheim International GmbH or one or more of its affiliated companies. All rights reserved.
This document may not - in full or in part - be passed on, reproduced, published or otherwise used without prior written permission.

Proprietary confidential information © 2015 Boehringer Ingelheim International GmbH or one or more of its affiliated companies

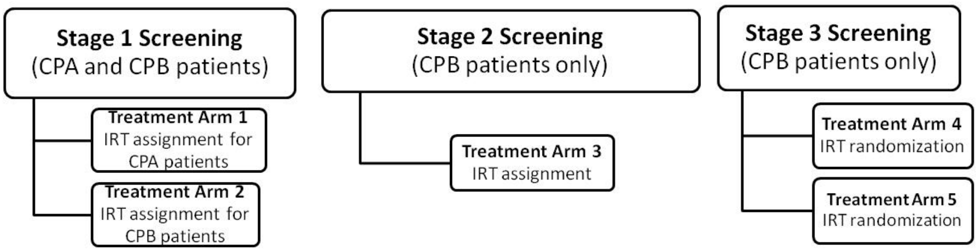

Figure 3.1: 2 Assignment of treatment arm by trial screening stage

Patients randomised to Arm 4 (placebo) will be given the option of receiving active treatment (as in Arm 5) after completion of their individual 24 week placebo treatment, if still eligible for treatment, or sooner, if results from ongoing trial as evaluated by the DMC support this (cf. Section 3.1.1.1 and ISF).

Forty eight patients in Cohort B will be assigned via Interactive Response Tool (IRT) to participate in a PK sub-study (cf. Section 5.5).

"On-treatment" AEs are those AEs that begin during treatment with the trial medications or within 28 days of the End of Treatment (EOT) visit. For more details on safety assessments in this trial, cf. Section 5.2 and Section 7.3.3.

Upon EOT visit patients will complete an additional 96 weeks follow-up (FU) period. Individual patient participation is concluded when the patient has completed the last planned visit.

The end of the trial is defined as "last patient out", i.e. last scheduled visit completed by last patient.

3.1.1 Administrative structure of the trial

The trial is sponsored by Boehringer Ingelheim.

Boehringer Ingelheim (BI) will appoint a Trial Clinical Monitor (TCM), responsible for coordinating the activities required in order to manage the trial in accordance with applicable regulations and internal standard operating procedures (SOPs), directing the clinical trial team in the preparation, conduct, and reporting of the trial, order the materials as needed for the trial, ensures appropriate training and information of Local Clinical Monitors (CML), Clinical Research Associates (CRAs), and investigators of participating countries.

Data Management and Statistical evaluation will be performed by BI according to BI SOPs. For these activities, a Trial Data Manager and a Trial Statistician will be appointed.

Tasks and functions assigned in order to organise, manage, and evaluate the trial will be defined according to BI SOPs. A list of responsible persons will be given in the Clinical Trial Master File (CTMF) document.

Proprietary confidential information.
© 2012 Boehringer Ingelheim International GmbH or one or more of its affiliated companies. All rights reserved.
This document may not - in full or in part - be passed on, reproduced, published or otherwise used without prior written permission.

Proprietary confidential information © 2015 Boehringer Ingelheim International GmbH or one or more of its affiliated companies

The organisation of the trial in the participating countries will be done by the respective local BI-organisation (Operative Unit (OPU)) or by a Contract Research Organisation (CRO) with which the responsibilities and tasks will have been agreed and a written contract filed before initiation of the clinical trial. In each OPU participating in this study, a CML will be appointed responsible for coordinating the activities required in order to manage the trial in accordance with applicable regulations and internal SOPs in the countries covered by the respective BI OPU.

A Co-ordinating Investigator will be nominated to coordinate investigators at different sites participating in this multicentre trial. Tasks and responsibilities for the Co-ordinating Investigators will be defined in a contract filed before initiation of the trial.

Documents on participating (Principal) investigators and other important study personnel, especially their curricula vitae, will be filed in the CTMF.

Details of the trial supplies including responsible institutions are given in Section 4 of this protocol.

The ISF will be maintained at the sites as required by local regulation and BI-SOP. A copy of the ISF documents will be kept as an electronic CTMF document according to BI SOPs.

A central laboratory service and IRT will be used in this trial.

3.1.1.1 Data Monitoring Committee (DMC)

The study will have a DMC independent from the sponsor. The purpose of the DMC is to ensure that the welfare of patients participating in this trial is maintained by:

- Monitoring the trial for possible untoward harmful effects or unexpected frequency of adverse safety events of study drugs;
- Assessing whether the goals of the trial are unlikely to be achieved, based on emerging data.

The DMC will evaluate the unblinded accrued patient data in order to recommend whether the trial or program should continue, be modified or stopped for safety concerns or ethical reasons. The DMC will review pertinent trial data, including deaths, serious adverse events (SAEs) and AEs, laboratory data and efficacy markers (HCV RNA). Unless specified differently in the DMC charter, it is expected that the first DMC meeting will occur approximately 2 months after the first patient has entered the trial and then every 3-4 months thereafter, with more frequent reviews as deemed necessary by the DMC or the sponsor. The DMC will review Cohort A Week 4 data prior to the start of Cohort B. All materials provided to the DMC are confidential. The tasks and responsibilities of the DMC will be filed in a charter before initiation of the trial and will contain written operating procedures. The DMC will maintain written records of all its meetings. Sponsor will remain blinded to these ongoing data reviews.

Proprietary confidential information.
© 2012 Boehringer Ingelheim International GmbH or one or more of its affiliated companies. All rights reserved.
This document may not - in full or in part - be passed on, reproduced, published or otherwise used without prior written permission.

Proprietary confidential information © 2015 Boehringer Ingelheim International GmbH or one or more of its affiliated companies

3.1.1.2 Rash and Photosensitivity Adjudication Committee (RPAC)

The study will include an independent committee of dermatology experts responsible for evaluation of protocol-defined significant skin AEs (cf. Section 5.2.2.1). The tasks and responsibilities for this committee will be established by contract before initiation of the trial and will contain written operating procedures.

3.1.1.3 Internal independent data review committee

The study will include a committee of sponsor members that are independent from the trial team. This committee will evaluate interim analysis data, including PK data, to select the BI 207127 dose for Cohort B. Refer to Section 7.3.4 for interim analysis plan and dose selection criteria.

The first interim analysis will be performed on Arm 1 and Arm 2 data up to Week 4 for submission to the committee. The BI 207127 dose for Cohort B will be selected or it will be determined that it is necessary to conduct Arm 3. If Arm 3 is needed, a second interim analysis will be performed on Arm 1 and Arm 3 data up to Week 4 for submission to the committee for Cohort B dose evaluation. Thus, randomisation in Cohort B will begin based on the Week 4 interim data of Cohort A.

The tasks and responsibilities for the Internal Independent Data Review Committee will be established before initiation of the trial and will contain written operating procedures.

3.2 DISCUSSION OF TRIAL DESIGN, INCLUDING THE CHOICE OF CONTROL GROUPS

The CPB patient population was selected because these patients, despite having the highest urgency for treatment, currently have no HCV treatment options and therefore have a high unmet medical need. Patients with poorly controlled ascites are excluded due to common fluctuations in ascites i.e. the distribution volume which could interfere with the PK analysis of both compounds.

The CPA control group was chosen because there is limited PK data in CPA patients in the phase II program for BI 207127.

Due to the low SVR rate seen in phase 2 studies in GT1a patients (cf. Table 1.2: 1), these patients are excluded from the program until a more effective treatment regimen has been identified.

The study is double-blind in order to eliminate the reporter/observer bias for the assessment of safety and of the treatment duration on efficacy between treatment Arm 4 and Arm 5.

The Arm 4 placebo group will allow comparison of safety between active treatment and placebo. It will be informative to assist in the identification of AEs related to active triple therapy versus symptoms associated with the underlying diseases and co-morbidities in CPB patients. The CPB placebo group is ethically acceptable since this population currently does not have an antiviral treatment option and their participation will be closely monitored by the

Proprietary confidential information.
© 2012 Boehringer Ingelheim International GmbH or one or more of its affiliated companies. All rights reserved.
This document may not - in full or in part - be passed on, reproduced, published or otherwise used without prior written permission.

Proprietary confidential information © 2015 Boehringer Ingelheim International GmbH or one or more of its affiliated companies

DMC. The duration of the 24 week placebo treatment may be potentially shortened and active treatment offered, if results from ongoing trial change the risk benefit ratio as evaluated by the DMC.

The smaller sample size of the PBO group was selected as a compromise between the expectation of spontaneous symptoms and (S)AE of patients due to underlying severe liver disease and the withholding of active treatment from such patients with high treatment need. It is recognised that double-blinding of such a treatment group will be challenging because of multiple laboratory test changes, specifically viral load, bilirubin, liver enzymes and hematologic changes that are related to the administration of active triple therapy vs. no such changes in patients in Arm 4. Nevertheless, extensive efforts will be made to overcome these difficulties through blinding selected laboratory tests for Arm 4 and Arm 5 to investigator, patient and sponsor (cf. Section 4.1.5.1).

PegIFN is not used in the trial and therefore, contraindications to use of PegIFN do not apply as exclusions in the eligibility criteria. Patients with retinopathies, history of psychiatric diseases, autoimmune diseases, thrombocytopenia and other conditions that contraindicate the use of PegIFN, may be entered in the study as long as their co-morbidities are considered clinically stable and with appropriate treatment, if necessary. This will ensure that the trial population is as representative as possible of the patient population seen in clinical practice.

Extended 96-week FU period will allow assessment of the persistence of the virological response, assessment of the progression of the liver disease and resistance profile of HCV over time. In case of re-detection of HCV RNA during the FU period, assessment will be made whether this represents a relapse or a subsequent re-infection.

3.3 SELECTION OF TRIAL POPULATION

A sufficient number of patients will be screened for the trial to ensure that approximately 165 patients are randomised to trial treatment (15 patients in each treatment Arm 1, Arm 2, and Arm 3; 30 patients in Arm 4, and 90 patients in Arm 5).

Demographic information, including patient gender, race and ethnicity, will be collected at screening.

Treatment Arm 1, Arm 2 and Arm 3 will be stratified by gender with a minimum of 8 females per arm (cf. Section 7.5).

Screening of patients for this trial is competitive, i.e. screening for the trial will stop at all centres when such a number of patients has been screened that it is anticipated that a sufficient number of patients will be randomised to trial treatment. This process of competitive screening will be carried out independently for each screening stage (cf. Figure 3.1: 2).

For Cohort A, patient replacement will be permitted (i.e. those patients who were assigned to a treatment group but did not receive any study drug will be replaced and those that discontinued study drug prior to Week 4 may be replaced as needed to ensure sufficient PK sample size).

Proprietary confidential information.
© 2012 Boehringer Ingelheim International GmbH or one or more of its affiliated companies. All rights reserved.
This document may not - in full or in part - be passed on, reproduced, published or otherwise used without prior written permission.

Proprietary confidential information © 2015 Boehringer Ingelheim International GmbH or one or more of its affiliated companies

For Cohort B, patients assigned to a treatment group who discontinue from the trial after start of treatment will not be replaced (i.e. those patients who were assigned to a treatment group but did not receive any study drug will be replaced to ensure that patients will receive study drug in each treatment group).

Re-screening of patients will not be permitted.

A log of all patients included into the study (i.e. having given informed consent) will be maintained in the ISF at the investigational site irrespective of whether they have been treated with investigational drug or not.

3.3.1 Main diagnosis for study entry

This study will be performed in treatment naïve and experienced patients (see inclusion criteria 4 for definitions) with chronic HCV infection of GT1, sub-GT1b virus only.

3.3.2 Inclusion criteria

Chronic HCV infection, diagnosed by positive anti-HCV antibodies and detected HCV RNA at screening in addition to at least one of the following:

a) positive anti-HCV antibodies or detected HCV RNA at least 6 months prior to screening

b) liver biopsy consistent with chronic HCV infection

c) history of elevated ALT levels at least 6 months prior to screening

1. HCV infection, confirmed by genotypic testing at screening, of GT1, sub-GT1b virus.
2. HCV viral load ≥ 1,000 IU/ml at screening
3. Previous treatment status must be one of the following:
   a) Treatment naïve: defined as patients who have never been previously treated with any interferon, with an investigational/approved DAA or any other HCV treatment regimen.
   b) Treatment experienced with prior relapse: defined as HCV RNA level > 25 IU/mL during the post-treatment period in patients who had plasma HCV RNA level undetected at end of all treatment
   c) Treatment experienced interferon intolerant: defined as patients who had to stop a previous PegIFN/RBV regimen before week 12 of treatment due to safety or tolerability issues.
   d) **(Allowed in Cohort A only)** Treatment experienced to an approved PegIFN/RBV regimen with prior partial response: defined as HCV RNA level > 25 IU/mL at end of treatment in patients who had HCV RNA drop by ≥2 log10 from baseline at Week 12.
4. Liver cirrhosis defined as at least one of the following:
   a) Metavir Grade =4 or Ishak Grade ≥5 on liver biopsy

Proprietary confidential information.
© 2012 Boehringer Ingelheim International GmbH or one or more of its affiliated companies. All rights reserved.
This document may not - in full or in part - be passed on, reproduced, published or otherwise used without prior written permission.

Proprietary confidential information © 2015 Boehringer Ingelheim International GmbH or one or more of its affiliated companies

b) liver stiffness of $\geq$13 kPa on fibroscan

5. No evidence of liver cancer in an appropriate imaging study (e.g., ultrasound, CT scan, or magnetic resonance imaging [MRI]) within last 3 months prior to randomisation.
6. No evidence of variceal bleeding or large varices in an upper GI endoscopy, as part of standard of care in patients with liver cirrhosis, performed within 12 months prior to randomization. Note: Patients may be included if they have a history of variceal bleeding or a history of large varices, if properly treated and monitored.
7. Age 18 to 75 years (inclusive)
8. Female patients:
   a) with documented hysterectomy,
   b) who have had both ovaries removed,
   c) with documented tubal ligation,
   d) who are post-menopausal with last menstrual period at least 12 months prior to screening, or
   e) of childbearing potential with a negative serum pregnancy test at screening and Day 1, who, if sexually active, agree to use two non-hormonal methods of birth control from the date of screening until 7 months after the last dose of RBV. Patients must agree not to breast-feed at any time from the date of screening until 7 months after the last dose of RBV. Accepted methods of contraception in the study include diaphragm with spermicidal substance, cervical caps, intrauterine devices and condoms.

      Note: Systemic hormonal contraceptives may not be as effective in women taking BI 207127/FDV combination therapy and are not accepted methods of contraception in the study.

   Male patients:
   a) who are documented to be sterile, or
   b) who are without pregnant female partner(s) and consistently and correctly use a condom while their female partner(s) (if of child-bearing potential) use one of the appropriate medically accepted methods of birth control from the date of screening until 7 months after the last dose of RBV. It is in the responsibility of the male patient to ensure that his partner(s) is not pregnant prior to screening into the study or becomes pregnant during the treatment and the observation phase. Female partners of childbearing potential should perform monthly pregnancy tests from the date of screening until 7 months after the last dose of RBV (tests will be provided by the sponsor).
9. Signed informed consent form prior to trial participation

3.3.3 Exclusion criteria

1. HCV infection of mixed genotype (1/2, 1/3, and 1/4) or mixed sub-GT1a/1b or undefined diagnosed by genotypic testing at screening

Proprietary confidential information.
© 2012 Boehringer Ingelheim International GmbH or one or more of its affiliated companies. All rights reserved.
This document may not - in full or in part - be passed on, reproduced, published or otherwise used without prior written permission.

Proprietary confidential information © 2015 Boehringer Ingelheim International GmbH or one or more of its affiliated companies

2. Liver disease due to causes other than chronic HCV infection which may include but is not limited to hemochromatosis, Wilson's disease, or autoimmune liver diseases.
3. HIV infection
4. Hepatitis B virus infection based on presence of HBs-Ag
5. Confirmed or suspected active malignancy or history of malignancy within the last 5 years prior to screening (with an exception of appropriately treated basal cell carcinoma of the skin or in situ carcinoma of the uterine cervix).
6. History of chronic alcohol abuse or illicit drug use other than cannabis within 12 months prior to randomisation, in the opinion of the investigator.
7. Patient is not willing to comply with the restriction of no alcohol consumption.
8. Patient is not willing to comply with the precautionary measures to prevent photosensitivity (avoid excessive sun exposure and use sun block on a daily basis).
9. A condition that is insufficiently diagnosed, treated or clinically unstable which, in the opinion of investigator, may put the patient at risk because of participation in this study, influence the results of this study, or limit the patient’s ability to participate in this study, including but is not limited to severe chronic obstructive pulmonary disease and uncontrolled psychiatric disease.
10. Total bilirubin > 3 mg/dL with ratio of direct/indirect > 1
11. Serum albumin < 2.8 g/dL
12. Prothrombin time International Normalised Ratio (INR) > 2.3
13. History or active poorly controlled ascites
14. Encephalopathy graded as other than minimal
15. Clinical evidence of unstable cardiovascular disease which may further decompensate due to anemia, including unstable angina, recent myocardial infarction, cardiomyopathy, congestive heart failure, uncontrolled hypertension or significant arrhythmia.
16. Red blood cell (RBC) disorders, including thalassemia major, sickle cell anemia or G6PD deficit. Patients with traits or minor diseases (e.g. sickle cell trait or thalassemia minor) may be enrolled if the disease did not result in anemia according to the investigator’s clinical judgement.
17. Body weight < 40 or > 125 kg
18. Usage of any investigational drugs within 28 days prior to randomisation, or planned usage of an investigational drug during the course of this study
19. Received concomitant immunomodulatory treatment within 28 days prior to screening
20. Received silymarin (milk thistle), glycyrrhizin, Sho-saiko-to (SST) or any medication listed in a restricted medication list provided in ISF within 28 days prior to randomisation, with the exception of parenteral analgesics used during liver biopsy procedure.
21. Known hypersensitivity to any ingredient of the study drugs
22. Hb < 11 g/dL for women and < 12 g/dL for men
23. Absolute neutrophil count < 1,000 cells/mm3
24. Creatinine clearance ≤ 50 ml/min

Proprietary confidential information.
© 2012 Boehringer Ingelheim International GmbH or one or more of its affiliated companies. All rights reserved.
This document may not - in full or in part - be passed on, reproduced, published or otherwise used without prior written permission.

Proprietary confidential information © 2015 Boehringer Ingelheim International GmbH or one or more of its affiliated companies

25. Expected time until liver transplantation less than 6 months
26. MELD score > 20
27. Platelet count < 50,000 cells/mm^3

3.3.4 Removal of patients from therapy or assessments

3.3.4.1 Removal of individual patients

Patients have the right to withdraw from the study at any time without the need to justify the decision. The investigator has the right to remove patients from the study for non-adherence. Furthermore, patients' treatment should be discontinued early due to lack of viral response or other reasons (as described below). The sponsor reserves the right to terminate a patient from the trial for non-adherence. It is understood that an excessive rate of withdrawals can render the study results uninterpretable; therefore unnecessary withdrawal of patients should be avoided. Patients who discontinue their treatment early will be followed throughout the course of the study and undergo all study required procedures as defined in Section 6.2.3.

Patients will discontinue their assigned treatment earlier than planned due to lack of on-treatment viral response:

a) If they have virologic breakthrough defined as:
   1) Increase of ≥1 log10 in plasma HCV RNA from a quantifiable nadir*; or,
   2) HCV RNA ≥25 IU/mL after previous plasma HCV RNA <25 IU/mL, which must be confirmed by a second consecutive plasma HCV RNA measurement as soon as possible and within 2 weeks time. (Note: If HCV RNA <500 IU/mL, the retest should be greater than the first HCV RNA measurement of ≥25 IU/mL before treatment is discontinued.)

b) If they never achieve plasma HCV RNA <25 IU/mL and have plasma HCV RNA ≥25 IU/mL at any time from Week 12 of active treatment onwards (Note: Patients with virologic breakthrough as defined above are not included)

* HCV RNA nadir refers to the lowest HCV RNA measurement while on treatment.

These rules will be used only while patients are on-treatment (i.e. in order to stop treatment due to lack on-treatment viral response). Once patients stop treatment, the retest requirements as described in Section 5.1.1 for SVR endpoints will apply.

Patients may discontinue their assigned treatment earlier than planned due to reasons other than lack of viral response, as listed below and based on the clinical judgement of investigator:

- Development of a toxicity or AE which warrants drug discontinuation.
- Development of any potentially life-threatening toxicity.
- Patient has to take any concomitant drug interfering with the study medications (list provided in ISF: this should be discussed with the BI Clinical Monitor prior to discontinuation).

Proprietary confidential information.
© 2012 Boehringer Ingelheim International GmbH or one or more of its affiliated companies. All rights reserved.
This document may not - in full or in part - be passed on, reproduced, published or otherwise used without prior written permission.

Proprietary confidential information © 2015 Boehringer Ingelheim International GmbH or one or more of its affiliated companies

- Patient is no longer able to participate for other medical reasons (e.g., surgery, pregnancy or medical contraindication to BI 207127, FDV or RBV).
- Concurrent increase in total bilirubin and ALT or aspartate aminotransferase (AST): bilirubin >5x upper limit normal (ULN) with a ratio of direct/indirect bilirubin >1 and ALT or AST >2x baseline with an absolute increase of at least 200 IU/L over the ALT or AST value at baseline.
- Concurrent increase in total bilirubin and prolongation of INR: bilirubin >5x ULN with a ratio of direct/indirect bilirubin >1 and INR >2.3.
- Concurrent increase in ALT or AST and prolongation of INR: ALT or AST >2x baseline with an absolute increase of at least 200 IU/L over the ALT or AST value at baseline, and INR >2.3.

3.3.4.2 Discontinuation of the trial by the sponsor

Boehringer Ingelheim reserves the right to discontinue the trial overall or at a particular trial site at any time for the following reasons:

1. Failure to meet expected enrolment goals overall or at a particular trial site,
2. Emergence of any efficacy/safety information that could significantly affect continuation of the trial,
3. Violation of Good Clinical Practice (GCP), the clinical trial protocol (CTP), or the contract by a trial site or investigator, disturbing the appropriate conduct of the trial.

The investigator / the trial site will be reimbursed for reasonable expenses incurred in case of trial termination (except in case of the third reason).

Proprietary confidential information.
© 2012 Boehringer Ingelheim International GmbH or one or more of its affiliated companies. All rights reserved.
This document may not - in full or in part - be passed on, reproduced, published or otherwise used without prior written permission.

Proprietary confidential information © 2015 Boehringer Ingelheim International GmbH or one or more of its affiliated companies

4. TREATMENTS

4.1 TREATMENTS TO BE ADMINISTERED

4.1.1 Identity of BI investigational products and comparator products

The following tables summarise the information about the investigational, non-investigational medicinal products, and comparator products as used in this trial.

Table 4.1.1: 1 Characteristics of the test products and comparator products

Substance	Pharma-ceutical form	Source	Unit strength	Daily dose	Posology	Route of admin.
**BI 207127**	tablet	Boehringer Ingelheim Pharma GmbH & Co. KG	200 mg	1200 mg; 800 mg	twice daily	oral
**BI 207127 matching placebo**	tablet	Temmler Werke GmbH, Munich, Germany	N/A	NA	twice daily	oral
**FDV**	soft gelatin capsule	Catalent Pharma solutions	120 mg	120 mg	once daily	oral
**FDV matching placebo**	soft gelatin capsule	Catalent Pharma solutions	N/A	NA	once daily	oral
**Ribavirin (Copegus®)**	tablet	F. Hoffmann-LaRoche Ltd	200 mg	1000 (<75 kg body weight); 1200mg (≥ 75 kg body weight)	twice daily	oral
**Ribavirin (Copegus®)**	tablet over-encapsulated	F. Hoffmann-LaRoche Ltd; encapsulation by Almac Clinical Services	200 mg	1000 (<75 kg body weight); 1200mg (≥ 75 kg body weight)	twice daily	oral
**Ribavirin matching placebo**	tablet over-encapsulated	Almac Clinical Services	N/A	NA	twice daily	oral

4.1.2 Method of assigning patients to treatment groups

After assessment of all entry criteria, the investigator or designee at the site will follow the process outlined in the ISF to assign the patient to a treatment group at visit 2. This will involve the use of an IRT system which will assign eligible patients to a treatment arm according to current screening stage of the trial (cf. Figure 3.1: 2). Treatment group assignment will be recorded on the eCRF and source document. For statistical features of the treatment allocation process refer to Section 7.5.

IRT will assign the first 48 patients randomised in Cohort B to participate the PK sub-study (cf. Section 5.5 and Appendix 10.1).

Proprietary confidential information.
© 2012 Boehringer Ingelheim International GmbH or one or more of its affiliated companies. All rights reserved.
This document may not - in full or in part - be passed on, reproduced, published or otherwise used without prior written permission.

Proprietary confidential information © 2015 Boehringer Ingelheim International GmbH or one or more of its affiliated companies

4.1.3 Selection of doses in the trial

The doses selected to be evaluated in this trial are BI 201727 400 mg b.i.d. or BI 201727 600 mg b.i.d. along with FDV 120 mg q.d. and weight-based RBV.

In SOUND-C2, the doses of BI 207127 600 mg b.i.d. in combination with FDV 120 mg q.d. and RBV demonstrated a favourable safety profile with a low rate of discontinuations due to AEs, and the absence of moderate or severe skin events or discontinuations due to skin events (rash or photosensitivity).

Safety and efficacy results from SOUND-C2 in patients with compensated liver cirrhosis were favourable (cf. Section 2.3). However, a 2-fold increase in exposure of both FDV and BI 207127 in cirrhotic patients compared to non-cirrhotic patients was observed. As such, it is expected that FDV and BI 207127 exposure in CPB patients will be at least 2-fold higher than that of non-cirrhotic patients. Therefore, a 400 mg b.i.d. dose of BI 207127 in combination with 120 mg FDV q.d. and RBV in CPB cirrhotic patients is expected to achieve similar or higher exposure than 600 mg b.i.d. dose group in non-cirrhotic patients. If this is not the case and exposure is lower, Arm 3 will be conducted to test the 600 mg b.i.d. dose in CPB patients before the start of Cohort B. The dose for Cohort B will be selected and released by the internal data review committee.

PK / pharmacodynamic (PD) analyses performed in SOUND-C1 and 2 indicate that a PK exposure higher than the one achieved with BI 207127 600 mg b.i.d. does not provide an efficacy benefit in target patient population but rather negatively affects the safety profile. In particular, PK trough levels achieved with the 600 mg b.i.d. dose of BI 207127 were already beyond the exposure-response threshold for GT1b virus. BI 207127 doses higher than 600 mg b.i.d. did not increase viral response but impacted negatively on the safety profile (more severe skin and GI events).

No BI 207127 induction dose will be used (cf. Section 2.3).

Based on the current understanding that cirrhotic patients require longer treatment durations and the lack of substantial clinical data in cirrhotic patients treated for 16 weeks in phase 2, patients in this trial are planned to receive treatment for 24 weeks only.

4.1.4 Drug assignment and administration of doses for each patient

For Cohort A (Arm 2 and Arm 3 only), once an investigator establishes that a CPB patient meets all entry criteria, the investigator will send electronically to the TCM a written request for assignment of the treatment visit date. The investigator will be required to receive written approval from the sponsor before confirming the appointment for the treatment visit with the patient (cf. ISF for request/approval form to be used).

The investigator will make a randomisation call to IRT to randomise/assign an eligible patient. Dispensing of study drugs will begin at Visit 2.

Proprietary confidential information.
© 2012 Boehringer Ingelheim International GmbH or one or more of its affiliated companies. All rights reserved.
This document may not - in full or in part - be passed on, reproduced, published or otherwise used without prior written permission.

Proprietary confidential information © 2015 Boehringer Ingelheim International GmbH or one or more of its affiliated companies

IRT will assign the medication number(s). The assigned medication number(s) must be entered in the eCRF, and the corresponding medication unit(s) must be given to the patient. Each medication unit will have a unique medication number and more than one medication unit may be assigned by IRT dependent on the visit schedule. The visits at which medication is dispensed to patients are specified in the Flow Chart. For details please refer to the ISF.

FDV (or matching placebo) loading dose on Day 1 will include an additional 120 mg capsule for a total of 240 mg for the first active dose. FDV (or matching placebo) should be taken q.d. in the morning together with the morning doses of BI 207127 and RBV (or matching placebo), and together with food. The evening doses of BI 207127 and RBV (or matching placebo) should be taken together with food 12 hours after the morning dose.

Patients in all groups are planned to receive study drugs (active or placebo) for a total of 24 weeks. Early treatment discontinuation rules for lack of viral response or other reasons are defined for all groups (cf. Section 3.3.4.1).

If either of FDV or BI 207127 needs to be discontinued early all three trial medications need to be discontinued. Dose reductions of FDV or BI 207127 are not allowed in this trial. The dose of RBV may be reduced or RBV may be temporarily discontinued due to anaemia (cf. Section 4.2.1) while still continuing FDV and BI 207127 treatment. However, RBV should be reintroduced even at a reduced dose as soon as clinically possible.

Treatment interruptions must be avoided as much as possible. If the combination therapy is interrupted due to an unavoidable reason, it should restart no later than 3 days after the treatment was interrupted. Treatment interruptions for more than 3 days are not allowed and in these cases, treatment has to be stopped permanently and an EOT visit should be performed (cf. Section 6.2.3).

4.1.5 Blinding and procedures for unblinding

4.1.5.1 Blinding

Cohort A

Treatment Arm 1, Arm 2, and Arm 3 will be open-label.

Cohort B

Study medications will be administered double blinded in treatment Arm 4 and Arm 5.

It is recognised that this blinding will be challenging because of multiple laboratory test changes, specifically viral load, bilirubin, liver enzymes and hematologic changes that are related to the administration of active triple therapy *vs.* no such changes in patients with placebo. However, it is important to maintain the blinding through the blinding of specific laboratory assessments (cf. Table 4.1.5.1: 1) to minimize assessment bias as well as to ensure compliance with study procedures, retain the patients in the study, and control for AEs inherent in the target population.

Proprietary confidential information.
© 2012 Boehringer Ingelheim International GmbH or one or more of its affiliated companies. All rights reserved.
This document may not - in full or in part - be passed on, reproduced, published or otherwise used without prior written permission.

Proprietary confidential information © 2015 Boehringer Ingelheim International GmbH or one or more of its affiliated companies

In order to mitigate the risk of indirect unblinding and to maintain the integrity of the blinded assessments, Arm 4 and Arm 5 results of plasma HCV RNA levels and a series of safety laboratory tests after start of the treatment will be blinded to investigators, patients and sponsor for up to Week 24 visit (non-inclusive, i.e., Week 24 results will be reported to investigators) (cf. Table 4.1.5.1: 1). These safety laboratory results will be made available to the investigator if any of the criteria are met as described in Table 4.1.5.1: 1.

Central laboratory service will disclose the results of safety laboratory parameters when the unblinding criteria for those laboratory tests are met. If any of ALT or AST meets the criteria for unblinding, results for both laboratory tests will be disclosed from that time-point forward. If Hb meets the criteria for unblinding results for RBC count, Hb, hematocrit will be disclosed from that time point forward. If for the patient care management the investigator needs to know the blinded laboratory results even prior meeting the criteria established in Table 4.1.5.1: 1, the investigator will be able to request these laboratory tests (except HCV RNA) from central laboratory service after consultation with BI Clinical Monitor.

Central laboratory service will inform the investigator whether the patient meets lack of on treatment viral response criteria based on HCV RNA, without disclosing the actual results of HCV RNA. If any of these stopping rules are met, the investigator will receive a notification for repeat testing or treatment discontinuation.

Note that treatment code will be only broken upon investigator's request as per procedures described in Section 4.1.5.2.

Table 4.1.5.1: 1 Blinded laboratory tests for Arm 4 and Arm 5 from after start of treatment to up to Week 24 visit (non-inclusive) and criteria for unblinding

**Blinded laboratory parameters**	**Criteria for unblinding the laboratory parameters**
HCV RNA	All stopping rules (cf. Section 3.3.4.1)
ALT	> 2x of baseline with an absolute increase of at least 100 IU/L compared to baseline
AST	> 2x of baseline with an absolute increase of at least 100 IU/L compared to baseline
Bilirubin (total, direct and indirect)	Bilirubin > 3 mg/dL with a ratio of direct/indirect bilirubin >1
RBC laboratory assessment (RBC count, Hb, hematocrit)	Hb <10.5 g/dL; or, a Hb drop ≥ 2 g/dL during any 4 week period

For Cohort B, the sponsor, patient, and investigator will be unblinded to all laboratory values and treatment (active or placebo) for each patient at their EOT visit.

Proprietary confidential information.
© 2012 Boehringer Ingelheim International GmbH or one or more of its affiliated companies. All rights reserved.
This document may not - in full or in part - be passed on, reproduced, published or otherwise used without prior written permission.

Proprietary confidential information © 2015 Boehringer Ingelheim International GmbH or one or more of its affiliated companies

According to BI's SOPs, suspected unexpected serious adverse reactions (SUSARs) will be unblinded for reporting to competent authorities. In such cases, access to the code will only be permitted by authorized drug safety representatives via IRT.

4.1.5.2 Procedures for emergency unblinding

An emergency code break will be available to the investigator/pharmacist/investigational drug storage manager via IRT. Details are included in the ISF. This code break may only be used in emergency situations when the identity of the trial drug must be known to the investigator in order to provide appropriate medical treatment or if required to assure safety of trial participants.

If the code break for a patient is used, the sponsor must be informed immediately. The reason for using the code break, the date when the code was broken and initials of person who broke the code must be documented on the appropriate electronic case report form (eCRF) page.

4.1.6 Packaging, labelling, and re-supply

BI 207127 (or matching placebo) are packaged in a carton box containing 2 bottles of 90 tablets each, for a total of 180 tables per medication unit.

FDV (also known as BI 201335, or matching placebo) are packaged in a bottle of 60 soft gelatine capsules per medication unit.

RBV (Copegus®) are packaged in a carton box containing 3 bottles of 60 tablets each, for a total of 180 tablets per medication unit.

RBV (Copegus®) over-encapsulated (or matching placebo) are packaged in a carton box containing 3 bottles of 60 tablets each, for a total of 180 tablets per medication unit.

Supply and re-supply will be managed by IRT.

For details of packaging and the description of the label, refer to the ISF.

4.1.7 Storage conditions

Drug supplies will be kept in their original packaging under the recommended storage conditions indicated on the label. Storage temperature should be monitored and documented.

The trial medication must be stored securely, e.g. in a locked cupboard or at a pharmacy. It may only be dispensed to trial patients according to the protocol by authorised personnel as documented in the form "Investigator's Trial Staff".

All unused medication must be returned to the sponsor. Receipt, usage and return must be documented on the respective forms. Account must be given for any discrepancies.

Proprietary confidential information.
© 2012 Boehringer Ingelheim International GmbH or one or more of its affiliated companies. All rights reserved.
This document may not - in full or in part - be passed on, reproduced, published or otherwise used without prior written permission.

Proprietary confidential information © 2015 Boehringer Ingelheim International GmbH or one or more of its affiliated companies

4.1.8 Drug accountability

Drug supplies, which will be provided by the sponsor or a CRO appointed by the sponsor, must be kept in a secure, limited access storage area under the storage conditions defined by the sponsor. Where necessary, a temperature log must be maintained to make certain that the drug supplies are stored at the correct temperature.

The investigator/pharmacist/investigational drug storage manager will receive the investigational drugs delivered by the sponsor when the following requirements are fulfilled:

- approval of the study protocol by the Institutional Review Board (IRB) / Independent Ethics Committee (IEC),
- availability of a signed and dated clinical trial contract between the sponsor and the Head of Trial Centre,
- approval/notification of the regulatory authority, e.g. competent authority (CA),
- availability of the Principal Investigator’s (PI’s) CV,
- availability of a signed and dated CTP or immediately imminent signing of the CTP (in exceptional cases, medication could already be sent to the site, before its activation via IRT),
- availability of the proof of a medical licence for the PI, if applicable
- availability of the Form 1572 (for USA).

The investigator/pharmacist/investigational drug storage manager must maintain records of the product’s delivery to the trial site, the inventory at the site, the use by each patient, and the return to the sponsor or alternative disposition of unused product(s).

These records will include dates, quantities, batch/serial numbers, expiry (‘use by’) dates, and the unique code numbers assigned to the investigational product(s) and trial patients. The investigator/pharmacist/investigational drug storage manager will maintain records that document adequately that the patients were provided the doses specified by the CTP and reconcile all investigational product(s) received from the sponsor. At the time of return to the sponsor or appointed CRO, the investigator/pharmacist/investigational drug storage manager must verify that all unused or partially used drug supplies have been returned by the clinical trial patient and that no remaining supplies are in the investigator’s possession.

4.2 CONCOMITANT THERAPY AND RESTRICTIONS, AND RESCUE TREATMENT

4.2.1 Rescue medication, emergency procedures, and additional treatments

If either of FDV or BI 207127 needs to be discontinued early, all three trial medications need to be discontinued (for further details cf. Section 4.1.4). If RBV-related severe adverse reactions or laboratory abnormalities develop during combination therapy, the dose of RBV may be reduced or RBV may be temporarily discontinued until the adverse reactions abate while still continuing FDV or BI 207127 treatment.

Proprietary confidential information.
© 2012 Boehringer Ingelheim International GmbH or one or more of its affiliated companies. All rights reserved.
This document may not - in full or in part - be passed on, reproduced, published or otherwise used without prior written permission.

Proprietary confidential information © 2015 Boehringer Ingelheim International GmbH or one or more of its affiliated companies

All dose adjustments of RBV, discontinuations and rescue medications must be documented in the patient source documentation and corresponding eCRF.

Management of drug-related skin events:

In case of drug-related skin events, the investigator should follow the dermatologic management plan, as specified in Appendix 10.2.

Management of gastrointestinal (GI) events:

GI AEs such as nausea, vomiting or diarrhoea have been reported during treatment with BI 207127 and FDV. The highest onset rate of these AEs was during the first week of treatment, particularly Day 1 and 2, followed by a significant decrease over time.

The following general measures are strongly recommended in order to prevent and manage these AEs:

- BI 207127 doses should be taken along with food avoiding intake in fasting conditions. Patients should be instructed to take their medications with food, preferably during breakfast and dinner.
- Early symptomatic treatment is recommended particularly during the first days of treatment.
- Antiemetic treatments such as ondansetron 8 mg tablets starting with one tablet b.i.d. or metoclopramide 10 mg tablets (orally dissolved) one tablet b.i.d. or q8h are recommended for nausea and vomiting. These agents may be taken approximately 30 minutes before BI 207127 intake if needed, and continued depending upon response. Re-taking study medications after vomiting is not recommended; patients should follow their regular schedule.
- Antimotility agents such as loperamide 2 mg caplets (or capsules) are recommended for non-infectious diarrhea; starting with 4 mg dose (2 caplets) and continue with 2 mg after each subsequent loose stool but no more than 4 caplets in 24 hours.

Management of anaemia

For RBV dose modification guidance for management of anaemia cf. to Table 4.2.1: 1. Note that even though Hb values are blinded for Cohort B, investigator will be receiving laboratory reports if a patients experience a Hb <10.5 g/dL or, a Hb drop ≥ 2 g/dL during any 4 week period (cf. Table 4.1.5.1: 1).

Proprietary confidential information.
© 2012 Boehringer Ingelheim International GmbH or one or more of its affiliated companies. All rights reserved.
This document may not - in full or in part - be passed on, reproduced, published or otherwise used without prior written permission.

Proprietary confidential information © 2015 Boehringer Ingelheim International GmbH or one or more of its affiliated companies

Table 4.2.1: 1 Ribavirin hemoglobin dosage modification guidelines

Laboratory Values	Reduce only Ribavirin dose to 600mg/day[1], if:	Discontinue Ribavirin[1], if:
Hb in patients with no cardiac disease	<10 g/dL	<8.5 g/dL
Hb in patients with history of stable cardiac disease	≥2 g/dL decrease in Hb during any 4-week period treatment	<12 g/dL despite 4 weeks at reduced dose

[1] If the abnormality is reversed, Ribavirin should be restarted at 600 mg daily, and further increased to 800 mg daily at the discretion of the treating physician. However, a return to higher doses is not recommended.

Of note, other ribavirin dosage modification guidelines due to anaemia may apply according to local approved Copegus® label.

The use of eyrthropoiesis stimulating agents (ESA), is permitted at the discretion of the investigator. If ESA are being prescribed, the following guidelines have to be followed:

- ESA should not be initiated until haemoglobin levels falls below 10g/dL
- Iron studies should be obtained prior to and during ESA treatment
- Iron supplementation should be initiated for deficient patients and to maintain transferring saturation at levels supporting erythropoiesis
- Once an ESA is initiated, haemoglobin levels and blood pressure must be monitored weekly until stabilisation of haemoglobin
- Treatment should target a haemoglobin level sufficient to avoid transfusion
- ESA dose should be titrated to treatment response
- ESA dose should be reduced if the haemoglobin rises by more than 1 g/dL in a 2-week period
- ESA dose should not exceed those recommended for currently approved indications
- ESA should not be used in patients at increased risk for thromboembolic events, cardiovascular events, including those with inadequately controlled hypertension, and patients with diagnosed malignancies
- Patients must be informed about the risks associated with ESA use, as described in the patient information
- Dose and duration of treatment must be documented in the eCRF

Treatment option for patients that complete Arm 4

Eligible patients who accept the offer to receive active treatment (corresponding to Arm 5) will follow the visit order and procedures specified in Table 4.2.1: 2 as well as the treatment discontinuation criteria as defined in Section 3.3. Refer to ISF for further details.

Proprietary confidential information.
© 2012 Boehringer Ingelheim International GmbH or one or more of its affiliated companies. All rights reserved.
This document may not - in full or in part - be passed on, reproduced, published or otherwise used without prior written permission.

Proprietary confidential information © 2015 Boehringer Ingelheim International GmbH or one or more of its affiliated companies

Table 4.2.1: 2 Arm 4 treatment option schedule

	Treatment Period						A-FU[2]	
**Visit**	A1	A2	A3	A4	A5	A-EOT	A-FU1	A-FU2
**Week**	0	4	8	12	16	24	A-EOT +12	A-EOT +24
Rescue medication eligibility assessment	X							
Treatment assignment via IRT	X							
Pregnancy screening	X	X	X	X	X	X	X	X
Physical examination[5]	X^T	X^T	X^T	X^T	X^T	X^T	X^T	X^T
HCV RNA	X	X	X	X	X	X	X	X
Virology samples	X	X	X	X	X	X	X	X
Laboratory tests[1]	X	X	X	X	X	X	X	X
SAEs[4]	X	X	X	X	X	X	X	X
Dispense rescue medications	X[3]	X	X	X	X			

1 Selected laboratory tests will be performed (cf. Laboratory Manual)
2 FU (follow-up) visits are counted from EOT (end of treatment).
3 Loading dose of FDV is needed.
4 All AEs with onset date up to 4 weeks after A-EOT will be recorded. Only related to FDV or related to BI 207127 or fatal SAEs will need to be reported through SAE form if onset date is 4 weeks after A-EOT.
5 "X^T" is a targeted physical examination including measurements of vital signs, and evaluation of organ systems particularly associated with AE(s) symptoms.

4.2.2 Restrictions

4.2.2.1 Restrictions regarding concomitant treatment

Concomitant use of medications should be guided by the '*restricted*' and '*use with caution*' drug lists provided in the ISF.

Concomitant immunomodulatory treatment, including the chronic administration of systemic corticosteroids, is discouraged and if clinically possible should be postponed until EOT. Note that topical, nasal, or pulmonary steroids may be used (if not listed as restricted in ISF).

Systemic antiviral treatment will not be allowed during treatment period this trial, with the exception of oral antiviral such as acyclovir, famiclovir or valacyclovir for mild, localised recurrent herpes simplex infection, or oseltamivir or zanamivir for Influenza A.

The use of silibinin or other medications extracted from the milk thistle is not allowed during the treatment period of this trial.

The co-administration of drugs which may cause photosensitivity reactions should be avoided throughout the treatment duration and up until 28 days after all treatment discontinuation, unless the benefit outweighs the risk based on the investigators' clinical judgment. The restriction of drugs with photosensitivity potential includes and is not limited to tetracyclines (e.g. tetracycline, doxycycline), fluoroquinolones (e.g. ciprofloxacin, ofloxacine), sulfonamides, sulfonylureas (e.g. glipizide), psoralens, oral retinoids (e.g. isotretinoin, acitretin), phenothiazines and amiodarone.

Proprietary confidential information.
© 2012 Boehringer Ingelheim International GmbH or one or more of its affiliated companies. All rights reserved.
This document may not - in full or in part - be passed on, reproduced, published or otherwise used without prior written permission.

Proprietary confidential information © 2015 Boehringer Ingelheim International GmbH or one or more of its affiliated companies

All concomitant and/or rescue therapies will be recorded on the appropriate pages of the eCRFs.

4.2.2.2 Restrictions on diet and life style

Patients must be willing to protect any uncovered skin area (including hands, face and lips) from sun- or UV-light exposure using sun-blocker cream with an SPF ≥50 providing UV-A and -B protection during treatment period on a daily basis. Lip balm with the highest SPF locally available should be used. Patients should avoid unnecessary or prolonged exposure to sunlight and wear protective clothing, sunglasses and hats in addition to sun-block. It is recommended to avoid direct sun exposure as much as possible. Tanning booths must be avoided during treatment period.

No alcohol consumption during screening, treatment, and follow-up periods of the trial.

4.3 TREATMENT COMPLIANCE

Study medication will be dispensed to the patient at the study site by responsible site personnel. Details regarding dispensing of the study medication to each participating patient, including patient identification, the amount of study drug dispensed, the date the drug was dispensed, and the numbers of capsules/tablets/vials returned to the site will be recorded in the drug dispensing log. All dispensed study drug should be recorded in the drug-dispensing log in the ISF.

Patients must return to the site drug packaging including unused or empty medication packages at each visit. Study medication usage and return must be documented on the respective form and account must be given for any discrepancies.

Treatment compliance for all three drugs will be strictly monitored and assessed at each relevant visit through pill-count by Investigator (or designated site personnel) according to worksheets provided in ISF. For more information regarding FDV pill-count procedure, please cf. ISF. Compliance worksheets must be kept as a source documents. BI Clinical Monitor should be contacted for cases of suspected non-compliance.

Compliance will be calculated according to the formula:

- Compliance (%) = Number of medications actually taken since last count / Number of medications which should have been taken in the same period ×100%

Planned interruptions and dose reductions in treatment (applicable for RBV) in accordance with the protocol and prescribed by the investigator should be taken into account when calculating compliance.

If the treatment compliance cannot be assessed in a specific visit (e.g., bottle is lost or forgotten), the eCRF compliance page at that visit should be left blank.

Compliance to the BI 207127, FDV, RBV and matching placebos should be between 80% and 120%. Patients who are non-compliant according to this definition will be treated as

Proprietary confidential information.
© 2012 Boehringer Ingelheim International GmbH or one or more of its affiliated companies. All rights reserved.
This document may not - in full or in part - be passed on, reproduced, published or otherwise used without prior written permission.

Proprietary confidential information © 2015 Boehringer Ingelheim International GmbH or one or more of its affiliated companies

protocol violations. The identification, handling and inclusion/exclusion of these and other protocol violations will be discussed in the trial statistical analysis plan (TSAP).

Patients who are non-compliant (e.g., have low treatment compliance, do not attend trial visits or violate the restrictions) may be withdrawn from the trial and the eCRF will be completed accordingly (for further procedures cf. Section 3.3.4), following discussion with BI Clinical Monitor.

Patients should take their medication regularly at the same time every day (BI 207127 and RBV every 12-hour and FDV every 24-hour). Excursions ± 1 hour outside from the regular times of intake should be avoided since this may lead to viral resistance and treatment failure.

If a patient misses a scheduled BI 207127 dose, and discovers this error:

- Less than 6 hours after the scheduled dose time, the patient must: take the missed dose; take all remaining doses at scheduled time; and, report the error at the next study visit.
- 6 hours or more after the scheduled dose time, the patient must: take the next scheduled dose at the next scheduled time; not take the missed dose; and, report the error at the next study visit.

If a patient misses a scheduled FDV dose, and discovers this error:

- Less than 12 hours after the scheduled dose time, the patient must: take the missed dose; take all remaining doses at scheduled time; and, report the error at the next study visit.
- 12 hours or more after the scheduled dose time, the patient must: take the next scheduled dose at the next scheduled time; not take the missed dose; and, report the error at the next study visit.

If a patient misses a scheduled RBV dose, and discovers this error:

- Less than 6 hours after the scheduled dose time, the patient must: take the missed dose; take all remaining doses at scheduled time; and, report the error at the next study visit.
- 6 hours or more after the scheduled dose time, the patient must: take the next scheduled dose at the next scheduled time; not take the missed dose; and, report the error at the next study visit.

Proprietary confidential information.
© 2012 Boehringer Ingelheim International GmbH or one or more of its affiliated companies. All rights reserved.
This document may not - in full or in part - be passed on, reproduced, published or otherwise used without prior written permission.

Proprietary confidential information © 2015 Boehringer Ingelheim International GmbH or one or more of its affiliated companies

5. VARIABLES AND THEIR ASSESSMENT

5.1 EFFICACY - CLINICAL PHARMACOLOGY

5.1.1 Endpoints of efficacy

Primary efficacy endpoint:

- SVR at Week 12 post-treatment (SVR12): Plasma HCV RNA level <25 IU/mL at 12 weeks after EOT

Secondary efficacy endpoints:

- SVR4: Plasma HCV RNA level <25 IU/mL at 4 weeks after EOT
- SVR24: Plasma HCV RNA level <25 IU/mL at 24 weeks after EOT

Note: Patients with *HCV RNA level <25 IU/mL, detected* during the FU period should have a HCV RNA retest as soon as possible (within two weeks). Further retests are needed until *HCV RNA ≥25 IU/mL* (relapse) or *HCV RNA <25 IU/mL, undetected* is obtained.

Other efficacy endpoints:

- Virological Response (VR) at Week 4:

  -Plasma HCV RNA undetected at Week 4 ($W4U_{TND}$)

  -Plasma HCV RNA level <25 IU/mL at Week 4 (W4U)

- Change in Child-Turcotte-Pugh or MELD score by ≥1 point 12 weeks after EOT
- Plasma HCV undetected at Week 12 ($W12U_{TND}$)
- End of Treatment Response: Plasma HCV RNA level undetected at end of all therapy (ETR_{TND})
- Time to achieving HCV RNA undetected
- Time on treatment with HCV RNA undetected
- Virologic breakthrough (as defined in Section 3.3.4.1)
- Lack of on-treatment viral response (as defined in Section 3.3.4.1)
- Time to virologic breakthrough (from first HCV RNA undetected)
- Relapse: HCV RNA level > 25 IU/mL during the post-treatment period in patients who had ETR_{TND} at the end of the planned treatment
- Biochemical response

Proprietary confidential information.
© 2012 Boehringer Ingelheim International GmbH or one or more of its affiliated companies. All rights reserved.
This document may not - in full or in part - be passed on, reproduced, published or otherwise used without prior written permission.

Proprietary confidential information © 2015 Boehringer Ingelheim International GmbH or one or more of its affiliated companies

-ALT and aspartame aminotransferase (AST) in normal range at EOT

- -ALT and AST in normal range post-treatment Events of liver disease progression (cf. Section 5.3.2.2)

5.1.2 Assessment of efficacy

A plasma sample will be obtained from all patients at each visit for the measurement of HCV RNA. The plasma HCV RNA level will be measured using the quantitative Roche COBAS® Taqman HCV/HPS assay (Version 2), a test that utilises real-time reverse transcription polymerase chain reaction (PCR) technology. The assay has a limit of detection between 10 and 20 IU/mL and a linear range of 25 IU/mL to 3.91×10^8 IU/mL.

The results below the linear range, HCV RNA levels <25 IU/mL, will be reported as being *undetected* or *detected*. For assessment of HCV RNA related endpoints, HCV RNA level <25 IU/mL is regardless whether HCV RNA is detected or undetected. HCV RNA undetected will be reported as *<25 IU/mL, undetected*.

Detailed instructions for obtaining, handling, and shipping HCV RNA measurement samples are provided in the ISF. Samples will be processed by a central laboratory service.

5.2 SAFETY

5.2.1 Endpoints of safety

The following safety endpoints will be assessed:

- AEs, including Division of Acquired Immunodeficiency Syndrome (DAIDS) grades
- AEs leading to treatment discontinuation of all trial medications
- SAEs
- Laboratory test abnormalities by DAIDS grades
- Change in laboratory test values over time

5.2.2 Assessment of adverse events

5.2.2.1 Definitions of adverse events

Adverse event

An AE is defined as any untoward medical occurrence, including an exacerbation of a pre-existing condition, in a patient in a clinical investigation who received a pharmaceutical product. The event does not necessarily have to have a causal relationship with this treatment.

Proprietary confidential information.
© 2012 Boehringer Ingelheim International GmbH or one or more of its affiliated companies. All rights reserved.
This document may not - in full or in part - be passed on, reproduced, published or otherwise used without prior written permission.

Proprietary confidential information © 2015 Boehringer Ingelheim International GmbH or one or more of its affiliated companies

Serious adverse event

An SAE is defined as any AE which results in death, is immediately life-threatening, results in persistent or significant disability / incapacity, requires or prolongs patient hospitalisation, is a congenital anomaly / birth defect, or is to be deemed serious for any other reason if it is an important medical event when based upon appropriate medical judgement which may jeopardise the patient and may require medical or surgical intervention to prevent one of the other outcomes listed in the above definitions.

Intensity of adverse event

All AEs with the exception of photosensitivity reaction will be graded according to DAIDS grading scale (see DAIDS grading scale in ISF (R10-1332)). Photosensitivity reactions will be graded according to a scale provided in the Dermatology Management Plan in Appendix 10.2.

The intensity of the AE should be judged based on the DAIDS Grading Scale:

- Mild: Grade 1
- Moderate: Grade 2
- Severe: Grade 3
- Potentially Life Threatening: Grade 4

Causal relationship of adverse event

Medical judgment should be used to determine the relationship, considering all relevant factors, including pattern of reaction, temporal relationship, de-challenge or re-challenge, confounding factors such as concomitant medication, concomitant diseases and relevant history. Assessment of causal relationship should be recorded in eCRFs.

Yes: There is a reasonable causal relationship between the investigational product administered and the AE.
No: There is no reasonable causal relationship between the investigational product administered and the AE.

If a SAE is reported from a still blinded trial, the causal relationship must be provided by the investigator for all potential trial drugs, i.e. the BI trial drug and for all other trial drugs (i.e. any active comparator or placebo according to the trial design).

Worsening of the underlying disease or other pre-existing conditions

Worsening of the underlying disease or of other pre-existing conditions will be recorded as an (S)AE in the eCRF.

Changes in vital signs, ECG, physical examination, and laboratory test results

Changes in vital signs, ECG, physical examination and laboratory test results will be recorded as an (S)AE in the (e)CRF , if they are judged clinically relevant by the investigator.

Proprietary confidential information.
© 2012 Boehringer Ingelheim International GmbH or one or more of its affiliated companies. All rights reserved.
This document may not - in full or in part - be passed on, reproduced, published or otherwise used without prior written permission.

Proprietary confidential information © 2015 Boehringer Ingelheim International GmbH or one or more of its affiliated companies

As described in Section 4.1.5.1 selected laboratory tests results in Arm 4 and 5 will be blinded to investigator, patients and sponsor. Therefore, it is understood that by design investigator will not be able to report laboratory related AEs, unless they are in the range that require unbinding of the investigator to the results (cf. Table 4.1.5.1: 1). The blinding ranges were set to exclude values that may be clinically relevant to avoid an effect on the AE reporting rates. In the Clinical Trial Report (CTR), all laboratory tests will be analyzed and presented to demonstrate any potential effect of laboratory test blinding on AE reporting rates.

Protocol-specified significant events

The following are considered as protocol-specified significant events:

- Skin rash cases of Grade ≥2 severity or photosensitivity reactions of moderate or greater severity.

Protocol-specified significant events are to be reported in an expedited manner similar to SAEs, even if they do not meet any of the seriousness criteria. For details please see Section 5.2.2.2.

5.2.2.2 Adverse event and serious adverse event reporting

All AEs, serious and non-serious, with onset date from signing the informed consent until 28 days after all treatment discontinuation (EOT), defined as the FU1 visit, and all related to study medication or fatal SAEs with onset date after FU1 visit will be collected, documented and reported to the sponsor by the investigator on the appropriate eCRFs/SAE reporting forms. Reporting will be done according to the specific definitions and instructions detailed in the 'Adverse Event Reporting' section of the ISF.

For each AE, the investigator will provide the onset date, end date, intensity, treatment required, outcome, seriousness, and action taken with the investigational drug. The investigator will determine the relationship of the investigational drug to all AEs as defined in Section 5.2.2.1. For details refer to the subsections of this Section.

If not stipulated differently in the ISF, the investigator must report the following events 1) if using paper process SAE form via telephone/fax or 2) if available for the trial, using the electronic submission process (RDC) immediately (within 24 hours or the next business day whichever is shorter) to the sponsor: SAEs and non-serious AEs occurring at the same time as an SAE and/or which are medically related to the SAE(s), and protocol-specified significant events.

BI has set up a list of AEs which are defined to be always serious. In order to support the investigator with the identification of these “always serious adverse events”, if a non serious AE is identified to be serious per BI definition, a query will be raised. The investigator must verify the description and seriousness of the event. If the event description is correct, the item “serious” needs to be ticked and an SAE has to be reported in expedited fashion

Proprietary confidential information.
© 2012 Boehringer Ingelheim International GmbH or one or more of its affiliated companies. All rights reserved.
This document may not - in full or in part - be passed on, reproduced, published or otherwise used without prior written permission.

Proprietary confidential information © 2015 Boehringer Ingelheim International GmbH or one or more of its affiliated companies

following the same procedure as above. The list of these AEs can be found via the RDC-system.

The SAE form is to be forwarded to the defined unique entry point identified for the BI OPU (country-specific contact details will be provided in the ISF) or by using the electronic submission process. This immediate report is required irrespective of whether the investigational product has been administered or not and irrespective of causal relationship. It also applies if new information to existing SAEs or protocol-specified significant events becomes available.

Reporting AEs occurring up to 28 days after all treatment discontinuation (FU1 visit):

The investigator has the responsibility to report all AEs occurring up to 28 days after all treatment discontinuation (FU1 visit), regardless whether they are serious, non-serious, related or not-related to the trial medication. Any AEs persisting after FU1 visit must be followed until they have resolved, or as deemed reasonable by the investigator in consultation with BI Clinical Monitor. These AEs will be considered “on-treatment”, except if the onset date was after the start of the active treatment option (for Arm 4 patients only).

Reporting AEs occurring later than 28 days after all treatment discontinuation (FU1 visit):

AEs that occur after FU1 visit will be reported only if serious and deemed related to the trial medication or study design or if fatal. These events will be attributed to the “post-treatment period.”

After the study completion (End of Observation (EOO) visit), the investigator has the responsibility to report only AEs that are serious and deemed related to the trial medication or study design, if the investigator becomes aware of them, but no active FU is necessary after EOO visit. These events will be attributed to the “post-study period.”

Pregnancy

In rare cases, pregnancy might occur in clinical trials. Once a female subject has been enrolled into the clinical trial, after having taken study medication, the investigator must report immediately any drug exposure during pregnancy to the sponsor. Drug exposure during pregnancy has to be reported immediately (within 24 hours or next business day whichever is shorter) to the defined unique entry point for SAE forms of the respective BI OPU (country-specific contact details will be provided in the ISF). The outcome of the pregnancy associated with the drug exposure during pregnancy must be followed up. In the absence of an (S)AE, only the Pregnancy Monitoring Form for Clinical Trials and not the SAE form is to be completed. The ISF will contain the Pregnancy Monitoring Form for Clinical Trials (Part A and Part B).

Reporting protocol-defined significant AEs

Skin AEs of moderate or greater severity deemed related to the trial medications should be reported as protocol-defined significant events. If not stipulated differently in the ISF, protocol-defined significant AEs (cf. Section 5.2.2.1) should be reported on the

Proprietary confidential information.
© 2012 Boehringer Ingelheim International GmbH or one or more of its affiliated companies. All rights reserved.
This document may not - in full or in part - be passed on, reproduced, published or otherwise used without prior written permission.

Proprietary confidential information © 2015 Boehringer Ingelheim International GmbH or one or more of its affiliated companies

SAE/Significant AE form immediately (within 24 hours or the next business day whichever is shorter even if not serious) as well as on the AE eCRF page and the skin eCRF page. Please note, the event should NOT be reported as serious on the SAE form unless it meets the regulatory definitions of a SAE defined in the "Adverse Event Reporting" section of the ISF. It is recommended that all patients with protocol-defined significant AEs should see be referred to a local dermatologist for diagnosis, confirmation of severity, and treatment advice. All patients with protocol-defined significant AEs should have a photo-documentation of their lesions. For instructions on photo-documentation, please refer to the ISF. For specific management and grading guidelines for patients developing rash and photosensitivity reactions, please cf. Appendix 10.2 and Dermatologic Operations Manual in the ISF.

Grade 1/mild skin AEs are not considered *"protocol-defined significant AEs"* and should be reported in the AE eCRF and skin eCRF. Skin AEs with a known etiology (e.g. viral exanthema, collagen vascular disease, neoplasia, bacterial infection, psoriasis, fungal skin infection, or autoimmune blistering disease) deemed not related to the trial medications are not considered *"protocol-defined significant AEs"* and should be reported in the AE eCRF page only. A skin eCRF page is not required.

Protocol-defined significant AEs will be subject to review by the dermatological adjudication committee.

BI has set up a list of AEs which are defined to be always serious. In order to support the investigator with the identification of these "always serious adverse events", if a non serious AE is identified to be serious per BI definition, a query will be raised. The investigator must verify the description and seriousness of the event. If the event description is correct, the item "serious" needs to be ticked and an SAE has to be reported in expedited fashion following the same procedure as above. The list of these AEs can be found via the RDC-system.

5.2.3 Assessment of safety laboratory parameters

The safety laboratory will be performed at the central laboratory service provider. Reference ranges and instructions on sample collection, handling/processing, and shipping will be provided in the ISF.

Patients should be fasting for at least 6 hours prior to the blood sample being taken (except screening visit). For time points of sampling refer to the Table 5.2.3: 1 and Flow Chart. For blinding of selected laboratory results cf. Section 4.1.5.1.

Clinically relevant laboratory values should be commented on lab report print-outs. A clinically relevant value may be either in- or outside the reference range. Clinically relevant abnormal laboratory test results must be confirmed using an unscheduled visit lab kit and should be repeated until normalisation or stabilisation or until an alternative explanation has been found.

Proprietary confidential information.
© 2012 Boehringer Ingelheim International GmbH or one or more of its affiliated companies. All rights reserved.
This document may not - in full or in part - be passed on, reproduced, published or otherwise used without prior written permission.

Proprietary confidential information © 2015 Boehringer Ingelheim International GmbH or one or more of its affiliated companies

The following laboratory results will be flagged by the central laboratory for further assessment of potential DILI cases:

- ALT or AST 2x baseline that is at least 100 IU/L higher than baseline,
- Total bilirubin >3x ULN combined with direct /indirect bilirubin >1

Proprietary confidential information.
© 2012 Boehringer Ingelheim International GmbH or one or more of its affiliated companies. All rights reserved.
This document may not - in full or in part - be passed on, reproduced, published or otherwise used without prior written permission.

Proprietary confidential information © 2015 Boehringer Ingelheim International GmbH or one or more of its affiliated companies

Table 5.2.3: 1 Laboratory tests

Category	Test name	
**Haematology**	Hematocrit (Hct)<br>Hb<br>RBC Count/Erythrocytes<br>Reticulocyte Count<br>WBC Count/Leukocytes<br>Platelet Count/Thrombocytes	
	Diff. Automatic (manual if diff. automatic is abnormal)	- Neutrophils<br>- Eosinophils<br>- Basophils<br>- Monocytes<br>- Lymphocytes
**Coagulation**	Partial Thromboplastin Time (aPTT)<br>Prothrombin time (Quick and INR)	
**Chemistry**	AST (GOT)<br>ALT (GPT)<br>Alkaline Phosphatase (AP)<br>Albumin<br>Creatine Kinase (CK)<br>CK-MB, only if CK is elevated<br>Gamma-Glutamyl Transferase (GGT/γ-GT)<br>Lactic Dehydrogenase (LDH)<br>Lipase<br>Amylase<br>Calcium<br>Sodium<br>Potassium<br>Chloride<br>Bicarbonate<br>Glucose<br>Creatinine<br>Creatinine clearance (calculated)<br>Bilirubin Total[4]<br>Bilirubin Direct[4]<br>Bilirubin Indirect[4]<br>Protein, Total<br>C-Reactive Protein<br>Cholesterol, total<br>Triglycerides<br>Haptoglobin, , only if Hb <10.5g/dL<br>Bile acids<br>Urea	

1 At Visit 1 only
2 At liver disease progression assessment time-points
3 Results will not be captured in the sponsor’s database
4 DAIDS grading does not apply
5 IgE only in case of moderate or severe rash

Proprietary confidential information.
© 2012 Boehringer Ingelheim International GmbH or one or more of its affiliated companies. All rights reserved.
This document may not - in full or in part - be passed on, reproduced, published or otherwise used without prior written permission.

Proprietary confidential information © 2015 Boehringer Ingelheim International GmbH or one or more of its affiliated companies

Table 5.2.3: 1 (continued) Laboratory tests

Category	Test name
**Pregnancy test**	Human serum chorionic gonadotropin (all visits)<br>Human urine chorionic gonadotropin (at Visit 2 only before the first study drugs intake)
**Urine-Sediment (microscopic examination), (only if urine analysis abnormal)**	Urine Sediment Bacteria<br>Urine Cast in Sediment<br>Urine Squamous Epithelial Cells<br>Urine Sed. Crys., Unspecified Urine Sediment RBC/Erythrocytes<br>Urine Sediment WBC/Leucocytes
**Tumour markers**[2]	Alpha-fetoprotein
**Autoimmune serum markers**[1]	Anti-nuclear antibodies (ANA)
**Other laboratory parameters**[1]	IP-10 (IFN-gamma inducible protein-10)<br>Glycosylated Hb (HbA1c)<br>Homeostatic model assessment of insulin resistance (HOMA-IR)<br>Parathyroid hormone<br>Vitamin D measurement<br>LDL, VLDV and HDL
**Drug screening (urine)**[1,3]	Cannabis<br>Benzodiazepine<br>Barbiturates<br>Opiates<br>Cocaine<br>Amphetamines<br>Methadone
**HCV, HBV, HIV testing**	Hepatitis B Surface Antigen (qualitative) [1,3]<br>Hepatitis C Antibodies (qualitative)<br>HIV-1, and HIV-2 Antibody (qualitative) [1,3]<br>HCV RNA PCR (quantitative)
**Specific immunoglobulin (Ig) measurement**	Total serum IgE[5]

1 At Visit 1 only
2 At liver disease progression assessment time-points
3 Results will not be captured in the sponsor’s database
4 DAIDS grading does not apply
5 IgE only in case of moderate or severe rash

5.2.3.1 Pregnancy tests

Serum pregnancy tests will be performed at screening (Visit 1) and at every visit for all female patients. In addition to serum pregnancy test at all study visits, a urine (dipstick) pregnancy test will be performed before receiving the study drugs at Day 1 (Visit 2). Only female patients with negative urine pregnancy test will receive study medication at Day 1 (Visit 2). Urine (dipstick) pregnancy tests will not be captured in the database.

When visits are more than 4 weeks apart and/or due to early treatment termination, female patients will be provided with urine pregnancy tests to perform monthly pregnancy tests at home between visits until 7 months after EOT. It is strongly recommended that female partners of male patients also perform monthly pregnancy test at home and the sponsor will provide urine (dipstick) pregnancy tests for this purpose.

Proprietary confidential information.
© 2012 Boehringer Ingelheim International GmbH or one or more of its affiliated companies. All rights reserved.
This document may not - in full or in part - be passed on, reproduced, published or otherwise used without prior written permission.

Proprietary confidential information © 2015 Boehringer Ingelheim International GmbH or one or more of its affiliated companies

5.2.4 Electrocardiogram

The 12-lead ECGs will be performed as scheduled in the Flow Chart.

It will be recorded after the patients have rested for at least 5 minutes in supine position at Visit 1. Six limb leads, as specified by Einthoven (I, II and III) and Goldberger (aVR, aVL, aVF), and six pre-cordial leads (V1–V6), according to Wilson, will be used. The investigator or a designate will evaluate whether the ECG is normal or abnormal and, if abnormal, whether it is clinically significant. Additionally, any occurrence of re- or depolarization disorders, arrhythmic disorders or other abnormalities will be assessed.

The electronic version of the ECG is regarded as source data. Dated and signed printouts will be stored in the patient's medical file.

ECGs may be repeated for quality reasons and the repeat used for analysis. Additional ECGs may be collected by the investigator for safety reasons. Clinically relevant abnormal findings will be reported as AEs.

5.2.5 Physical examination

Complete and target physical examinations will be carried out as described in the Flow Chart.

A complete physical exam consists of an evaluation of organ systems with vital signs (including respiratory rate, temperature, pulse rate, and systolic/diastolic blood pressure). Respiratory rate, temperature, pulse rate, and blood pressure will be measured after patients have been sitting comfortably for at least five minutes.

A targeted physical examination includes an evaluation of organ systems only associated with an AE symptom(s) (as needed,and particularly focused on hepatic-related events) and evaluation of vital signs.

Clinically relevant abnormal findings will be reported as AEs.

5.2.6 FibroSURE™

Non-invasive method for lived fibrosis assessment, FibroSURE™, will be assessed at the time-points specified in the Flow Chart. FibroSURE™ is an algorithm based on a composite of five serum biochemical markers (alpha-2-macroglobulin, apolipoprotein A1, haptoglobin, GGT and bilirubin) that is used to assess liver fibrosis with its different degrees. The algorithm generates a score that correlates with the level of fibrosis and the presence of cirrhosis.

Proprietary confidential information.
© 2012 Boehringer Ingelheim International GmbH or one or more of its affiliated companies. All rights reserved.
This document may not - in full or in part - be passed on, reproduced, published or otherwise used without prior written permission.

Proprietary confidential information © 2015 Boehringer Ingelheim International GmbH or one or more of its affiliated companies

5.3 OTHER

5.3.1 Other endpoints

5.3.1.1 Health Care Resource Utilization (HCRU) and Work Productivity

For the purpose of a separate health economic analysis (such as cost-utility analysis), HCRU data will be collected throughout the study. Resource use data collected for calculating direct costs will include unscheduled hospitalisations, healthcare provider visits, and emergency room/ICU use. Information on work productivity will also be collected.

The economic evaluation of the HCRU data will not be part of the CTR but reported separately.

5.3.1.2 EQ-5D

Health related quality of life will be assessed using the EQ-5D at the visits indicated on the Flow Chart. EQ-5D is a standardized instrument for use as a measure of health outcome. It is a generic measure, rather than disease-specific, and is therefore applicable to a wide range of health conditions and treatments. It provides a simple descriptive profile and a single index value for health status. EQ-5D is designed for self completion by patients.

The EQ-5D self-report questionnaire (EQ-5D) essentially consists of 2 pages comprising:

- The descriptive system (five dimensions of health; namely mobility, self care, usual activities, pain/discomfort, anxiety/depression). Each dimension comprises three levels (no problems, some/moderate problems, extreme problems).

- The EQ-VAS (visual analogue scale) which records the patient's self-rated health status on a vertical graduated (0 – 100) VAS.

The paper-and-pen version in the required native language of the patient will be used. If the required language is not available then the patient is not required to complete the questionnaire.

A patient can self-administer the EQ-5D in a few minutes, and the Investigator (or designated site-personnel) should ensure that the patient has access to a quiet area at the site where he can be left alone to record a response to the descriptive system and VAS. In instances where a patient cannot give or decide upon a response, no response should be recorded. The Investigator (or designated site-personnel) should check that all items have been completed by the patient, but the response to each item should not be scrutinized. Instructions to patients are included in the questionnaire.

Proprietary confidential information.
© 2012 Boehringer Ingelheim International GmbH or one or more of its affiliated companies. All rights reserved.
This document may not - in full or in part - be passed on, reproduced, published or otherwise used without prior written permission.

Proprietary confidential information © 2015 Boehringer Ingelheim International GmbH or one or more of its affiliated companies

5.3.2 Other assessments

5.3.2.1 Genotypic and Phenotypic Resistance

5.3.2.1.1 Genotypic Resistance

In this study of FDV and BI 207127 combination therapy, genotypic resistance monitoring (sequencing) will focus on the NS3/4A and NS5B genomic segments. Samples for genotyping the HCV NS3/4A protease and NS5B polymerase will be collected at screening (Visit 1) and at all patient visits. Viral genotyping will be performed for patients who discontinue study treatment. HCV RNA plasma viral load data will guide which additional samples are examined; genotyping will be performed on samples in which the HCV RNA plateaus above the lower limit of quantification or in which the HCV RNA rebounds during trial period (before or at EOO visit) and compared to baseline sequence. These include FU isolates (as detailed in Flow Chart) from individuals whose on- and post-treatment viral samples are genotyped to assess the persistence of resistance mutations during the outgrowth of wild-type virus.

The viral RNA will be extracted, cDNA synthesized and amplified using reverse transcriptase-PCR. The length of amplified product potentially limits the detection to samples with HCV RNA $>10^3$ IU/mL. The NS3/4A protease and NS5B polymerase nucleotide sequences will be obtained by direct DNA sequencing of the amplified product that allows for the detection of variants present at approximately ≥30%.

5.3.2.1.2 Phenotypic Resistance

The virology samples obtained for genotypic resistance monitoring are also suitable for phenotyping. NS3 protease or NS5B polymerase replicon-based phenotyping will be performed on samples in which the HCV RNA plateaus above the limit of quantification or in which the HCV RNA rebounds during trial period (before or at EOO visit) and which are associated with unique resistant mutants that have not been previously phenotyped. Representatives of the most common and well characterized pathways in assays will be included as references. In addition to patient-derived NS3 or NS5B samples, novel amino acid substitutions will also be characterized phenotypically using site-directed mutagenesis if they are observed repeatedly in genotyped subjects.

5.3.2.1.3 Methods and timing of sample collection

At least 4 mL of plasma from 8 mL of blood sample will be collected for virology samples (genotyping and phenotyping analyses) at each visit. These will be divided into two 2 mL aliquots: one primary and one back-up. Samples will be shipped to the central laboratory on dry ice. The central laboratory will store samples at -80°C until processing. Detailed instructions for obtaining, handling, and shipping virology samples are provided in ISF.

5.3.2.2 Assessment of liver disease progression

Markers of liver disease progression will be collected as occurrence of any of the following clinical outcomes and assessed at the time points specified in the Flow Chart:

Proprietary confidential information.
© 2012 Boehringer Ingelheim International GmbH or one or more of its affiliated companies. All rights reserved.
This document may not - in full or in part - be passed on, reproduced, published or otherwise used without prior written permission.

Proprietary confidential information © 2015 Boehringer Ingelheim International GmbH or one or more of its affiliated companies

- Child-Turcotte-Pugh score in points (cf. Appendix 10.3)
- MELD score in points
- New onset of ascites or change in severity from baseline
- New onset of hepatic encephalopathy or change in severity from baseline
- Liver cancer
- Esophageal variceal haemorrhage
- Spontaneous bacterial peritonitis
- Liver transplant
- Liver-related death

5.3.3 Pharmacogenomic evaluation

The first sample, a mandatory part of the protocol, collected at Visit 2 will be used for DNA extraction and subsequent genotyping of genotyping of IL-28b rs12979860, as well as other genes involved in the activity and/or efficacy of the drug within the context of the clinical trial. In addition, genes influencing assimilation and/or metabolism like UGT1A1, HLA class I and II molecules, CYP450 enzyme and drug transporter genes, like OATPs, genes influencing the therapeutic outcome like interferon signalling pathway genes and/or interferon stimulated genes may be evaluated. After successful analysis, the remaining material will be destroyed.

The second sample collected at Visit 2 sample is voluntary and will be taken and processed or stored only after a separate informed consent is given in accordance with local ethical and regulatory requirements. This sample will be completely anonymised. The anonymization procedure will guarantee a very high level of data protection for the donor. Once the anonymization has been carried out, there will be no way to trace back to the identity of the donor through the coding keys. The anonymised DNA may be analysed at a later time to identify whether there are genetic factors that could contribute to a better therapeutic outcome or a higher risk of developing treatment-related adverse drug reactions. These analyses may include genes related to efficacy, safety and PK. After anonymization, the third sample (or the DNA derived thereof) will be stored at Boehringer Ingelheim for 15 years after the end of the clinical trial or until there is no more material available for tests.

5.3.3.1 Methods and timing of sample collection

One mandatory and one optional 8.5mL blood sample will be taken at Visit 2 for pharmacogenetic testing with PaxGene DNA blood sampling tubes. The Paxgene Blood DNA tubes can be stored and shipped at room temperature within 14 days. If a longer storage and shipment period for Paxgene Blood DNA tubes is necessary, the blood samples have to be stored at a temperature of -20° C or below. Once frozen, thawing of the samples should be avoided. Detailed instructions for pharmacogenetic sampling, handling and shipment of samples are provided in the ISF.

Proprietary confidential information.
© 2012 Boehringer Ingelheim International GmbH or one or more of its affiliated companies. All rights reserved.
This document may not - in full or in part - be passed on, reproduced, published or otherwise used without prior written permission.

Proprietary confidential information © 2015 Boehringer Ingelheim International GmbH or one or more of its affiliated companies

5.3.3.2 Analytical determinations

Genomic DNA will be extracted from blood samples according to standard molecular genetics methods and analyzed by TaqMan® or other standard genotyping technologies.

5.4 APPROPRIATENESS OF MEASUREMENTS

All measurements performed during this trial are standard measurements in HCV treatment trials and will be performed in order to monitor safety aspects or assess treatment response in an appropriate way.

The scheduled measurements are appropriate to see drug induced changes in vital signs, standard laboratory values and ECG. These primary and secondary endpoints are standard and accepted for evaluation of safety and tolerability of an oral drug, and they are widely used in this kind of studies.

Therefore, the appropriateness of all measurements applied in this trial is given.

5.5 DRUG CONCENTRATION MEASUREMENTS AND PHARMACOKINETICS

5.5.1 Pharmacokinetic endpoints

The following primary PK parameters will be calculated for patients participated in Cohort A:

- C_{max} (maximum measured concentration of BI 207127 & metabolites, FDV and RBV)
- AUC_{0-12} (area under the concentration-time curve of BI 207127 & metabolites and RBV in plasma over the time interval from 0 to 12 hour)
- AUC_{0-24} (area under the concentration-time curve of FDV in plasma over the time interval from 0 to 24 hour)
- C_{12} (plasma concentration of BI 207127 & metabolites and RBV at 12 hour)
- C_{24} (plasma concentration of FDV at 24 hour)

Other PK parameters will be determined for the analyte if feasible in Cohort A:

- t_{max} (time to reach maximum concentration C_{max} in plasma)
- λ_z (terminal rate constant in plasma)
- $t_{1/2}$ (terminal half-life of the analyte in plasma)
- MRT_{po} (mean residence time of the analyte in the body)

Proprietary confidential information.
© 2012 Boehringer Ingelheim International GmbH or one or more of its affiliated companies. All rights reserved.
This document may not - in full or in part - be passed on, reproduced, published or otherwise used without prior written permission.

Proprietary confidential information © 2015 Boehringer Ingelheim International GmbH or one or more of its affiliated companies

- CL/F (apparent clearance of the analyte in the plasma after extravascular administration)
- V_z/F (apparent volume of distribution during the terminal phase λ_z)

In addition plasma protein binding of BI 207127 & metabolites and FDV will be determined in Cohort A only.

5.5.2 Methods of sample collection

The date and clock time of the most recent drug intakes, and date and clock time of the blood sample collection will be entered in the eCRF. Further details on collection, preparation, and shipping will be included in ISF.

5.5.2.1 Pharmacokinetic samples

For Cohort A, intensive PK blood samples will be collected at Day 1, 8 and Week 4 and 16 for all patients. At visits with intensive PK sample collection, PK blood samples are collected at -0.05 (pre-dose), 0.5, 1, 1.5, 2, 3, 4, 5, 6, 8, 10, 12 and 24 hours after dosing (cf. Appendix 10.1.2, Table 10.1.2: 1).

For Cohort B, an intensive PK sub-study will be conducted to assess pre-dose and 4 post-dose samples at Day 1, 8 and Week 4 and 16 (cf. Appendix 10.1.2, Table 10.1.2: 2). A total of 48 patients will participate and 16 patients will be assigned to each of the following collection schedules via IRT: Day 1 and Day 8; Day 1 and Week 4; or Day 1 and Week 16 (cf. Section 4.1.2). The 4 post-dose samples will be taken at specific time period post-morning dose according to a pseudo-randomised scheme described in Appendix 10.1.2, Table 10.1.2: 2. The data from intensive PK sub-study for Cohort B will be tranferred to Pharmacometrics group in Translationational Medicine department for population pharmacokinetic analysis.

For all patients in Cohort A and Cohort B, pre-dose PK blood samples will be collected according to the Flow Chart. If patient is subjected to intensive PK on same visit day, only collect intensive PK samples. Patients should not take study medication the mornings of treatment period visits. Administration of the study drug will be done under the supervision of the investigator or his designee after the pre-dose sample has been collected. The time elapsed since the last dose of FDV should be as close to 24 hours as possible and the last dose of BI 207127 should be as close to 12 hours as possible.

For quantification of FDV and RBV plasma concentration, 2.0 mL of blood will be drawn from a forearm vein in an EDTA-anticoagulant blood drawing tube. For quantification of BI 207127, CD 6168, BI 208333 (BI 207127 acylglucuride) and CD 6168-acylglucuronide, 4.5 mL of blood will be drawn from a forearm vein in a blood drawing tube containing citrate or citrate and citric acid.

Proprietary confidential information.
© 2012 Boehringer Ingelheim International GmbH or one or more of its affiliated companies. All rights reserved.
This document may not - in full or in part - be passed on, reproduced, published or otherwise used without prior written permission.

Proprietary confidential information © 2015 Boehringer Ingelheim International GmbH or one or more of its affiliated companies

5.5.2.2 Protein binding

For determination of protein binding of BI 207127 & metabolites and FDV (Arm 1, 2 and 3 in Cohort A only), two 10 mL blood samples will be drawn from a forearm vein in EDTA-anticoagulant blood drawing tubes at 2.5 h post dose at Day 1,8 and Week 4 and 16. Due to the resource capacity, only samples collected at Day 1 and 8 will be first used for protein binding assay. Remaining samples will be analyzed only if needed, i.e. variability from the results of Day 1 and 8.

5.5.3 Analytical determinations

5.5.3.1 Pharmacokinetics

All analytes will be determined by a validated HPLC-MS/MS assay (high performance liquid chromatography, tandem mass spectrometry). Bioanalytical determinations for all the analytes will be performed by Tandem Labs (Salt Lake City, UT 84124).

FDV ZW: The concentration of FDV ZW in EDTA plasma will be determined by a validated HPLC/MS/MS (high performance liquid chromatography, tanden mass spectrometry) method.

BI 207127: The concentrations of BI 207127 and its metabolites in citrated/citric acid plasma will be determined by a validated HPLC/MS/MS (high performance liquid chromatography, tandem mass spectrometry) method.

RBV: The concentrations of RBV in EDTA plasma will be determined by a validated HPLC/MS/MS (high performance liquid chromatography, tandem mass spectrometry) method.

Proprietary confidential information.
© 2012 Boehringer Ingelheim International GmbH or one or more of its affiliated companies. All rights reserved.
This document may not - in full or in part - be passed on, reproduced, published or otherwise used without prior written permission.

Proprietary confidential information © 2015 Boehringer Ingelheim International GmbH or one or more of its affiliated companies

6. INVESTIGATIONAL PLAN

6.1 VISIT SCHEDULE

All patients are to adhere to the visit schedule as specified in the Flow Chart. Each visit date (with its window) is to be counted from Day 1. Additional visits for the purpose of re-testing of laboratory parameters or AE monitoring may be included as deemed necessary by the investigator.

6.2 DETAILS OF TRIAL PROCEDURES AT SELECTED VISITS

For detailed description of the trial procedures to be performed at each visit, refer to the Flow Chart and respective protocol sections. Explanations of procedures are provided in Section 5. Additional details regarding visit procedures are provided below.

All visits, except Visit 1 (screening), must be performed in fasted state.

6.2.1 Screening and run-in periods

No procedures should be done unless the patient has provided written informed consent in accordance with GCP and local legislation to taking part in the trial.

Once consented, a patient is considered to be enrolled in the trial and have started screening. The patient should be assigned a patient number, recorded on the enrolment log, registered in IRT, and recorded on the eCRF as a screened patient.

Screening (Visit 1) should normally take place no more than 42 days before Visit 2; however the time window for Visit 1 may be extended at the discretion of the CML in conjunction with the TCM on a case by case basis.

Re-screening a patient will not be permitted. Patients who have a laboratory test value outside the range specified by the entry criteria may have the test repeated once to determine eligibility and the result must be available prior to Visit 2 (Day 1). Screening visit (Visit 1) activities may be extended to two physical visits if needed.

Prior liver biopsy or fibroscan results will be recorded in the eCRF as medical history.

Patients who fail screening following Visit 1 procedures should be registered as a screen failure in IRT.

6.2.2 Treatment period

The treatment period is from Visit 2 to EOT Visit. Patients will be dispensed medication at visits according to the Flow Chart and new medication unit number(s) will be provided through the IRT on each occasion.

Instruct patient NOT to take study medication on the morning of all visits.

Proprietary confidential information.
© 2012 Boehringer Ingelheim International GmbH or one or more of its affiliated companies. All rights reserved.
This document may not - in full or in part - be passed on, reproduced, published or otherwise used without prior written permission.

Proprietary confidential information © 2015 Boehringer Ingelheim International GmbH or one or more of its affiliated companies

Administration of the morning dose of study drug will be done under the supervision of the investigator or his designee after the pre-dose PK sample has been collected. Note that the routine laboratory samples are to be collected in fasted condition and only afterwards patients must take their medication with food.

6.2.2.1 Randomization/treatment assignment visit (Visit 2)

Patients will begin treatment as assigned by the IRT at Visit 2 with the first dose administered in the office (Day 1) including an additional 120 mg loading dose of FDV (or placebo) (cf. Section 4.1.4).

For Cohort B, IRT will assign mandatory PK sub-study participation (cf. Section 5.5).

6.2.2.2 Visit 3 to Visit 12

For Cohort B, results of plasma HCV RNA levels and a series of safety laboratory tests after start of the treatment will be blinded to investigators, patients and sponsor (cf. Section 4.1.5.1). The Investigator will be informed by the central laboratory when a criteria for treatment discontinuation due to lack of virologic response (cf. Section 3.3.4.1) are met for a given patient during this period.

6.2.2.3 EOT Visit

Planned EOT visit will occur at Week 24 according to the planned treatment duration. These patients should be registered as completed in IRT.

Patients who discontinue treatment early due to either lack of efficacy or safety reasons will be required to have an EOT visit (if the patient is at the research site), or have EOT visit scheduled within 2 weeks from the last intake of study medication. These patients should be registered in IRT as early discontinuation of trial medication. Please note that if either BI 207127 or FDV is permanently discontinued, all study medication including RBV must be discontinued immediately (cf. Section 4.2.1).

For Cohort B, the sponsor, patient, and investigator will be unblinded to all laboratory values and treatment (active or placebo) for each patient at their EOT visit.

6.2.3 End of trial and follow-up period

Termination of trial medication and trial completion must be recorded on the corresponding eCRFs for all randomised/treatment assigned patients.

6.2.3.1 Follow-up (FU)

If HCV RNA is detected during a FU visit, a subsequent confirmatory testing should be performed.

If a patient receives any other HCV treatment outside this protocol during the FU period the patient will need to be discontinued from the trial and have EOO visit prior to the start of that

Proprietary confidential information.
© 2012 Boehringer Ingelheim International GmbH or one or more of its affiliated companies. All rights reserved.
This document may not - in full or in part - be passed on, reproduced, published or otherwise used without prior written permission.

Proprietary confidential information © 2015 Boehringer Ingelheim International GmbH or one or more of its affiliated companies

treatment. Efficacy data collected after the start of such treatment will not be attributed to the trial treatment. Any subsequent end-points will be considered as not achieving treatment response.

Patients with EOT at Week 24

Patients who successfully complete the 24-week treatment period should return to the clinic for FU visits according to the Flow Chart. FU1 visit will occur 4 weeks after the discontinuation of trial treatment. This is the last at which the investigator has the responsibility to report every AE. During the next FU visits, only SAEs fatal or considered by the investigators as related to study medication will be reported. Refer to Flow Chart and Section 5.2.2.

Patient with early treatment termination

Patients who stop study treatment prior to completion of the treatment period should return to the clinic for all FU visits per the Flow Chart starting with a FU1 visit 4 weeks after EOT.

It is understood that a patient may withdraw consent at any time and decide not to complete FU, but such FU is to be encouraged.

6.2.3.2 End of Observation

EOO will be performed for all patients at 96 weeks after EOT.

Patients who discontinue the FU period early will be required to have an EOO visit (if the patient is at the research site), or have EOO visit scheduled within 3 weeks from discontinuation of the FU.

6.2.3.3 Treatment Option for Arm 4

For patients that participated in Arm 4, an option to received trial treatment according to Arm 5 may be offered, if patient is still eligible for treatment (cf. Section 3.1). The trial Flow Chart will not be followed; instead these patients will follow the treatment visit schedule and procedures as listed in Table 4.2.1: 2. Refer to ISF for further details on the procedures to follow and unblinding.

Proprietary confidential information.
© 2012 Boehringer Ingelheim International GmbH or one or more of its affiliated companies. All rights reserved.
This document may not - in full or in part - be passed on, reproduced, published or otherwise used without prior written permission.

Proprietary confidential information © 2015 Boehringer Ingelheim International GmbH or one or more of its affiliated companies

7. STATISTICAL METHODS AND DETERMINATION OF SAMPLE SIZE

7.1 STATISTICAL DESIGN - MODEL

7.1.1 Design

Cohort A:

This design is a multi-national, three-arm, open label trial. Fifteen CPA patients will be treated with 600 mg BI 207127 b.i.d. in combination with 120 mg FDV q.d. and RBV (Arm 1); 15 CPB patients will be treated with 400 mg BI 207127 b.i.d. in combination with 120 mg FDV q.d. and RBV (Arm 2); If patients in Arm 2 do not reach the target exposure, Arm 3 of 15 CPB patients treated with 600 mg BI 207127 b.i.d. in combination with 120 mg FDV q.d. and RBV.

Cohort B:

This design is a multi-national, randomised, double-blind, placebo controlled, parallel-group trial (cf. Section 3.1). Moderate hepatic impairment (CPB) patients with HCV genotype 1b infection will be randomised in a 1:3 ratio to placebo:active treatment.

7.2 NULL AND ALTERNATIVE HYPOTHESES

Cohort A:

No hypothesis testing will be performed. The results will be presented descriptively.

Cohort B:

The null hypothesis is that the incidence of SVR12 in the active treated group is equal to that in the placebo group. The alternative hypothesis is that the two incidences are unequal, with the goal to demonstrate a higher SVR12 rate in the active-treatment group than in the placebo group. The hypothesis will be tested using alpha=0.05 (2-sided) with at least 80% of power.

7.3 PLANNED ANALYSES

The primary analyses of efficacy will be carried out on an intent-to-treat basis including all patients who are randomised and receive at least one dose of assigned therapy during the trial.

The interim analysis for Cohort A to determine BI 207127 dose in Cohort B will be performed after all Arm 1 and 2 patient reach Week 4 and, if needed, after all Arm 3 patients reach Week 4.

The primary analysis for Cohort A and B will be performed after all patients complete FU2 (assessment of SVR12, the primary efficacy endpoint).

Proprietary confidential information.
© 2012 Boehringer Ingelheim International GmbH or one or more of its affiliated companies. All rights reserved.
This document may not - in full or in part - be passed on, reproduced, published or otherwise used without prior written permission.

Proprietary confidential information © 2015 Boehringer Ingelheim International GmbH or one or more of its affiliated companies

The final analysis will be performed after all patients complete their last planned visit, including data from Cohort B placebo arm patients that received active treatment.

7.3.1 Primary analyses

Cohort A:

PK analysis will be performed after Day 1, 8, Week 4 and 16 intensive PK sample collection for Cohort A. For details of the analysis, see Section 7.3.4.

Cohort B:

For the primary analysis, Fisher's exact test will be used for the hypothesis testing.

SVR12 will be assessed based on the observed HCV RNA result taken at least 8 weeks (to allow an appropriate visit window) after treatment discontinuation. This definition will also hold for patients that discontinue treatment early, i.e. if the patient has HCV RNA undetected at least 8 weeks after stop of all treatment, they will be considered a responder in the primary analysis.

If a patient has detectable HCV RNA at the FUP2 visit but had undetectable HCV RNA at the previous visit, a confirmation sample will be used to determine SVR12. This procedure will also be implemented for patients who have multiple observations in the SVR12 window (≥10 weeks after the planned treatment period) – that is, if a patient has a detectable HCV RNA after being undetectable, confirmation will be required before classifying the patient as a SVR12 failure. Patients that take other HCV therapies after stopping all trial medication will also be considered SVR12 failure in the primary analyses.

7.3.2 Secondary analyses

Sensitivity analyses of the primary endpoint will be performed for the per-protocol set and completers set to assess the impact of important protocol violations and premature discontinuation on the primary endpoint.

A further sensitivity analysis will be performed imputing missing SVR data by last available post-treatment result (SVR12).

The secondary endpoints SVR4 and SVR24 will be evaluated with the same statistical method that is described for the primary endpoint in Section 7.3.1.

Ninety-five percent confidence intervals (CIs) will be reported for the differences in response rates between treatment groups for SVR12, SVR4, and SVR24.

7.3.3 Safety analyses

All safety data will be displayed and analysed using descriptive statistical methods. No formal inferential analysis is planned for safety comparison

Proprietary confidential information.
© 2012 Boehringer Ingelheim International GmbH or one or more of its affiliated companies. All rights reserved.
This document may not - in full or in part - be passed on, reproduced, published or otherwise used without prior written permission.

Proprietary confidential information © 2015 Boehringer Ingelheim International GmbH or one or more of its affiliated companies

AEs will be coded using the Medical Dictionary for Drug Regulatory Activities (MedDRA) coding dictionary. All events with an onset after the first dose of study medication up to a period of 28 days after the last dose of study medication will be assigned to the treatment phase for evaluation. Other AEs will be assigned either to the screening or FU phase as appropriate. Frequency tables of patients who experience AEs leading to discontinuation will be provided. Kaplan-Meier curves might be used to investigate the time to an event for considerations of special interest, for example rash event. More details of these analyses will be included in the TSAP.

Laboratory values taken after the first dose of randomised treatment up to a period of 7 days after the last intake of treatment will be assigned to the treatment phase for evaluation. Frequency tables with abnormal laboratory values defined by DAIDS grades (R10-1332) and, if necessary, the proportion exceeding multiples (2*, 3* …) of the upper normal range limit will be provided. Changes in bilirubin (direct, indirect, total) values will be described in the context of being associated with clinical events. The absolute value and the change from baseline in laboratory values will be summarized by treatment group. Baseline is defined as the last observed measurement prior to administration of any randomised study medication.

A DMC will be set up with primary responsibility to review the safety database on an ongoing basis. Refer to Section 3.1.1 for more details.

7.3.4 Interim analyses

For Cohort A, PK parameters AUC_{0-12}, Cmax, C_{12} of BI 207127 and AUC_{0-24}, Cmax, C_{24} of FDV will be calculated and provided to evaluate the systemic exposure of BI 207127 and FDV in CPB patients after receiving 400 mg BI 207127 compared to BI 207127 and FDV in CPA patients after receiving 600 mg BI 207127. Geometric mean of difference, sd, 90% CI will be calculated for these parameters between treatment arms. The results will be presented descriptively and no statistical testing will be performed. The lower bound of the 90% CI for the geometric mean test arm (Arm 1): control arm (Arm 2) of C_{12} will be compared to 0.5 while the upper bound will be compared to 2. The boundaries were derived based on SOUND-C2 results. Whether the lower BI 207127 dose treatment in Arm 1 will provide proper exposure to CPB patients will be decided by assessment of the descriptive statistics listed above along with the efficacy and safety profile of the patients by an independent internal review committee (cf. Section 3.1.1). This committee will either select a dose for Cohort B or determine that Arm 3 should be conducted and the same analysis performed comparing Arm 1 to Arm 3.

7.3.5 Pharmacokinetic analyses

Cohort A:

Refer to Section 7.3.4.

Cohort B:

The PK parameters AUC_{0-12}, C_{max}, C_{12} of BI 207127 and AUC_{0-24}, C_{max}, C_{24} of FDV will be calculated after Day 1, 8, Week 4 and 16 of intensive PK sample collection.

Proprietary confidential information.
© 2012 Boehringer Ingelheim International GmbH or one or more of its affiliated companies. All rights reserved.
This document may not - in full or in part - be passed on, reproduced, published or otherwise used without prior written permission.

Proprietary confidential information © 2015 Boehringer Ingelheim International GmbH or one or more of its affiliated companies

7.3.6 Other analyses

Ninety-five percent CIs will be reported for the differences in response rates between treatment groups for $W4U_{TND}$, W4U, $W12U_{TND}$, and ETR_{TND}.

Time to achieving HCV RNA undetected and time to virologic failure will be analyzed using Cox model.

The relationship between patient response and exposure will be investigated. HCV RNA change from baseline over time will be regressed on corresponding BI 201335 and BI 207127 concentrations to assess saturation of the exposure-response relationship.

Biochemical response and liver disease progression will be presented descriptively.

7.4 HANDLING OF MISSING DATA

All randomised patients who discontinue from the trial without reaching the SVR time points will be counted as treatment failures. Patients with missing SVR4 will be imputed by SVR12, if available. Patients with missing SVR12 will be imputed by SVR24, if available. Likewise, patients with missing SVR24 will be left as missing at the time of the interim database lock. Sensitivity analyses for missing SVR12 values due to early discontinued patients will be described in the TSAP.

It is not planned to impute missing values for safety data with the exception that missing AE start and end dates will be estimated (details to be written up in the TSAP).

7.5 RANDOMISATION

Cohort A:

Eligible patients will be assigned to a treatment arm according the patient's Child Pugh score and the current screening stage of the trial. No randomisation is needed. All treatment arms will require a minimum of 8 females per arm.

Cohort B:

Eligible patients will be randomly assigned to one of the two treatment regimens to avoid possible biases due to subjective patient selection. An allocation ratio of 1:3 for Arm 4 and Arm 5 is planned.

The randomisation list will be generated using a validated system, which involves a pseudorandom number generator to guarantee the reproducibility of the assignments. This randomisation list will be checked by an independent statistician and used by a third-party IRT system to assign randomisation numbers to eligible patients.

Proprietary confidential information.
© 2012 Boehringer Ingelheim International GmbH or one or more of its affiliated companies. All rights reserved.
This document may not - in full or in part - be passed on, reproduced, published or otherwise used without prior written permission.

Proprietary confidential information © 2015 Boehringer Ingelheim International GmbH or one or more of its affiliated companies

7.6 DETERMINATION OF SAMPLE SIZE

Cohort A:

Aiming at equal trough between Arms 1 and 2, 15 patients per arm will provide reasonable power to detect differences when alpha = 0.1 and the lower equivalence bound of the trough ratio is set at 0.5 and upper bound set at 2 (Table 7.6: 1).

Table 7.6: 1 Power calculation for Cohort A (alpha = 0.1)

CV	Power
80%	83%
100%	67%
120%	52%

Computed with proc power of SAS version 9.2 (assuming log normal and using exact method)

Cohort B:

Thirty patients will be recruited for the placebo group and 90 will be recruited for the active treatment group. The response rate for the placebo group is expected to be zero. For calculation purpose, it is set to 1%. The reference response rate for the active treatment group has not been set yet because no such treatment combination was tested on such patient population. The trial will have a power of 81% to detect, with a 2-sided alpha=0.05, an SVR12 incidence rate of 20% on active treatment group using Fisher's exact test. If the incidence rate reaches 30%, the power will be greater than 99% (Table 7.6: 2).

Table 7.6: 2 Power calculation for Cohort B (incidence rate in placebo group is set at 1%, 2-sided alpha = 0.05)

Power	Incidence rate of active treatment group
81%	20%
95%	25%
>99%	30%

Computed with NQuery version 6.01(routine PTT2U)

Proprietary confidential information.
© 2012 Boehringer Ingelheim International GmbH or one or more of its affiliated companies. All rights reserved.
This document may not - in full or in part - be passed on, reproduced, published or otherwise used without prior written permission.

Proprietary confidential information © 2015 Boehringer Ingelheim International GmbH or one or more of its affiliated companies

8. INFORMED CONSENT, DATA PROTECTION, TRIAL RECORDS

The trial will be carried out in compliance with the protocol, the principles laid down in the Declaration of Helsinki, in accordance with the ICH Harmonised Tripartite Guideline for GCP and relevant BI SOPs. Standard medical care (prophylactic, diagnostic and therapeutic procedures) remains in the responsibility of the treating physician of the patient.

The investigator should inform the sponsor immediately of any urgent safety measures taken to protect the study subjects against any immediate hazard, and also of any serious breaches of the protocol/ICH GCP.

The rights of the investigator and of the sponsor with regard to publication of the results of this trial are described in the investigator contract. As a general rule, no trial results should be published prior to finalisation of the CTR.

Insurance Cover: The terms and conditions of the insurance cover are made available to the investigator and the patients via documentation in the ISF.

8.1 STUDY APPROVAL, PATIENT INFORMATION, AND INFORMED CONSENT

This trial will be initiated only after all required legal documentation has been reviewed and approved by the respective IRB / IEC and CA according to national and international regulations. The same applies for the implementation of changes introduced by amendments.

Prior to patient participation in the trial, written informed consent must be obtained from each patient (or the patient’s legally accepted representative) according to ICH GCP and to the regulatory and legal requirements of the participating country. Each signature must be personally dated by each signatory and the informed consent and any additional patient-information form retained by the investigator as part of the trial records. A signed copy of the informed consent and any additional patient information must be given to each patient or the patient’s legally accepted representative.

The patient must be informed that his/her personal trial-related data will be used by Boehringer Ingelheim in accordance with the local data protection law. The level of disclosure must also be explained to the patient.

The patient must be informed that his / her medical records may be examined by authorised monitors (CML/CRA) or Clinical Quality Assurance auditors appointed by Boehringer Ingelheim, by appropriate IRB / IEC members, and by inspectors from regulatory authorities.

8.2 DATA QUALITY ASSURANCE

A quality assurance audit/inspection of this trial may be conducted by the sponsor or sponsor’s designees or by IRBs / IECs or by regulatory authorities. The quality assurance auditor will have access to all medical records, the investigator’s trial-related files and correspondence, and the informed consent documentation of this clinical trial.

Proprietary confidential information.
© 2012 Boehringer Ingelheim International GmbH or one or more of its affiliated companies. All rights reserved.
This document may not - in full or in part - be passed on, reproduced, published or otherwise used without prior written permission.

Proprietary confidential information © 2015 Boehringer Ingelheim International GmbH or one or more of its affiliated companies

8.3 RECORDS

Case Report Forms (CRFs) for individual patients will be provided by the sponsor, either on paper or via remote data capture (RDC). See Section 4.1.5.2 for rules about emergency code breaks. For drug accountability, refer to Section 4.1.8.

8.3.1 Source documents

Source documents provide evidence for the existence of the patient and substantiate the integrity of the data collected. Source documents are filed at the investigator's site.

Data entered in the eCRFs that are transcribed from source documents must be consistent with the source documents or the discrepancies must be explained. The investigator may need to request previous medical records or transfer records, depending on the trial; also current medical records must be available.

For eCRFs all data must be derived from source documents.

8.3.2 Direct access to source data and documents

The investigator / institution will permit trial-related monitoring, audits, IRB / IEC review and regulatory inspection, providing direct access to all related source data / documents. CRFs/eCRFs and all source documents, including progress notes and copies of laboratory and medical test results must be available at all times for review by the sponsor's clinical trial monitor, auditor and inspection by health authorities (e.g. FDA). The CRA / on site monitor and auditor may review all CRFs/eCRFs, and written informed consents. The accuracy of the data will be verified by reviewing the documents described in Section 8.3.1.

8.4 LISTEDNESS AND EXPEDITED REPORTING OF ADVERSE EVENTS

8.4.1 Listedness

To fulfill the regulatory requirements for expedited safety reporting, the sponsor evaluates whether a particular AE is "listed", i.e. is a known side effect of the drug or not. Therefore a unique reference document for the evaluation of listedness needs to be provided. For the BI 207127 and FDV this is the current version of the IB (U06-1740 and U04-3332). For RBV this is EU SmPC. The current versions of these reference documents are to be provided in the ISF. No AEs are classified as listed for matching placebo, study design, or invasive procedures.

8.4.2 Expedited reporting to health authorities and IECs / IRBs

Expedited reporting of SAEs, e.g. SUSARs, to health authorities and IECs / IRBs, will be done according to local regulatory requirements. Further details regarding this reporting procedure are provided in the ISF.

Proprietary confidential information.
© 2012 Boehringer Ingelheim International GmbH or one or more of its affiliated companies. All rights reserved.
This document may not - in full or in part - be passed on, reproduced, published or otherwise used without prior written permission.

Proprietary confidential information © 2015 Boehringer Ingelheim International GmbH or one or more of its affiliated companies

8.5 STATEMENT OF CONFIDENTIALITY

Individual patient medical information obtained as a result of this trial is considered confidential and disclosure to third parties is prohibited with the exceptions noted below. Patient confidentiality will be ensured by using patient identification code numbers.

Treatment data may be given to the patient's personal physician or to other appropriate medical personnel responsible for the patient's welfare. Data generated as a result of the trial need to be available for inspection on request by the participating physicians, the sponsor's representatives, by the IRB / IEC and the regulatory authorities.

Proprietary confidential information.
© 2012 Boehringer Ingelheim International GmbH or one or more of its affiliated companies. All rights reserved.
This document may not - in full or in part - be passed on, reproduced, published or otherwise used without prior written permission.

Proprietary confidential information © 2015 Boehringer Ingelheim International GmbH or one or more of its affiliated companies

9. REFERENCES

9.1 PUBLISHED REFERENCES

P97-2059 Fattovich G, et al. Morbidity and mortality in compensated cirrhosis type C: a retrospective follow-up study of 384 patients. Gastroenterology 1997;112(2):463-472.

R99-1243 Pugh RNH, Murray-Lyon IM, Dawson JL, Pietroni MC, Williams R. Transsection of the oesophagus for bleeding oesophageal varices. Br J Surg 1973;60(8):646-649.

P00-15027 Heathcote J. Antiviral therapy for patients with chronic hepatitis C. Semin Liver Dis. 2000. 20(2): 185-199.

R10-1332 Division of AIDS Regulatory Support Center (DAIDS RSC) Division of AIDS table for grading the severity of adult and pediatric adverse events (version 1.0, December, 2004 (clarification August 2009)). http://rsc.tech-res.com/safetyandpharmacovigilance/ (accessed June 22, 2010) (2009).

R10-2440 Child CG, Turcotte JG. Surgery and portal hypertension. In: Child CG, editor. The liver and portal hypertension. Philadelphia: Saunders; 1964. p. 50-64.

R12-0262 Incivek (telaprevir) film coated tablets (Vertex Pharmaceuticals) (US prescribing information, revised: 5/2011). 2011

R12-0263 Victrelis (boceprevir) capsules (Merck & Co) (US prescribing information, revised: 05/2011). 2011

9.2 UNPUBLISHED REFERENCES

U04-3332 Steinmann G. Investigator’s Brochure FDV NA, Chronic Hepatitis C Infection, 1220.P1. Current Version 12 10 Oct 2011.

U06-1740 Kukolj G, Boecher W, Wu K, Stachlewitz R, Sane R, Hinderliter J. Investigator´s Brochure BI 207127 NA, Chronic Hepatitis C Virus Infection 1241.P1. Current Version 7. 08 Dec 2011.

U11-3471-01 Mensa F, Nehmiz G, Sabo J. Summary report of interim analysis at Week 12, Trial 1241.21 Part 2. 12 Sep 2011.

U11-3472-01 Sabo J, Lehr T, Mensa F. Clinical pharmacology report. Trial 1241.21 Part 1. Snapshot archive version No. 1.4. 7 Dec 2011.

Proprietary confidential information.
© 2012 Boehringer Ingelheim International GmbH or one or more of its affiliated companies. All rights reserved.
This document may not - in full or in part - be passed on, reproduced, published or otherwise used without prior written permission.

Proprietary confidential information © 2015 Boehringer Ingelheim International GmbH or one or more of its affiliated companies

10. APPENDICES

10.1 PHARMACOKINETIC METHODS

10.1.1 Evaluation of Pharmacokinetic Parameters

For the calculation of PK parameters, only concentrations within the validated concentration range will be used. The actual sampling times will be used. For pre-dose samples, the actual sampling time will be set to zero.

Noncompartmental PK parameters will be determined using WinNonlin or another validated program.

C_{max} and t_{max}: Individual C_{max} and t_{max} values will be directly determined from the plasma concentration time profiles of each subject. If the same C_{max} concentration occurs at different time points, t_{max} is assigned to the first occurrence of C_{max}.

AUCs**:** The areas under the curve spanning various time intervals will be calculated using the linear up/log down algorithm. If a drug concentration is equal to or higher than the preceding concentration, the linear trapezoidal method will be used. If the drug concentration is smaller than the preceding concentration, the logarithmic method will be used.

Linear trapezoidal rule ($t_2 > t_1$ and $C_2 \geq C_1$):

The area of the trapezoid between the two data points (t_1, C_1) and (t_2, C_2) will be computed by:

$$AUC_{t1\text{-}t2} = 0.5 \times (t_2 - t_1) \times (C_1 + C_2)$$

Logarithmic trapezoid rule ($t_2 > t_1$ and $C_2 < C_1$):

The area of the trapezoid between the two data points (t_1, C_1) and (t_2, C_2) will be computed by:

$$AUC_{t1-t2} = \frac{(t_2 - t_1) \times (C_2 - C_1)}{\ln(C_2 / C_1)}$$

Estimation of λ_z: The apparent terminal rate constant λ_z will be estimated from a regression of ln(C) versus time over the terminal log-linear drug disposition portion of the concentration-time profiles. The log-linear profiles, which include the regression line through the terminal points, will be checked via visual inspection, and it will be determined whether the regression appropriately represents the terminal slope. Only data points that describe the terminal log-linear decline will be included in the regression. A minimum of three points will be used in the determination of λ_z. If the last concentration-time point increases, this time point may be included if the $t_{1/2}$ estimate is reasonable. If λ_z is not determinable then consequently only parameters not requiring λ_z will be reported. In addition, the lower ($t_{\lambda z,start}$) and upper ($t_{\lambda z,end}$) limit on time for values to be included in the calculation of λ_z will be listed.

Proprietary confidential information.
© 2012 Boehringer Ingelheim International GmbH or one or more of its affiliated companies. All rights reserved.
This document may not - in full or in part - be passed on, reproduced, published or otherwise used without prior written permission.

Proprietary confidential information © 2015 Boehringer Ingelheim International GmbH or one or more of its affiliated companies

$t_{1/2}$: The terminal half-life will be calculated from the terminal rate constant using the equation:

$$t_{1/2} = \frac{\ln 2}{\lambda_z}$$

MRT_{po}: The mean residence time after extravascular administration (MRT_{po}) will be calculated as follows:

$$MRT_{po} = \frac{AUMC_{0-\infty}}{AUC_{0-\infty}}$$

The area under the first moment curve from time 0 to infinity ($AUMC_{0-\infty}$) is calculated according to:

$$AUMC_{0\text{-}\infty} = AUMC_{0\text{-tz}} + \frac{C'_{tz} \times t_z}{\lambda_z} + \frac{C'_{tz}}{\lambda_z^2}$$

CL/F: The apparent clearance after extravascular administration will be determined according to the following equation:

$$\text{CL or CL/F} = \frac{\text{Dose}}{AUC_{0-\infty}}$$

(F = absolute bioavailability factor)

V_z/F: The apparent volume of distribution during the terminal phase after extravascular administration (at steady state) will be determined according to the following equation:

$$V_z/F = \frac{CL/F}{\lambda_z}$$

The geometric mean (gMean) and coefficient of variation, gCV (given in %), will be calculated by the formulae:

$$gMean = \exp\left[\frac{1}{n}\sum_{i=1}^{n}\ln(x_i)\right] = \exp\left[\overline{\ln(x_i)}\right]$$

$$gCV(\%) = 100 \cdot \sqrt{\exp\left[Var(\ln(x_i))\right] - 1}$$

where

$$Var(\ln(x_i)) = \frac{1}{n-1}\sum_{i=1}^{n}\left[\ln(x_i) - \overline{\ln(x_i)}\right]^2$$

Proprietary confidential information.
© 2012 Boehringer Ingelheim International GmbH or one or more of its affiliated companies. All rights reserved.
This document may not - in full or in part - be passed on, reproduced, published or otherwise used without prior written permission.

Proprietary confidential information © 2015 Boehringer Ingelheim International GmbH or one or more of its affiliated companies

10.1.2 Blood sampling scheme for Pharmacokinetics

Table 10.1.2: 1 Blood sampling scheme for intensive PK at Day 1, 8 and Week 4 and 16 in Cohort A (Arms 1, 2 and 3)

Visit	Planned Time [h:min]	Clock Time (of the actual day) [h:min]	PK Plasma Sample	Protein Binding sample	Administration of the study drug
2, 3,6, 11	-0:05	07:55	X[2]		
	0:00	08:00			X[1]
	0:30	08:30	X		
	1:00	09:00	X		
	1:30	09:30	X		
	2:00	10:00	X		
	2:30	10:30		X	
	3:00	11:00	X		
	4:00	12:00	X		
	5:00	13:00	X		
	6:00	14:00	X		
	8:00	16:00	X		
	10:00	18:00	X		
	12:00	20:00	X		
	24:00	08:00	X		

Footnotes:

1 Administration of the study drug will be done under the supervision of the investigator or his designee after the pre-dose sample has been collected. The time elapsed since the last dose of FDV should be as close to 24 hours as possible and the last dose of BI 207127 should be as close to 12 hours as possible.

2 Pre-dose blood sample is collected within 10 minutes prior to the first drug administration of the day (nominal time 08:00).

Proprietary confidential information.
© 2012 Boehringer Ingelheim International GmbH or one or more of its affiliated companies. All rights reserved.
This document may not - in full or in part - be passed on, reproduced, published or otherwise used without prior written permission.

Proprietary confidential information © 2015 Boehringer Ingelheim International GmbH or one or more of its affiliated companies

Table 10.1.2: 2 Blood sampling scheme for PK sub-study visits in Cohort B (Arm 4 and Arm 5)

**Patient month of birth**[1]	**Blood sample collection time window**[2] **(hours relative to first drug administration of the day)**[3]				
	**Sample 1**[4]	**Sample 2**[5]	**Sample 3**[5]	**Sample 4**[5]	**Sample 5**[5]
January	-0.05 (pre-dose)	0.5 - 1.5	3 - 4	5.5 - 6.5	8 - 9
February	-0.05 (pre-dose)	1 – 2	3.5 - 4.5	6 - 7	8.5 - 9.5
March	-0.05 (pre-dose)	1.5 - 2.5	4 - 5	6.5 - 7.5	9 - 10
April	-0.05 (pre-dose)	0.5 - 1.5	3 - 4	5.5 - 6.5	8 - 9
May	-0.05 (pre-dose)	1 – 2	3.5 - 4.5	6 - 7	8.5 - 9.5
June	-0.05 (pre-dose)	1.5 - 2.5	4 - 5	6.5 - 7.5	9 - 10
July	-0.05 (pre-dose)	0.5 - 1.5	3 - 4	5.5 - 6.5	8 - 9
August	-0.05 (pre-dose)	1 – 2	3.5 - 4.5	6 - 7	8.5 - 9.5
September	-0.05 (pre-dose)	1.5 - 2.5	4 - 5	6.5 - 7.5	9 -10
October	-0.05 (pre-dose)	0.5 - 1.5	3 - 4	5.5 - 6.5	8 - 9
November	-0.05 (pre-dose)	1 – 2	3.5 - 4.5	6 - 7	8.5 - 9.5
December	-0.05 (pre-dose)	1.5 - 2.5	4 - 5	6.5 - 7.5	9 - 10

Footnotes:

[1] Pseudo-randomised scheme based on patient’s birth month. PK samples are collected at different time windows depending on birth month of patient as specified in the table above.

[2] For collection of PK sub-study samples, patients will be assignment via IRT at Visit 2 to one of the following collection schedules: Day 1 and Day 8; Day 1 and Week 4; or Day 1 and Week 16.

[3] Administration of the study drug will be done under the supervision of the investigator or his designee after the pre-dose sample has been collected. The time elapsed since the last dose of FDV should be as close to 24 hours as possible and the last dose of BI 207127 should be as close to 12 hours as possible.

[4] Pre-dose blood sample (Sample 1) is collected within 10 minutes prior to the first drug administration of the day (nominal time 08:00).

[5] Post-dose blood samples (Sample 2 to Sample 5) are collected at any time within the collection window and are relative to the first drug administration of the day (nominal time 08:00).

Proprietary confidential information.
© 2012 Boehringer Ingelheim International GmbH or one or more of its affiliated companies. All rights reserved.
This document may not - in full or in part - be passed on, reproduced, published or otherwise used without prior written permission.

Proprietary confidential information © 2015 Boehringer Ingelheim International GmbH or one or more of its affiliated companies

10.2 DERMATOLOGY MANAGEMENT PLAN

10.2.1 Introduction and scope

Drug-induced skin reactions (rash and photosensitivity reactions) have been reported with the use of FDV and BI 207127 combination therapy. The aim of this plan is to provide guidance for the management of suspected or confirmed drug-induced skin reactions. This section is not intended to provide guidance for skin events with known aetiologies in which a drug-relatedness has been ruled out (e.g. measles, skin cancer, scabies, etc).

For the management of skin rash cases refer to Section 10.2.3 and of photosensitivity reactions refer to Section 10.2.4. Skin rash cases may be reported as rash, or other specific diagnoses, e.g. urticaria, exanthema, etc. Photosensitivity reactions may be reported as phototoxic reaction, sunburn, etc.

10.2.2 General measures for skin protection

Patients should be instructed to protect any uncovered skin area (including hands, face and lips) from sun- or UV-light exposure using sun-blocker cream and lip balm with an SPF ≥50 providing UV-A and -B protection during treatment period on a daily basis. Patients should avoid unnecessary or prolonged exposure to sunlight and wear protective clothing, sunglasses and hats in addition to sun-block. It is recommended to avoid direct sun exposure as much as possible. Tanning booths must be avoided during treatment period.

All patients should be instructed to report all skin reactions and be seen by study medical staff as soon as possible. If a patient has photosensitivity reaction, the patient should be instructed to be even more consistent with the measures described earlier and stay away from windows as much as possible.

10.2.3 Drug-induced skin rash

These skin reactions may manifest with a multitude of clinical presentations and variable severity, ranging from mild rashes to life-threatening reactions such as Steven-Johnson Syndrome (SJS), toxic epidermal necrolysis (TEN) or acute generalized exanthematous pustulosis (AGEP).

10.2.3.1 Definition of severity for skin rash*

* definition of severity based on the DAIDS grading of rash (Division of AIDS Table for Grading the Severity of Adult and Pediatric Adverse Events), version 1.0 as of December 2004 – Clarification AUGUST 2009.

Mild (Grade I):

Localized macular or maculopapular rash.

Moderate (Grade II):

Diffuse macular, maculopapular, or morbilliform rash; or, target lesions.

Severe (Grade III):

Proprietary confidential information.
© 2012 Boehringer Ingelheim International GmbH or one or more of its affiliated companies. All rights reserved.
This document may not - in full or in part - be passed on, reproduced, published or otherwise used without prior written permission.

Proprietary confidential information © 2015 Boehringer Ingelheim International GmbH or one or more of its affiliated companies

Diffuse macular, maculopapular, or morbilliform rash with vesicles; limited number of bullae; or, superficial ulcerations of mucous membrane limited to one site.

Potentially Life-Threatening (Grade IV):

Extensive or generalized bullous lesions; SJS; ulceration of mucous membrane involving two or more distinct mucosal sites; or, TEN.

10.2.3.2 Management guidelines of skin rash

Mild:

Medium to high potency topical corticosteroids (potentially combined with oral antihistamines) should be prescribed as early as possible. Other non-prescription remedies such as oatmeal baths may be used to provide temporary relief of skin sensitivity and pruritus.

The need for UV light protection should be reinforced (Section 10.2.2).

Treatment with study medication may be continued without interruption.

Moderate:

The patient should have a detailed photo-documentation of the skin, preferably prior to the administration of any therapy. The subject should be seen by a dermatologist as soon as possible, but no later than 72 hours for diagnosis, confirmation of severity and treatment. A non-progressive moderate skin rash, confirmed by the dermatologist should be closely and frequently monitored at the investigator's discretion taking into account recommendation by dermatologist.

Medium to high potency topical corticosteroids combined with oral antihistamines should be prescribed as early as possible, but preferably after detailed photo-documentation of the skin lesions. Other non-prescription remedies such as oatmeal baths may be used to provide temporary relief of skin sensitivity and pruritus. Systemic corticosteroids may be applied if deemed clinically necessary.

The need for UV light protection should be reinforced (Section 10.2.2).

These reactions do not require discontinuation of study treatment.

Severe:

Medium to high potency topical corticosteroids combined with oral antihistamines should be prescribed as early as possible. Systemic corticosteroids may be applied if deemed clinically necessary. Subjects should have a detailed photo-documentation of the skin and referred to a dermatologist immediately but no later than 24 hours for diagnosis, confirmation of severity, treatment and skin biopsy. These patients must be closely monitored.

Proprietary confidential information.
© 2012 Boehringer Ingelheim International GmbH or one or more of its affiliated companies. All rights reserved.
This document may not - in full or in part - be passed on, reproduced, published or otherwise used without prior written permission.

Proprietary confidential information © 2015 Boehringer Ingelheim International GmbH or one or more of its affiliated companies

The decision to stop or continue the study treatment should be based on the clinical judgement of the investigator taking into account the recommendation by the dermatologist.

Potentially Life-Threatening

Patients with potentially life-threatening skin reactions should be referred immediately for emergency care. Subjects should stop all study medications. Patients should be seen by a dermatologist for diagnosis, confirmation of severity, treatment and skin biopsy. The patient should be followed up as appropriate.

10.2.4 Photosensitivity reactions

Photosensitivity reactions typically resemble exaggerated sunburn with erythema and edema often occurring within minutes to hours of sun exposure, and may involve vesiculation and desquamation in some cases. These photosensitivity reactions affect exclusively the sun-exposed skin areas; therefore, a clear delimitation of the erythema by garments is typically observed.

10.2.4.1 Definition of severity of photosensitivity reactions

Mild: Any localized superficial 1st degree burn: erythema and edema, peeling.

Moderate: Extensive 1st degree sunburn (erythema, edema, peeling), or localized 2nd degree sunburn with blistering, serous exudates.

Severe: Extensive or full face 2nd degree sunburn with blistering, serous exudates.

10.2.4.2 Management guidelines for photosensitivity reactions

Symptomatic management of photosensitivity reaction of any severity includes cool compresses, soothing lotions, topical corticosteroids and/or systemic non-steroidal anti-inflammatory drugs (NSAID) or antipruritics. The need for UV light protection should be reinforced (Section 10.2.2).

Mild:

Treatment with study medication may be continued without interruption.

Moderate:

The patient should have a detailed photo-documentation of the skin and should be seen by a dermatologist as soon as possible but no later than 72 hours for diagnosis, confirmation of severity, and treatment. Confirmed moderate photosensitivity reactions should be closely and frequently monitored at the investigator's discretion taking into account recommendation by dermatologist. These reactions do not require study treatment discontinuation.

Proprietary confidential information.
© 2012 Boehringer Ingelheim International GmbH or one or more of its affiliated companies. All rights reserved.
This document may not - in full or in part - be passed on, reproduced, published or otherwise used without prior written permission.

Proprietary confidential information © 2015 Boehringer Ingelheim International GmbH or one or more of its affiliated companies

Severe:

The subject should have a detailed photo-documentation of the skin and referred to a dermatologist immediately and within 24-48 hours for diagnosis, confirmation of severity, treatment and skin biopsy. These patients must be closely monitored. The decision to stop or continue the study treatment should be based on the clinical judgement of the investigator taking into account the recommendation by the dermatologist.

Proprietary confidential information.
© 2012 Boehringer Ingelheim International GmbH or one or more of its affiliated companies. All rights reserved.
This document may not - in full or in part - be passed on, reproduced, published or otherwise used without prior written permission.

Proprietary confidential information © 2015 Boehringer Ingelheim International GmbH or one or more of its affiliated companies

10.3 CHILD-TURCOTTE-PUGH CLASSIFICATION

Child-Turcotte-Pugh class and score (R99-1243 and R10-2440) employ five clinical measures of liver disease in the setting of liver cirrhosis: bilirubin, albumin, blood coagulation (as determined by prothrombin time [PT] or by INR), evidence of ascites, and evidence of encephalopathy.

To calculate the score, the limits presented in the table below are used.

Measure	1 Point	2 Points	3 Points
Bilirubin (mg/dL)*	<2*	2-3*	>3*
Albumin (g/dL)	>3.5	2.8-3.5	<2.8
Blood coagulation<br>PT: seconds prolonged<br>INR	 1-3<br><1.7	 4-6<br>1.7-2.3	 >6<br>>2.3
Ascites	None	Slight, or controlled medically	Moderate or severe
Encephalopathy	None	Grade 1-2	Grade 3-4

*For assessment of liver disease progress at Visit 6, Visit 8, Visit 10 and Visit 12, an increase in points due to increase in bilirubin value should only be added to the Child-Turcotte-Pugh score if the patient has a ratio of direct bilirubin/total bilirubin > 0.5.

For Child-Turcotte-Pugh class determination the scores are added and classified as:

- A: 5-6 points
- B: 7-9 points
- C: 10-15 points

Proprietary confidential information.
© 2012 Boehringer Ingelheim International GmbH or one or more of its affiliated companies. All rights reserved.
This document may not - in full or in part - be passed on, reproduced, published or otherwise used without prior written permission.

Proprietary confidential information © 2015 Boehringer Ingelheim International GmbH or one or more of its affiliated companies

11. DESCRIPTION OF GLOBAL AMENDMENTS

This is the original protocol.

**Number of global amendment**		
**Date of CTP revision**		
**EudraCT number**		
**BI Trial number**		
**BI Investigational Product(s)**		
**Title of protocol**		
**To be implemented only after approval of the IRB / IEC / Competent Authorities**		☐
**To be implemented immediately in order to eliminate hazard – IRB / IEC / Competent Authority to be notified of change with request for approval**		☐
**Can be implemented without IRB / IEC / Competent Authority approval as changes involve logistical or administrative aspects only**		☐
**Section to be changed**		
**Description of change**		
**Rationale for change**		

Proprietary confidential information.
© 2012 Boehringer Ingelheim International GmbH or one or more of its affiliated companies. All rights reserved.
This document may not - in full or in part - be passed on, reproduced, published or otherwise used without prior written permission.

Proprietary confidential information © 2015 Boehringer Ingelheim International GmbH or one or more of its affiliated companies

CO-ORDINATING INVESTIGATOR SIGNATURE

Trial Title: **A phase II randomised, double-blind and placebo-controlled study of BI 207127 in combination with faldaprevir and ribavirin in patients with moderate hepatic impairment (Child-Pugh B) with genotype 1b chronic hepatitis C infection**

Trial Number: **1241.30**

Protocol Version: **1**

I herewith certify that I agree to adhere to the trial protocol and to all documents referenced in the trial protocol.

Date: 13.12.12

Name: Michael P. Manns, Ph.D.,M.D. Signature: ____________

Affiliation:

Zentrum Innere Medizin
Medizinische Hochschule Hannover
Carl-Neuberg-Straße 1
30625 Hannover

Signed signature page is located in the electronic Clinical Trial Master File

A Klinische Forschung BC
FB Immunologie/Virologie
15. Jan. 2013

Proprietary confidential information © 2015 Boehringer Ingelheim International GmbH or one or more of its affiliated companies

16.1.1.2 Revised protocol and protocol amendments 83
1241-0030--protocol--revision 3 84
clinical-trial-protocol-revision-3-signature-ci-manns 172
clinical-trial-protocol-revision-2-signature-ci-manns 173
ctp-revision-1-signature-ci-manns-2013-08-28 174

Proprietary confidential information © 2015 Boehringer Ingelheim International GmbH or one or more of its affiliated companies

Clinical Trial Protocol (Revision 3)

Doc. No.: c02347351-01
Legacy Doc. No.: U12-3886-04

EudraCT No.:	2012-003534-17
**BI Trial No.:**	1241.30
**BI Investigational Products:**	BI 207127 (deleobuvir) in combination with faldaprevir
**Title:**	A phase ~~III~~ **IIb** ~~randomised, double-blind and placebo-controlled~~ **open label** study of BI 207127 in combination with faldaprevir and ribavirin in patients with moderate hepatic impairment (Child-Pugh B) with genotype 1b chronic hepatitis C infection
**Clinical Phase:**	IIb/~~III~~
**Trial Clinical Monitor:**	Renee Kaste, Ph.D.<br>Boehringer Ingelheim Pharmaceuticals, Inc.<br>900 Ridgebury Road<br>Ridgefield, Connecticut 06877<br>Phone: (203) 791-6626<br>Fax: (203) 798-5433
**Co-ordinating Investigator:**	Michael P. Manns, Ph.D.,M.D.<br>Zentrum Innere Medizin<br>Medizinische Hochschule Hannover<br>Carl-Neuberg-Straße 1<br>30625 Hannover<br>Phone: +49 (0) 5 11 - 5 32 33 05<br>Fax: +49 (0) 5 11 - 5 32 48 96
**Status:**	**Revised Protocol based on Global Amendment #03**
**Version and Date:**	**Version: 4.0** **Date: 11 Jun 2014**

Page 1 of 86

Proprietary confidential information.
© 2014 Boehringer Ingelheim International GmbH or one or more of its affiliated companies. All rights reserved.
This document may not - in full or in part - be passed on, reproduced, published or otherwise used without prior written permission.

Proprietary confidential information © 2015 Boehringer Ingelheim International GmbH or one or more of its affiliated companies

CLINICAL TRIAL PROTOCOL SYNOPSIS

**Name of company:**<br><br>Boehringer Ingelheim		**Tabulated**<br>**Trial Protocol**	
**Name of finished product:**<br><br>NA			
**Name of active ingredient:**<br><br>BI 207127 in combination with faldaprevir			
**Protocol date:**<br>30 Nov 2012	**Trial number:**<br>1241.30		**Revision date:**<br>**11 Jun 2014**

**Title of trial:**	A phase ~~III~~ **IIb** ~~randomised, double-blind and placebo-controlled~~ **open label** study of BI 207127 in combination with faldaprevir and ribavirin in patients with moderate hepatic impairment (Child-Pugh B) with genotype 1b chronic hepatitis C infection
**Co-ordinating:**	Michael P. Manns, Ph.D., M.D.
**Trial sites:**	Multi-centre trial
**Clinical phase:**	IIb/~~III~~
**Objectives:**	The objective of Cohort A is to evaluate the safety and pharmacokinetic (PK) profile of BI 207127 (potentially two doses) in combination with 120 mg once daily (q.d.) FDV and weight-based RBV in a small group of patients with moderate hepatic impairment (Child-Pugh B [CPB]) compared to patients with mild hepatic impairment (Child-Pugh A [CPA]) ~~to define the BI 207127 dose to be used in Cohort B~~.<br>~~The objective of Cohort B is to assess efficacy, safety, and pharmacokinetics of 24-week treatment of the BI 207127 dose selected in Cohort A in combination with 120 mg once daily (q.d.) FDV and weight - based RBV in a larger group of chronically infected HCV GT1b patients with moderate hepatic impairment (CPB).~~
**Methodology:**	Cohort A: Open label~~; Cohort B: Randomised, double-blind, placebo-controlled~~
**No. of patients:**	
**total entered:**	Approximately ~~165~~ **30**
**each treatment:**	Cohort A (15 patients per arm)<br>Arm 1 CPA 600 mg BI 207127 b.i.d. + 120 mg FDV q.d. + RBV b.i.d.<br>Arm 2 CPB 400 mg BI 207127 b.i.d. + 120 mg FDV q.d. + RBV b.i.d.<br>~~Arm 3 CPB 600 mg BI 207127 b.i.d. + 120 mg FDV q.d. + RBV b.i.d.~~<br>~~Cohort B (90 active treatment, 30 placebo)~~<br>~~Arm 4 CPB BI 207127 placebo b.i.d. + FDV placebo q.d. + RBV placebo b.i.d~~<br>~~Arm 5 CPB 400 or 600 mg (based on outcome of cohort A) BI 207127 b.i.d. + 120 mg FDV q.d. + RBV b.i.d~~
**Diagnosis:**	Chronic HCV infection
**Main criteria for inclusion:**	Treatment naïve and experienced patients (prior relapse, interferon intolerant, and ~~[allowed in Cohort A only]~~ prior partial response).<br>Chronic HCV infection of genotype 1 (GT1), sub-GT1b virus only.<br>Liver cirrhosis defined as Metavir Grade=4 or Ishak Grade ≥5 on liver biopsy or liver stiffness of ≥13 kPa on fibroscan.

Proprietary confidential information.
© 2014 Boehringer Ingelheim International GmbH or one or more of its affiliated companies. All rights reserved.
This document may not - in full or in part - be passed on, reproduced, published or otherwise used without prior written permission.

Proprietary confidential information © 2015 Boehringer Ingelheim International GmbH or one or more of its affiliated companies

**Name of company:**<br><br>Boehringer Ingelheim<br>**Name of finished product:**<br><br>NA<br>**Name of active ingredient:**<br><br>BI 207127 in combination with faldaprevir	**Tabulated Trial Protocol**	
**Protocol date:** 30 Nov 2012 | **Trial number:** 1241.30		**Revision date:** 11 Jun 2014

**Test product:**	Faldaprevir (FDV)
**dose:**	120 mg q.d. (additional 120 mg loading dose for a total of 240 mg q.d. on Day 1)
**mode of admin.:**	Per os
**Test product:**	BI 207127
**dose:**	600 mg b.i.d.<br>400 mg b.i.d.
**mode of admin.:**	Per os
**Test product:**	Ribavirin
**dose:**	1000 or 1200 mg daily (weight-based b.i.d. dosing)
**mode of admin.:**	Per os
~~**Comparator products:**~~	~~BI 207127-matching placebo; FDV-matching placebo; ribavirin-matching placebo~~
~~**dose:**~~	~~NA~~
~~**mode of admin.:**~~	~~Per os~~
**Duration of treatment:**	24 weeks
**Criteria for efficacy:**	The primary endpoint is Sustained Virologic Response at Week 12 post-treatment (SVR12): Plasma HCV RNA level <25 IU/mL at 12 weeks after EOT.<br>The secondary efficacy endpoints are:<br>• SVR4: Plasma HCV RNA level <25 IU/mL at 4 weeks after EOT<br>~~• SVR24: Plasma HCV RNA level <25 IU/mL at 24 weeks after EOT~~
**Criteria for pharmacokinetics:**	Pharmacokinetic parameters for BI 207127 (and metabolites), FDV and ribavirin<br>Primary endpoints: Cmax, $AUC_{0\text{-}12}$, $AUC_{0\text{-}24}$, C12, C24
**Criteria for safety:**	Safety endpoints to be assessed in this trial include: AEs, including Division of Acquired Immunodeficiency Syndrome (DAIDS) grades, AEs leading to treatment discontinuation of all trial medications, SAEs, Events of liver disease progression, Laboratory test abnormalities by DAIDS grades, Change in laboratory test values over time.
**Statistical methods:**	No hypothesis testing will be performed. The results will be presented descriptively.<br>~~A null hypothesis of equal response rate of SVR12 between the treatment and placebo group will be tested by Fisher's exact test. Time to achieving HCV RNA undetected and time to virologic failure will be analyzed using the Cox model.~~

Proprietary confidential information.
© 2014 Boehringer Ingelheim International GmbH or one or more of its affiliated companies. All rights reserved.
This document may not - in full or in part - be passed on, reproduced, published or otherwise used without prior written permission.

Proprietary confidential information © 2015 Boehringer Ingelheim International GmbH or one or more of its affiliated companies

FLOW CHART

Study period	Screening	Treatment												Follow-up (FU)[13]					
Visit	1	2	3	4	5	6	7	8	9	10	11	12	EOT[12]	FU1	~~FU2/~~ EOO[14]	~~FU3~~	~~FU4~~	~~FU5~~	~~EOO~~[14]
Week	-6 to -1	0	1	2	3	4	6	8	10	12	16	20	24	EOT +4	EOT +12	~~EOT +24~~	~~EOT +48~~	~~EOT +72~~	~~EOT +96~~
Day	-42 to -7	1	8 ±2	15±2	22±2	29±2	43±2	57±2	71±2	85±2	113±2	141 ±2	169 ±2	EOT+28 ±7	EOT+84 -2/+7	~~EOT+168 -2/+7~~	~~EOT+336 ±14~~	~~EOT+504 ±14~~	~~EOT+672 ±21~~
Informed consent	X																		
Demographics, medical history and baseline conditions	X																		
HCV, HBV, HIV serology	X																		
Drug screening (urine)	X																		
Eligibility criteria	X	X																	
Randomisation via IRT		X																	
Pharmacogenomic sample[1]		X																	
Pregnancy screening[2]	X	X	X	X	X	X	X	X	X	X	X	X	X	X	X	~~X~~			
Physical examination[3]	X^C	X^T	X^T	X^T	X^T	X^T	X^T	X^T	X^T	X^T	X^T	X^T	X^C	X^C	X^T	~~X^T~~	~~X^T~~	~~X^T~~	~~X^T~~
12-lead ECG	X	X				X		X		X	X	X	X	X					
HCV RNA	X	X	X	X	X	X	X	X	X	X	X	X	X	X	X	~~X~~	~~X~~	~~X~~	~~X~~
Virology samples[4]	X	X	X	X	X	X	X	X	X	X	X	X	X	X	X	~~X~~	~~X~~	~~X~~	~~X~~
Laboratory tests[5]	X	X	X	X	X	X	X	X	X	X	X	X	X	X	X	~~X~~	~~X~~	~~X~~	~~X~~
Pre-dose PK sample[6]		X	X	X	X	X	X	X	X	X	X	X	X						
Intensive PK[7,~~8~~]		X	X			X					~~X~~								
Assess liver disease progression[9]	X	X				X		X		X			X	X	X	~~X~~	~~X~~	~~X~~	~~X~~
FibroSURE™		X								X			X		X	~~X~~	~~X~~	~~X~~	~~X~~
EQ-5D and HCRU[10]		X				X		X			X		X			~~X~~			
AEs	X	X	X	X	X	X	X	X	X	X	X	X	X	X	X[16]	~~X[16]~~	~~X[16]~~	~~X[16]~~	~~X[16]~~
Concomitant medication	X	X	X	X	X	X	X	X	X	X	X	X	X	X	X	~~X~~	~~X~~	~~X~~	~~X~~
Dispense trial medication		X[11]				X		X		X	X	X							
Compliance			X	X	X	X	X	X	X	X	X	X	X						
Termination of trial medication													X[15]						
Trial completion form															X				~~X~~

Proprietary confidential information.
© 2014 Boehringer Ingelheim International GmbH or one or more of its affiliated companies. All rights reserved.
This document may not - in full or in part - be passed on, reproduced, published or otherwise used without prior written permission.

Proprietary confidential information © 2015 Boehringer Ingelheim International GmbH or one or more of its affiliated companies

Footnotes:

1 One mandatory and one optional pharmacogenomic (PG) samples will be collected at Visit 2.

2 Serum pregnancy tests for females of childbearing potential will be performed at all marked study visits. At Visit 2, an additional urine (dipstick) pregnancy test will be performed for all females and only those female patients with a negative result will receive study medication. When visits are more than 4 weeks apart, female patients of childbearing potential will be provided with urine pregnancy tests to perform monthly pregnancy tests at home until 7 months after the last dose of ribavirin. Urine pregnancy test for male participants' female partners of child-bearing potential will be provided to perform monthly testing until 7 months after the last dose of ribavirin. Male patients will provide and ensure pregnancy testing of his female partner. It is the responsibility of the investigator to remind the male patients of this.

3 "X^{C}" is a complete physical examination. "X^{T}" is a targeted physical examination including measurements of vital signs and evaluation of organ systems particularly associated with AE(s) symptoms.

4 Plasma will be stored for analysis of genotypic and phenotypic viral resistance and other HCV viral tests.

5 For laboratory test details refer to Section 5.2.3. Patients should be fasting for at least 6 hours prior to the blood sample being taken (except screening visit).

6 Patients should not take study medication the mornings of study visits. Pre-dose PK samples for FDV, BI 207127 (and metabolites) and ribavirin will be collected during the clinic visit. Time and date of the last drug intake will be recorded for the purpose of using in PK analysis. If patient is subjected to intensive PK on same visit day, only collect intensive PK samples. For details refer to Section 5.5.2.1.

7 For all of Cohort A ~~and Cohort B~~ PK sub-study participants: intensive PK samples for FDV, BI 207127 (and metabolites) and ribavirin will be collected at multiple times after dosing. Refer to Appendix 10.1.2, Table 10.1.2: 1 for Cohort A ~~and Table 10.1.2: 2 for Cohort B PK sub-study~~.

~~8 Only for Cohort A, blood samples will be collected at 2.5 hours post dose at Day 1, Day 8, Week 4, and Week 16 for determination of protein binding. Refer to Appendix 10.1.2, Table 10.1.2: 1.~~

9 For details on assessment of liver disease progression refer to Section 5.3.2.2.

10 EQ-5D questionnaires: patient reported outcomes. Healthcare Resource Use (HCRU): Patient outpatient visits due to AEs, hospital visits (other than study scheduled visits) due to AEs, and hospitalizations due to AEs. Refer to Section 5.3.1.2.

11 Includes an additional 120 mg loading dose of FDV (or matching placebo) for a total of 240 mg taken at the site and observed by research staff on Day 1.

12 End of Treatment (EOT). Patients who discontinue treatment prematurely must perform an EOT visit within 2 weeks of last dose of study drug. Patients who discontinue treatment due to lack of efficacy will perform the EOT visit when the lack of efficacy criteria is confirmed. Patients must proceed according to the visit schedule with the subsequent follow up visits after EOT even if they had an early treatment discontinuation.

13 FU visits are counted from day after last intake of study medication (EOT), which in most cases will be EOT visit.

14 End of Observation (EOO).

15 Termination of trial medication may be earlier if the patient does not complete the full treatment course. Each trial medication termination will be recorded separately.

16 For AE assessment after FU1, SAEs only (cf. Section 5.2.2.2).

Proprietary confidential information.
© 2014 Boehringer Ingelheim International GmbH or one or more of its affiliated companies. All rights reserved.
This document may not - in full or in part - be passed on, reproduced, published or otherwise used without prior written permission.

Proprietary confidential information © 2015 Boehringer Ingelheim International GmbH or one or more of its affiliated companies

TABLE OF CONTENTS

TITLE PAGE 1

CLINICAL TRIAL PROTOCOL SYNOPSIS 2

FLOW CHART 4

TABLE OF CONTENTS 6

ABBREVIATIONS 10

1. INTRODUCTION 12
   - 1.1 MEDICAL BACKGROUND 12
   - 1.2 DRUG PROFILE 13

2. RATIONALE, OBJECTIVES, AND BENEFIT - RISK ASSESSMENT 16
   - 2.1 RATIONALE FOR PERFORMING THE TRIAL 16
   - 2.2 TRIAL OBJECTIVES 16
   - 2.3 BENEFIT - RISK ASSESSMENT 16

3. DESCRIPTION OF DESIGN AND TRIAL POPULATION..... 20
   - 3.1 OVERALL TRIAL DESIGN AND PLAN 20
     - 3.1.1 Administrative structure of the trial 21
       - 3.1.1.1 Data Monitoring Committee (DMC).....22
       - 3.1.1.2 Rash and Photosensitivity Adjudication Committee (RPAC) 23
   - 3.2 DISCUSSION OF TRIAL DESIGN, INCLUDING THE CHOICE OF CONTROL GROUPS.....23
   - 3.3 SELECTION OF TRIAL POPULATION 24
     - 3.3.1 Main diagnosis for study entry 25
     - 3.3.2 Inclusion criteria 25
     - 3.3.3 Exclusion criteria 27
     - 3.3.4 Removal of patients from therapy or assessments.....28
       - 3.3.4.1 Removal of individual patients.....28
       - 3.3.4.2 Discontinuation of the trial by the sponsor 30

4. TREATMENTS..... 31
   - 4.1 TREATMENTS TO BE ADMINISTERED.....31
     - 4.1.1 Identity of BI investigational products and comparator products 31
     - 4.1.2 Method of assigning patients to treatment groups 31
     - 4.1.3 Selection of doses in the trial.....32
     - 4.1.4 Drug assignment and administration of doses for each patient 32

Proprietary confidential information.
© 2014 Boehringer Ingelheim International GmbH or one or more of its affiliated companies. All rights reserved.
This document may not - in full or in part - be passed on, reproduced, published or otherwise used without prior written permission.

Proprietary confidential information © 2015 Boehringer Ingelheim International GmbH or one or more of its affiliated companies

4.1.5 Blinding and procedures for unblinding 33
4.1.5.1 Blinding 33
4.1.5.2 Procedures for emergency unblinding 35
4.1.6 Packaging, labelling, and re-supply 35
4.1.7 Storage conditions 35
4.1.8 Drug accountability 36
4.2 CONCOMITANT THERAPY AND RESTRICTIONS, AND RESCUE TREATMENT 36
4.2.1 Rescue medication, emergency procedures, and additional treatments 36
4.2.2 Restrictions 39
4.2.2.1 Restrictions regarding concomitant treatment 39
4.2.2.2 Restrictions on diet and life style 40
4.3 TREATMENT COMPLIANCE 40
5. VARIABLES AND THEIR ASSESSMENT 43
5.1 EFFICACY - CLINICAL PHARMACOLOGY 43
5.1.1 Endpoints of efficacy 43
5.1.2 Assessment of efficacy 44
5.2 SAFETY 44
5.2.1 Endpoints of safety 44
5.2.2 Assessment of adverse events 44
5.2.2.1 Definitions of adverse events 44
5.2.2.2 Adverse event and serious adverse event reporting 46
5.2.3 Assessment of safety laboratory parameters 48
5.2.3.1 Pregnancy tests 50
5.2.4 Electrocardiogram 51
5.2.5 Physical examination 51
5.2.6 FibroSURE™ 51
5.3 OTHER 52
5.3.1 Other endpoints 52
5.3.1.1 Health Care Resource Utilization (HCRU) and Work Productivity 52
5.3.1.2 EQ-5D 52
5.3.2 Other assessments 53
5.3.2.1 Genotypic and Phenotypic Resistance 53
5.3.2.1.1 Genotypic Resistance 53
5.3.2.1.2 Phenotypic Resistance 53
5.3.2.1.3 Methods and timing of sample collection 53
5.3.2.2 Assessment of liver disease progression 53
5.3.3 Pharmacogenomic evaluation 54

Proprietary confidential information.
© 2014 Boehringer Ingelheim International GmbH or one or more of its affiliated companies. All rights reserved.
This document may not - in full or in part - be passed on, reproduced, published or otherwise used without prior written permission.

Proprietary confidential information © 2015 Boehringer Ingelheim International GmbH or one or more of its affiliated companies

5.3.3.1 Methods and timing of sample collection 54
5.3.3.2 Analytical determinations 55
5.4 APPROPRIATENESS OF MEASUREMENTS 55
5.5 DRUG CONCENTRATION MEASUREMENTS AND PHARMACOKINETICS 55
5.5.1 Pharmacokinetic endpoints 55
5.5.2 Methods of sample collection 56
5.5.2.1 Pharmacokinetic samples 56
5.5.3 Analytical determinations 57
5.5.3.1 Pharmacokinetics 57
6. INVESTIGATIONAL PLAN 58
6.1 VISIT SCHEDULE 58
6.2 DETAILS OF TRIAL PROCEDURES AT SELECTED VISITS 58
6.2.1 Screening and run-in periods 58
6.2.2 Treatment period 58
6.2.2.1 Randomization/treatment assignment visit (Visit 2) 59
6.2.2.~~3~~2 EOT Visit 59
6.2.3 End of trial and follow-up period 59
6.2.3.1 Follow-up (FU) 59
6.2.3.2 End of Observation 60
7. STATISTICAL METHODS AND DETERMINATION OF SAMPLE SIZE 61
7.1 STATISTICAL DESIGN - MODEL 61
7.1.1 Design 61
7.2 NULL AND ALTERNATIVE HYPOTHESES 61
7.3 PLANNED ANALYSES 61
7.3.1 Primary analyses 62
7.3.2 Secondary analyses 62
7.3.3 Safety analyses 62
7.3.4 Interim analyses 63
7.3.5 Pharmacokinetic analyses 64
7.3.6 Other analyses 64
7.4 HANDLING OF MISSING DATA 64
7.5 RANDOMISATION 64
7.6 DETERMINATION OF SAMPLE SIZE 65
8. INFORMED CONSENT, DATA PROTECTION, TRIAL RECORDS 67

Proprietary confidential information.
© 2014 Boehringer Ingelheim International GmbH or one or more of its affiliated companies. All rights reserved.
This document may not - in full or in part - be passed on, reproduced, published or otherwise used without prior written permission.

Proprietary confidential information © 2015 Boehringer Ingelheim International GmbH or one or more of its affiliated companies

8.1 STUDY APPROVAL, PATIENT INFORMATION, AND INFORMED CONSENT 67
8.2 DATA QUALITY ASSURANCE 67
8.3 RECORDS 68
8.3.1 Source documents 68
8.3.2 Direct access to source data and documents....68
8.4 LISTEDNESS AND EXPEDITED REPORTING OF ADVERSE EVENTS....68
8.4.1 Listedness....68
8.4.2 Expedited reporting to health authorities and IECs / IRBs....68
8.5 STATEMENT OF CONFIDENTIALITY....69
9. REFERENCES 70
9.1 PUBLISHED REFERENCES....70
9.2 UNPUBLISHED REFERENCES....70
10. APPENDICES 71
10.1 PHARMACOKINETIC METHODS....71
10.1.1 Evaluation of Pharmacokinetic Parameters....71
10.1.2 Blood sampling scheme for Pharmacokinetics....73
10.2 DERMATOLOGY MANAGEMENT PLAN....75
10.2.1 Introduction and scope....75
10.2.2 General measures for skin protection....75
10.2.3 Drug-induced skin rash....75
10.2.3.1 Definition of severity for skin rash*....75
10.2.3.2 Management guidelines of skin rash....76
10.2.4 Photosensitivity reactions....77
10.2.4.1 Definition of severity of photosensitivity reactions....77
10.2.4.2 Management guidelines for photosensitivity reactions....77
10.3 CHILD-TURCOTTE-PUGH CLASSIFICATION....79
11. DESCRIPTION OF GLOBAL AMENDMENTS.... 80

Proprietary confidential information.
© 2014 Boehringer Ingelheim International GmbH or one or more of its affiliated companies. All rights reserved.
This document may not - in full or in part - be passed on, reproduced, published or otherwise used without prior written permission.

Proprietary confidential information © 2015 Boehringer Ingelheim International GmbH or one or more of its affiliated companies

ABBREVIATIONS

AE	Adverse Event
AGEP	Acute Generalized Exanthematous Pustulosis
ALT	Alanine Aminotransferase
AST	Aspartate Aminotransferase
AUC_{0-12}	Area under the concentration-time curve in plasma over the time interval from 0 to 12 hour
AUC_{0-24}	Area under the concentration-time curve in plasma over the time interval from 0 to 24 hour
BI	Boehringer Ingelheim
b.i.d.	bis in die (twice daily)
CA	Competent Authority
CI	Confidence Interval
C_{max}	Maximum concentration of an analyte in plasma or serum at steady state
CML	Local Clinical Monitor
CPA	Child-Pugh A
CPB	Child-Pugh B
CRA	Clinical Research Associate
CRF	Case Report Form
CTMF	Clinical Trial Master File
CTP	Clinical Trial Protocol
CTR	Clinical Trial Report
CYP	Cytochrome P450
DAA	Direct Acting Antiviral
DAIDS	Division of Acquired Immunodeficiency Syndrome
DRESS	Drug Rash with Eosinophilia and Systemic Symptoms
DMC	Data Monitoring Committee
eCRF	Electronic Case Report Form
EOO	End of Observation
EOT	End of Treatment
ESA	Erythropoiesis Stimulating Agent
ETR_{TND}	End of Treatment Response
EudraCT	European Clinical Trials Database
FDV	Faldaprevir
FU	Follow-up
GCP	Good Clinical Practice
G-CSF	Granulocyte-Colony Stimulating Factor
GI	Gastrointestinal
GT1	Genotype 1
Hb	Haemoglobin
HCV	Hepatitis C Virus
HCRU	Health Care Resource Utilization
IB	Investigator’s Brochure
IEC	Independent Ethics Committee
INR	International Normalized Ratio

Proprietary confidential information.
© 2014 Boehringer Ingelheim International GmbH or one or more of its affiliated companies. All rights reserved.
This document may not - in full or in part - be passed on, reproduced, published or otherwise used without prior written permission.

Proprietary confidential information © 2015 Boehringer Ingelheim International GmbH or one or more of its affiliated companies

IRB	Institutional Review Board
IRT	Interactive Response Tool
ISF	Investigator Site File
LFT	Liver function test
MedDRA	Medical Dictionary for Drug Regulatory Activities
MRI	Magnetic resonance imaging
OPU	Operative Unit
PD	Pharmacodynamics
PegIFN	Pegylated alfa interferon
P-gp	P-glycoprotein
PI	Principal Investigator
PK	Pharmacokinetic
q.d.	quaque die (once daily)
RBC	Red blood cell
RBV	Ribavirin
RNA	Ribonucleic acid
RPAC	Rash and Photosensitivity Adjudication Committee
SAE	Serious Adverse Event
SJS	Stevens-Johnson Syndrome
SOC	Standard of Care
SOP	Standard Operating Procedure
SUSAR	Suspected Unexpected Serious Adverse Reaction
SVR	Sustained virological response
$t_{1/2}$	Terminal half-life of the analyte in plasma/serum
TCM	Trial Clinical Monitor
TEN	Toxic epidermal necrolysis
t.i.d.	ter in die (three times daily)
t_{max}	Time to peak concentration
TSAP	Trial Statistical Analysis Plan
ULN	Upper limit normal
UV	Ultraviolet
WHO	World Health Organization
$W12U_{TND}$	Plasma HCV undetected at Week 12
W4U	Plasma HCV RNA level <25 IU/mL at Week 4
$W4U_{TND}$	Plasma HCV RNA undetected at Week 4

Proprietary confidential information.
© 2014 Boehringer Ingelheim International GmbH or one or more of its affiliated companies. All rights reserved.
This document may not - in full or in part - be passed on, reproduced, published or otherwise used without prior written permission.

Proprietary confidential information © 2015 Boehringer Ingelheim International GmbH or one or more of its affiliated companies

1. INTRODUCTION

1.1 MEDICAL BACKGROUND

Chronic infection with hepatitis C virus (HCV) represents a major public health problem worldwide. The World Health Organization (WHO) estimates that approximately 3% of the world population are infected. Approximately one third of those infected will develop cirrhosis in less than 20 years, and as many as 7% may develop hepatocellular carcinoma (P00-15027).

Different HCV genotypes prevail in different geographic regions, with genotype 1 (GT1) being the predominant genotype (70%) in United States, Asia, and Europe. Sub-GT1a is more prevalent in North and South America and common in Australia, while sub-GT1b is more prevalent in Europe and Asia.

Currently, two direct acting antiviral agents (DAAs) (HCV protease inhibitors, telaprevir and boceprevir) have been approved for HCV treatment in selected countries. Treatment with 12 weeks of telaprevir in combination with pegylated alfa interferon (PegIFN) and ribavirin (RBV) for either 24 or 48 weeks demonstrated sustained virologic response (SVR) rate of 74% or 79% at Week 24 (ILLUMINATE or ADVANCE studies, R12-0262). Boceprevir given for 24 or 44 weeks in combination with PegIFN/RBV achieved SVR rates of 63% to 66%, respectively (SPRINT-2 study, R12-0263). These combination therapies represent the new Standard of Care (SOC) for HCV patients with chronic GT1 infection with compensated liver disease (mild hepatic impairment or CPA). However, both these compounds are given three times daily (t.i.d.), are associated with substantial side effects, and are not approved regimens for patients with moderate hepatic impairment (CPB).

Despite the improved virologic response rates in patients with HCV GT1 infection following the recent approvals of new DAAs, the increasingly aging population with co-morbidities and contraindications to PegIFN, and the limiting side effects of this current SOC underscores the remaining urgent need for more effective, less toxic, shorter, more convenient and less invasive therapy for these patients. Effective antiviral therapy without PegIFN would constitute a major breakthrough in HCV treatment. PegIFN is contraindicated in CPB patients since it is poorly tolerated and associated with serious adverse events, high discontinuation rates and a faster liver progression in this population. Therefore, this patient population has a high unmet medical need and an interferon-free regimen may represent the only treatment option for patients with progressive liver cirrhosis or with HCV re-infection after liver transplantation.

BI is investigating the safety and efficacy of BI 207127, an oral, specific and reversible non-nucleoside HCV specific ribonucleic acid (RNA)-dependent RNA polymerase inhibitor, in combination with faldaprevir (FDV), a potent, oral HCV NS3/4a protease inhibitor, and RBV in patients with moderate hepatic impairment.

Proprietary confidential information.
© 2014 Boehringer Ingelheim International GmbH or one or more of its affiliated companies. All rights reserved.
This document may not - in full or in part - be passed on, reproduced, published or otherwise used without prior written permission.

Proprietary confidential information © 2015 Boehringer Ingelheim International GmbH or one or more of its affiliated companies

1.2 DRUG PROFILE

Detailed descriptions of drug substance, pharmacology, safety, pharmacology, toxicology and pharmacokinetics from preclinical and clinical studies are available in the current version of the Investigator's Brochures (IB) for BI 207127 (U06-1740) and FDV (U04-3332).

Faldaprevir (FDV)

FDV (also known as BI 201335) is a potent and specific HCV NS3/4A protease inhibitor. FDV is currently being evaluated in combination with PegIFN and RBV in Phase 3 of clinical development.

Clinical drug interaction studies of FDV with CYP3A4/5, CYP2C9 and P-gp have shown moderate inhibition of CYP3A4 and mild inhibition of CYP2C9. These drug interactions can be effectively managed. Specific recommendations for co-medications, including substrates of CYP3A4/5 or CYP2C9 which have a narrow therapeutic range, were developed with external clinical pharmacology experts and are provided in the Investigator Site File (ISF) (lists of restricted concomitant drugs and of drugs that need to be used with caution). For more information refer to the current IB version (U04-3332).

BI 207127

BI 207127 is a specific and reversible non-nucleoside inhibitor of HCV specific RNA-dependent RNA polymerase and is being developed in combination with FDV. Phase 2b evaluation of BI 207127 is ongoing in trial 1241.21 (SOUND-C studies) with Parts 1 and 2 complete and Part 3 in progress.

1241.21 Part 2 (SOUND-C 2) is a 5-arm, open-label, randomized, phase 2b study evaluating efficacy and safety of interferon-free oral combination regimens of BI 207127 and FDV with and without RBV for up to 40 weeks of treatment. A total of 362 treatment-naïve HCV GT1 patients were randomized and treated in 5 treatment arms. In the combined interim analysis by viral subtype (1a *vs.* 1b), the highest SVR12 (85%) was achieved with the twice daily (BID) 28-week regimen in GT1b infected patients (Table 1.2: 1). This is the dose and target population to be studied in the phase 3 program starting in parallel. In contrast, GT1a, and especially the sub-population of 1a_non-CC achieved sub-optimal SVR12 rates ranging from 42% to 0% (Table 1.2: 1). Among GT1b patients with compensated liver cirrhosis in SOUND-C2 randomized to RBV-containing regimens, the SVR rate was between 57% and 80%.

Proprietary confidential information.
© 2014 Boehringer Ingelheim International GmbH or one or more of its affiliated companies. All rights reserved.
This document may not - in full or in part - be passed on, reproduced, published or otherwise used without prior written permission.

Proprietary confidential information © 2015 Boehringer Ingelheim International GmbH or one or more of its affiliated companies

Table 1.2: 1 1241.21 SOUND-C2 SVR12 rate by patient sub-population

	GT1b	GT1a
16-week BI 207127 TID with FDV and RBV	35/47 (75%)	13/34 (38%)
28-week BI 207127 TID with FDV and RBV	33/48 (69%)	14/32 (44%)
40-week BI 207127 TID with FDV and RBV	24/43 (56%)	16/34 (47%)
28-week BI 207127 BID with FDV and RBV	41/48 (85%)	13/30 (43%)
28-week BI 207127 TID FDV without RBV	16/28 (57%)	2/18 (11%)

Among patients assigned to a BI 207127 600 mg t.i.d. regimen, patients randomised to 40 weeks experienced the highest rate of discontinuation due to adverse events (AEs). There was a 5%, 13% and 25% rate of early discontinuation due to AEs in the 16-week, 28-week and 40-week treatment arms, respectively. In the BI 207127 600 mg b.i.d. 28-week regimen, 8% patients discontinued due to AEs. As expected, the most frequent AEs reported on the FDV/ BI 207127 combination therapy are skin (rash, photosensitivity reactions) and gastrointestinal (GI) (nausea, vomiting and diarrhoea) events and indirect hyperbilirubinemia. Photosensitivity reactions and vomiting occurring during the first 10 days of treatment have been statistically associated with the extent of the exposure of the BI drugs.

BI 207127 and FDV plasma concentrations were higher when the two drugs are used in combination compared to when there are used as monotherapy or when combined with PegIFN and RBV. On-treatment, pre-dose plasma concentrations of both appeared to steadily decrease over the first few weeks of treatment reaching stable trough exposure approximately at treatment Week 4-6. In addition, a 2-fold increase in exposure of both FDV and BI 207127 in cirrhotic patients compared to non-cirrhotic patients was observed.

Drug metabolism, transport, and drug-drug interactions

Two major metabolites of BI 207127 were identified in humans, CD 6168 (alkene reduction) and BI 208333 (BI 207127-acyl glucuronide), present in plasma at ~ 32 and 23% of BI 207127 related material. CD 6168 is formed in the GI tract, likely by gut bacteria and BI 208333 is formed by UGT1A1, 1A3, 1A7 and 1A8, of which UGT1A1 contribution appears to be predominant.

BI 207127 is a substrate of hepatic uptake transporters OATP1B1, OATP1B3 and OATP2B1. BI 207127 is also a substrate of hepatic and intestinal efflux transporters P-glycoprotein (P-gp) and BCRP.

BI 201335 is a substrate of CYP3A4, which metabolizes it to two hydroxy metabolites M2a and M2b. While together these were found to constitute up to 40% of the dose, M2A and M2b were predominantly found only in feces. The exposure of these metabolites in plasma was very low (<1% of total BI 201335 related material). BI 201335 is a substrate of hepatic uptake transporters OATP1B1, OATP1B3 and OATP2B1.

Proprietary confidential information.
© 2014 Boehringer Ingelheim International GmbH or one or more of its affiliated companies. All rights reserved.
This document may not - in full or in part - be passed on, reproduced, published or otherwise used without prior written permission.

Proprietary confidential information © 2015 Boehringer Ingelheim International GmbH or one or more of its affiliated companies

BI 201335is also a substrate of hepatic and intestinal efflux transporters P-gp and MRP2.

Drug-drug interactions are predicted (based on in vitro DDI data) to be likely if BI 207127 is co-administered with drugs that are substrates of Cytochrome P450 (CYP)1A2, CYP2C8 and P-gp. BI 207127 is also a substrate and an inhibitor of OATP1B1, OATP1B3 and OATP2B1. Until the clinical relevance of these *in vitro* findings are confirmed *in vivo*, patients taking drugs that are CYP1A2 or CYP2C8 substrates with narrow therapeutic indices or sensitive OATP substrates (such as certain statins) are either restricted from concomitant use or recommended for use at the lowest effective doses. The clinical drug interaction study results (1241.18) indicate that inhibition of CYP2C9 and CYP3A4 observed in vitro is not clinically significant.

Proprietary confidential information.
© 2014 Boehringer Ingelheim International GmbH or one or more of its affiliated companies. All rights reserved.
This document may not - in full or in part - be passed on, reproduced, published or otherwise used without prior written permission.

Proprietary confidential information © 2015 Boehringer Ingelheim International GmbH or one or more of its affiliated companies

2. RATIONALE, OBJECTIVES, AND BENEFIT - RISK ASSESSMENT

2.1 RATIONALE FOR PERFORMING THE TRIAL

Patients with compensated liver cirrhosis have been included and successfully treated in SOUND-C2 with BI 207127 in combination with FDV and RBV. Trial 1241.30 will assess this combination treatment in patients with decompensated cirrhosis, a patient population with a high unmet medical need and no available antiviral treatment options, as PegIFN is contraindicated. This study will aim to define an interferon-free regimen that may offer an antiviral treatment option for patients chronically infected HCV GT1b with moderate hepatic impairment (CPB).

2.2 TRIAL OBJECTIVES

The objective of Cohort A is to evaluate the safety and pharmacokinetic (PK) profile of BI 207127 (potentially two doses) in combination with 120 mg once daily (q.d.) FDV and weight-based RBV in a small group of patients with moderate hepatic impairment (CPB) compared to patients with mild hepatic impairment (CPA)~~ to define the BI 207127 dose to be used in Cohort B~~.

~~The objective of Cohort B is to assess efficacy, safety, and pharmacokinetics of 24-week treatment of the BI 207127 dose selected in Cohort A in combination with 120 mg once daily (q.d.) FDV and weight - based RBV in a larger group of chronically infected HCV GT1b patients with moderate hepatic impairment (CPB).~~

2.3 BENEFIT - RISK ASSESSMENT

Patients participating in this study have a high unmet medical need with their only available treatment option for decompensated cirrhosis being liver transplantation. Once cirrhosis has developed, mortality is approximately 10% over the subsequent 10 years. Clinical hepatic decompensation occurs in 30% of patients with cirrhosis and 5-year survival declines to about 50% (P97-2059).

The inclusion of treatment naïve patients is supported by data from the SOUND-C2 trial, the largest phase 2 trials with all-oral HCV treatment performed to date. Efficacy and safety in patients with compensated liver cirrhosis was studied in SOUND-C2 and the BI 207127 600 mg b.i.d. dose regimen demonstrated the best benefit/risk ratio in this population. Among GT1b patients with cirrhosis randomised to the b.i.d. group, the SVR12 rate was 80% and the rate of AEs leading to discontinuation of treatment was similar to the overall patients in same arm (approximately 8%). Most AEs were of mild intensity; the most common AEs were rash, photosensitivity reactions, asthenia, fatigue, jaundice (isolated indirect hyperbilirubinemia), nausea, vomiting and diarrhoea.

Many patients with progressive liver cirrhosis have been exposed to PegIFN/RBV in the past. Thus, to secure sufficient enrolment in this important study for a high-medical need

Proprietary confidential information.
© 2014 Boehringer Ingelheim International GmbH or one or more of its affiliated companies. All rights reserved.
This document may not - in full or in part - be passed on, reproduced, published or otherwise used without prior written permission.

Proprietary confidential information © 2015 Boehringer Ingelheim International GmbH or one or more of its affiliated companies

population treatment experienced patients (prior relapse, interferon intolerant, and [allowed in Cohort A only] prior partial response) will also be included (cf. Section 3.3.2 for definitions).

This trial will include a PK sub-study for Cohort B to further assess the BI 207127 b.i.d. dose selected in Cohort A in a larger group of CPB patients. Refer to Section 4.1.3 for selection of dose rationale. Rash and photosensitivity reaction were reported in 20-30% of patients. Rash was commonly reported as maculo-papular skin reaction on the trunk and extremities, while photosensitivity reactions were described as exaggerated sunburns in hands, face or any other sun-exposed skin area. Among patients who received 600 mg b.i.d. BI 207127 in combination with FDV and RBV, all skin reactions were mild. There were no moderate, severe, or serious skin reactions or discontinuation of treatment due to skin events reported. Serious rash and photosensitivity reactions were only reported with higher BI 207127 dose (600 mg tid) and none of the cases were considered life-threatening. There were no cases of Stevens-Johnson, erythema multiforme, toxic epidermal necrolysis (TEN) or drug rash with eosinophilia and systemic symptoms (DRESS) reported in any of the BI 207127-FDV combination trials to date. However, DRESS has been reported in FDV program in combination with PegIFN/RBV at 120 and 240 mg QD doses. Rash and photosensitivity reactions will be carefully monitored and managed with a detailed dermatologic management plan (cf. Appendix 10.2 and ISF). The dermatologic plan includes precautionary measures to restrict exposure to sun and artificial ultraviolet (UV) light and instructions to decrease the dose of BI 207127 in specific cases. Every skin reaction fulfilling the criteria as protocol-defined significant AEs will be reported in an expedited manner similar to SAEs and will be subject to review by a dermatological adjudication committee (cf. Sections 3.1.1.2 and 5.2.2).

GI events such as nausea, vomiting and diarrhoea were reported in BI 207127-FDV combination trials. The highest risk of vomiting in previous trials was observed during the first 2 days of treatment (17% cumulative risk of vomiting during the first 2 days) (U11-3471-01). There was an association between the high BI 207127 exposure at Day 1 and the risk of GI events (U11-3472-01). This may have been due to the administration of an induction dose of BI 207127 (1200 mg, 2 times the individual dose) on the morning of first day of dosing. Since there was no virologic benefit associated with the induction dose (no association between BI 207127 exposure and the initial HCV RNA decay kinetics), this induction dose will not be used in this trial. As such, the risk of vomiting during the first 2 days of treatment in this trial may be lower than in previous trials. PegIFN-free regimens of BI 207127 in Trial 1241.21 Parts 1 and 2 did not cause any relevant changes from baseline in laboratory parameters, except for a continuous drop in alanine aminotransferase (ALT) in all patients, a decrease in haemoglobin (Hb, consistent with RBV treatment) and increase in total bilirubin due primarily to increases in the unconjugated fraction (a known clinically benign effect of FDV treatment). Up to 25% of patients experienced jaundice due to increases in unconjugated bilirubin without concomitant increases in liver function tests (LFTs) or any sign of liver toxicity.

As of 24 October 2013, DAIDS Grade IV neutropenia (absolute neutrophil counts $\leq$ 500 cells/mm^3) has occurred in 4 patients treated with BI 207127-FDV-RBV combination therapy over approximately 1400 patients treated with this regimen. The events occurred at or beyond Week 12 of treatment which is atypical for drug-induced neutropenia. Three out of 4 patients fully recovered after treatment discontinuation. The remaining patient recovered after

Proprietary confidential information.
© 2014 Boehringer Ingelheim International GmbH or one or more of its affiliated companies. All rights reserved.
This document may not - in full or in part - be passed on, reproduced, published or otherwise used without prior written permission.

Proprietary confidential information © 2015 Boehringer Ingelheim International GmbH or one or more of its affiliated companies

5 days of treatment interruption and was re-challenged with BI 207127-FDV-RBV without recurrence of neutropenia. Mean absolute neutrophil count in patients during the course of treatment remains similar to the levels observed at baseline in the completed and ongoing Phase 3 trials with BI 207127+FDV+RBV. At this stage, it is unclear whether these events are related to the study drugs, underlying disease (hepatitis) or other conditions.

Of note, only approximately 10% of patients experienced anaemia (defined as Hb > 10g/dL) receiving the oral combination in SOUND-C2 compared to 36% of receiving telaprevir with PegIFN/RBV (R12-0262) or 49% of receiving boceprevir with PegIFN/RBV (R12-0263). This may be explained by the lack of PegIFN and its myelosuppressive effect which enhances the negative effect of RBV in the regimen.

The evaluation of the ECGs in one phase 1b/2 trial in Japan (trial 1241.25) suggested an increase in the QTc of 10 to 20 ms compared to baseline for patients receiving a combination of the three active drugs. However, the trial was not designed to evaluate QT effects, but rather collected the ECG data in a routine manner without controlling for factors known to affect the QT interval and introducing a potential bias in the assessment. Of note, there were no patients in the trial 1241.25 with abnormal QTc interval defined according to current standards as >450 ms for males and >470 ms for females. Based on an extensive phase 1-3 clinical database of subjects receiving faldaprevir (approximately 3355 subjects) and BI 207127 (approximately 600 subjects), there have been no reports of unexplained serious arrhythmias, QT prolongations, torsades de pointes or sudden deaths. A thorough QTc trial with faldaprevir only (trial 1220.16) demonstrated no QT prolongation in a manner that suggested any clinically relevant effects on cardiac repolarization as per the FDA and EMA guidance. Another thorough QTc study with faldaprevir and BI207127 combination therapy is currently planned to further evaluate any potential for QTc prolongation.

Based on the current understanding that cirrhotic patients require longer treatment durations and the lack of substantial clinical data in cirrhotic patients treated for 16 weeks in phase 2, patients with compensated cirrhosis will receive treatment for 24 weeks.

A potential risk with a PegIFN-free regimen is the selection of mutations conferring resistance to both compound classes which may limit future treatments options. However, it is currently not known if HCV resistant variants are archived and persist. Therefore it might be possible to retreat patients that had selected for resistance mutation on a given DAA(s) with the same regiment once the variants are no longer detectable. Additionally, major resistant variants associated with the use of BI 207127, such as P495L, would only confer resistance to another thumb site-1 nonnucleoside NS5B inhibitor. There are currently a number of DAAs with different MOA in development (including: thumb site-2 NS5B inhibitors, NS5A inhibitors, nuclotide NS5B inhibitors, cyclophlin inhibitors, etc.). Future treatment options will be expanded once some of these agents become available to patients in the near future. To maximize the chance to achieve SVR and reduce the chance of emergence of resistance only GT1b patients (which have the highest rate of response to this regimen) will be included.

~~Patients with decompensated cirrhosis randomised to placebo will not receive benefit from participating in the trial. However, the knowledge gained from this treatment group will be invaluable in understanding the emergence of AEs in decompensated cirrhotic patients during~~

Proprietary confidential information.
© 2014 Boehringer Ingelheim International GmbH or one or more of its affiliated companies. All rights reserved.
This document may not - in full or in part - be passed on, reproduced, published or otherwise used without prior written permission.

Proprietary confidential information © 2015 Boehringer Ingelheim International GmbH or one or more of its affiliated companies

~~placebo treatment, and thus understanding of the safety profile of the tested regimen in this population. These patients currently have no HCV treatment options and participation in this trial will not compromise future treatment options for these patients. Patients randomised to receive placebo will be given the option of receiving Arm 5 treatment after completion of 24 weeks, if still eligible for treatment (cf. Section 3.1). In addition, an independent Data Monitoring Committee (DMC) will be established (cf. Section 3.1.1.1) and all patients will be frequently monitored. If the risk-benefit ratio changes upon favourable on-treatment clinical data in the active treatment arm 5 of Cohort B, the DMC may recommend that placebo arm patients are offered the option of receiving active treatment prior to completion of 24 weeks of placebo.~~

Other risks to the patients are the risks inherent to any clinical trial, such as unexpected adverse clinical or laboratory events. Patients will be closely monitored for AEs and rebounds of plasma HCV RNA.

Although rare, a potential for drug-induced liver injury (DILI) is under constant surveillance by sponsors and regulators. Therefore, this study requires timely detection, evaluation, and follow-up of laboratory abnormalities of selected liver laboratory parameters to ensure patients' safety.

Given the good safety profile of this PegIFN-free combination treatment, coupled with rapid and potent antiviral activity observed so far, the sponsor assesses benefit risk relationship in this study as positive, and the knowledge gained will outweigh the potential risk for this patient population with an unmet medical need.

Proprietary confidential information.
© 2014 Boehringer Ingelheim International GmbH or one or more of its affiliated companies. All rights reserved.
This document may not - in full or in part - be passed on, reproduced, published or otherwise used without prior written permission.

Proprietary confidential information © 2015 Boehringer Ingelheim International GmbH or one or more of its affiliated companies

3. DESCRIPTION OF DESIGN AND TRIAL POPULATION

3.1 OVERALL TRIAL DESIGN AND PLAN

This is a multi-centre ~~and multi-staged~~ trial.~~ Interim data from Cohort A (open label) will be used to select the BI 207127 dose to be assessed in Cohort B (double-blind, and placebo controlled, randomised with a 1:3 ratio for Arm 4 and Arm 5) CPB patients.~~

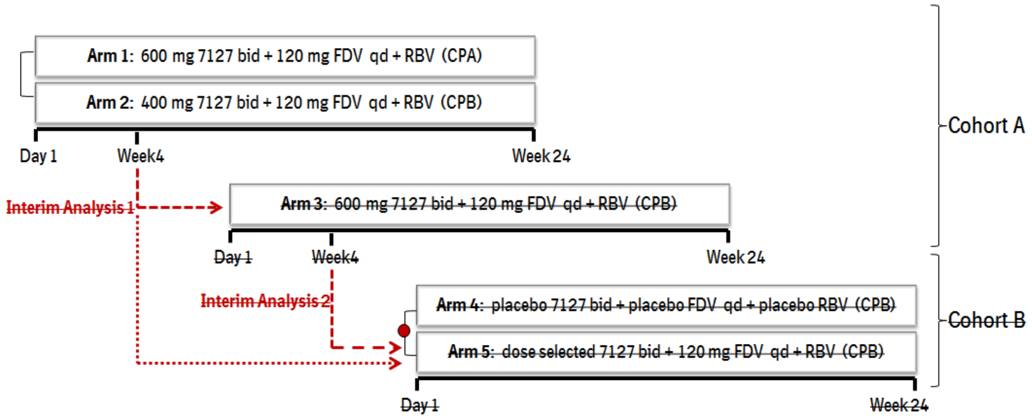

Figure 3.1: 1 Trial design

In total, approximately ~~165~~ 30 patients are planned for inclusion in this trial. Refer to Table 3.1: 1 for details of HCV sub-type and Child Pugh score for each treatment arm.

Table 3.1: 1 Treatment arm descriptions

		HCV sub-type	Child Pugh Score	Blinding	n	Treatment*
**Cohort A**	**Arm 1**	GT1b	CPA	open label	15	600 mg BI 207127 b.i.d. + 120 mg FDV q.d. + RBV
	**Arm 2**	GT1b	CPB	open label	15	400 mg BI 207127 b.i.d. + 120 mg FDV q.d. + RBV
	~~**Arm 3**~~	~~GT1b~~	~~CPB~~	~~open label~~	~~15~~	~~600 mg BI 207127 b.i.d. + 120 mg FDV q.d. + RBV~~
~~**Cohort B**~~	~~**Arm 4**~~	~~GT1b~~	~~CPB~~	~~double-blind~~	~~30~~	~~placebo BI 207127 b.i.d. + placebo FDV q.d. + placebo RBV~~
	~~**Arm 5**~~	~~GT1b~~	~~CPB~~	~~double-blind~~	~~90~~	~~dose selected of BI 207127 b.i.d. + 120 mg FDV q.d. + RBV~~

* FDV loading dose of 240 mg total on Day 1

Patients are included in the study once they have signed the informed consent. Patients suitable after screening will be eligible to participate in the 24 week treatment period and,

Proprietary confidential information.
© 2014 Boehringer Ingelheim International GmbH or one or more of its affiliated companies. All rights reserved.
This document may not - in full or in part - be passed on, reproduced, published or otherwise used without prior written permission.

Proprietary confidential information © 2015 Boehringer Ingelheim International GmbH or one or more of its affiliated companies

according to Figure 3.1: 2 will be assigned to an arm open to enrolment depending on the current screening stage of the trial.

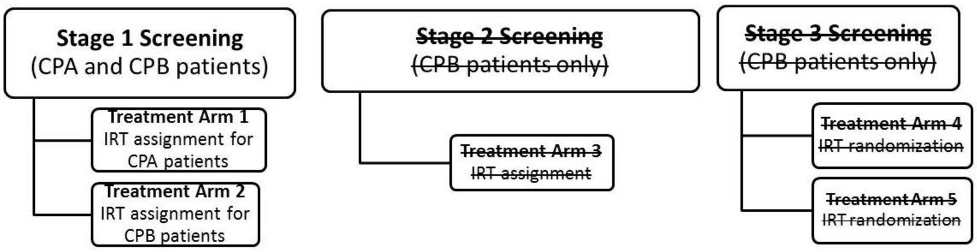

Figure 3.1: 2 Assignment of treatment arm by trial screening stage

~~Patients randomised to Arm 4 (placebo) will be given the option of receiving active treatment (as in Arm 5) after completion of their individual 24 week placebo treatment, if still eligible for treatment, or sooner, if results from ongoing trial as evaluated by the DMC support this (cf. Section 3.1.1.1 and ISF).~~

~~Forty eight patients in Cohort B will be assigned via Interactive Response Tool (IRT) to participate in a PK sub-study (cf. Section 5.5).~~

"On-treatment" AEs are those AEs that begin during treatment with the trial medications or within 28 days of the End of Treatment (EOT) visit. For more details on safety assessments in this trial, cf. Section 5.2 and Section 7.3.3.

Upon EOT visit patients will complete an additional ~~96~~ 12 weeks follow-up (FU) period. Individual patient participation is concluded when the patient has completed the last planned visit.

The end of the trial is defined as "last patient out", i.e. last scheduled visit completed by last patient.

3.1.1 Administrative structure of the trial

The trial is sponsored by Boehringer Ingelheim.

Boehringer Ingelheim (BI) will appoint a Trial Clinical Monitor (TCM), responsible for coordinating the activities required in order to manage the trial in accordance with applicable regulations and internal standard operating procedures (SOPs), directing the clinical trial team in the preparation, conduct, and reporting of the trial, order the materials as needed for the trial, ensures appropriate training and information of Local Clinical Monitors (CML), Clinical Research Associates (CRAs), and investigators of participating countries.

Data Management and Statistical evaluation will be performed by BI according to BI SOPs. For these activities, a Trial Data Manager and a Trial Statistician will be appointed.

Proprietary confidential information.
© 2014 Boehringer Ingelheim International GmbH or one or more of its affiliated companies. All rights reserved.
This document may not - in full or in part - be passed on, reproduced, published or otherwise used without prior written permission.

Proprietary confidential information © 2015 Boehringer Ingelheim International GmbH or one or more of its affiliated companies

Tasks and functions assigned in order to organise, manage, and evaluate the trial will be defined according to BI SOPs. A list of responsible persons will be given in the Clinical Trial Master File (CTMF) document.

The organisation of the trial in the participating countries will be done by the respective local BI-organisation (Operative Unit (OPU)) or by a Contract Research Organisation (CRO) with which the responsibilities and tasks will have been agreed and a written contract filed before initiation of the clinical trial. In each OPU participating in this study, a CML will be appointed responsible for coordinating the activities required in order to manage the trial in accordance with applicable regulations and internal SOPs in the countries covered by the respective BI OPU.

A Co-ordinating Investigator will be nominated to coordinate investigators at different sites participating in this multicentre trial. Tasks and responsibilities for the Co-ordinating Investigators will be defined in a contract filed before initiation of the trial.

Documents on participating (Principal) investigators and other important study personnel, especially their curricula vitae, will be filed in the CTMF.

Details of the trial supplies including responsible institutions are given in Section 4 of this protocol.

The ISF will be maintained at the sites as required by local regulation and BI-SOP. A copy of the ISF documents will be kept as an electronic CTMF document according to BI SOPs.

A central laboratory service and IRT will be used in this trial.

3.1.1.1 Data Monitoring Committee (DMC)

The study will have a DMC independent from the sponsor. The purpose of the DMC is to ensure that the welfare of patients participating in this trial is maintained by:

- Monitoring the trial for possible untoward harmful effects or unexpected frequency of adverse safety events of study drugs;
- Assessing whether the goals of the trial are unlikely to be achieved, based on emerging data.

The DMC will evaluate the unblinded accrued patient data in order to recommend whether the trial or program should continue, be modified or stopped for safety concerns or ethical reasons. The DMC will review pertinent trial data, including deaths, serious adverse events (SAEs) and AEs, laboratory data and efficacy markers (HCV RNA). Unless specified differently in the DMC charter, it is expected that the first DMC meeting will occur approximately 2 months after the first patient has entered the trial and then every 3-4 months thereafter, with more frequent reviews as deemed necessary by the DMC or the sponsor. ~~The DMC will review Cohort A Week 4 data prior to the start of Cohort B.~~ All materials provided to the DMC are confidential. The tasks and responsibilities of the DMC will be filed in a charter before initiation of the trial and will contain written operating procedures.

Proprietary confidential information.
© 2014 Boehringer Ingelheim International GmbH or one or more of its affiliated companies. All rights reserved.
This document may not - in full or in part - be passed on, reproduced, published or otherwise used without prior written permission.

Proprietary confidential information © 2015 Boehringer Ingelheim International GmbH or one or more of its affiliated companies

The DMC will maintain written records of all its meetings. ~~Sponsor will remain blinded to these ongoing data reviews.~~

3.1.1.2 Rash and Photosensitivity Adjudication Committee (RPAC)

The study will include an independent committee of dermatology experts responsible for evaluation of protocol-defined significant skin AEs (cf. Section 5.2.2.1). The tasks and responsibilities for this committee will be established by contract before initiation of the trial and will contain written operating procedures.

~~3.1.1.3 Internal independent data review committee~~

~~The study will include a committee of sponsor members that are independent from the trial team. This committee will evaluate interim analysis data, including PK data, to select the BI 207127 dose for Cohort B. Refer to Section 7.3.4 for interim analysis plan and dose selection criteria.~~

~~The first interim analysis will be performed on Arm 1 and Arm 2 data up to Week 4 for submission to the committee. The BI 207127 dose for Cohort B will be selected or it will be determined that it is necessary to conduct Arm 3. If Arm 3 is needed, a second interim analysis will be performed on Arm 1 and Arm 3 data up to Week 4 for submission to the committee for Cohort B dose evaluation. Thus, randomisation in Cohort B will begin based on the Week 4 interim data of Cohort A.~~

~~The tasks and responsibilities for the Internal Independent Data Review Committee will be established before initiation of the trial and will contain written operating procedures.~~

3.2 DISCUSSION OF TRIAL DESIGN, INCLUDING THE CHOICE OF CONTROL GROUPS

The CPB patient population was selected because these patients, despite having the highest urgency for treatment, currently have no HCV treatment options and therefore have a high unmet medical need. Patients with poorly controlled ascites are excluded due to common fluctuations in ascites i.e. the distribution volume which could interfere with the PK analysis of both compounds.

The CPA control group was chosen because there is limited PK data in CPA patients in the phase II program for BI 207127.

Due to the low SVR rate seen in phase 2 studies in GT1a patients (cf. Table 1.2: 1), these patients are excluded from the program until a more effective treatment regimen has been identified.

~~The study is double-blind in order to eliminate the reporter/observer bias for the assessment of safety and of the treatment duration on efficacy between treatment Arm 4 and Arm 5.~~

~~The Arm 4 placebo group will allow comparison of safety between active treatment and placebo. It will be informative to assist in the identification of AEs related to active triple~~

Proprietary confidential information.
© 2014 Boehringer Ingelheim International GmbH or one or more of its affiliated companies. All rights reserved.
This document may not - in full or in part - be passed on, reproduced, published or otherwise used without prior written permission.

Proprietary confidential information © 2015 Boehringer Ingelheim International GmbH or one or more of its affiliated companies

therapy versus symptoms associated with the underlying diseases and co-morbidities in CPB patients. The CPB placebo group is ethically acceptable since this population currently does not have an antiviral treatment option and their participation will be closely monitored by the DMC. The duration of the 24 week placebo treatment may be potentially shortened and active treatment offered, if results from ongoing trial change the risk benefit ratio as evaluated by the DMC.

The smaller sample size of the PBO group was selected as a compromise between the expectation of spontaneous symptoms and (S)AE of patients due to underlying severe liver disease and the withholding of active treatment from such patients with high treatment need. It is recognised that double-blinding of such a treatment group will be challenging because of multiple laboratory test changes, specifically viral load, bilirubin, liver enzymes and hematologic changes that are related to the administration of active triple therapy vs. no such changes in patients in Arm 4. Nevertheless, extensive efforts will be made to overcome these difficulties through blinding selected laboratory tests for Arm 4 and Arm 5 to investigator, patient and sponsor (cf. Section 4.1.5.1).

PegIFN is not used in the trial and therefore, contraindications to use of PegIFN do not apply as exclusions in the eligibility criteria. Patients with retinopathies, history of psychiatric diseases, autoimmune diseases, thrombocytopenia and other conditions that contraindicate the use of PegIFN, may be entered in the study as long as their co-morbidities are considered clinically stable and with appropriate treatment, if necessary. This will ensure that the trial population is as representative as possible of the patient population seen in clinical practice.

Extended 96 **12**-week FU period will allow assessment of the persistence of the virological response, assessment of the progression of the liver disease and resistance profile of HCV over time. In case of re-detection of HCV RNA during the FU period, assessment will be made whether this represents a relapse or a subsequent re-infection.

3.3 SELECTION OF TRIAL POPULATION

A sufficient number of patients will be screened for the trial to ensure that approximately 165 **30** patients are randomised to trial treatment (15 patients in each treatment Arm 1, Arm 2, and Arm 3; 30 patients in Arm 4, and 90 patients in Arm 5).

Demographic information, including patient gender, race and ethnicity, will be collected at screening.

Treatment Arm 1, **and** Arm 2 and Arm 3 will be stratified by gender with a minimum of 8 females per arm (cf. Section 7.5).

Screening of patients for this trial is competitive, i.e. screening for the trial will stop at all centres when such a number of patients has been screened that it is anticipated that a sufficient number of patients will be randomised to trial treatment. This process of competitive screening will be carried out independently for each screening stage (cf. Figure 3.1: 2).

Proprietary confidential information.
© 2014 Boehringer Ingelheim International GmbH or one or more of its affiliated companies. All rights reserved.
This document may not - in full or in part - be passed on, reproduced, published or otherwise used without prior written permission.

Proprietary confidential information © 2015 Boehringer Ingelheim International GmbH or one or more of its affiliated companies

For Cohort A, patient replacement will be permitted (i.e. those patients who were assigned to a treatment group but did not receive any study drug will be replaced and those that discontinued study drug prior to Week 4 may be replaced as needed to ensure sufficient PK sample size).

~~For Cohort B, patients assigned to a treatment group who discontinue from the trial after start of treatment will not be replaced (i.e. those patients who were assigned to a treatment group but did not receive any study drug will be replaced to ensure that patients will receive study drug in each treatment group).~~

Re-screening of patients will not be permitted.

A log of all patients included into the study (i.e. having given informed consent) will be maintained in the ISF at the investigational site irrespective of whether they have been treated with investigational drug or not.

3.3.1 Main diagnosis for study entry

This study will be performed in treatment naïve and experienced patients (see inclusion criteria 4 for definitions) with chronic HCV infection of GT1, sub-GT1b virus only.

3.3.2 Inclusion criteria

1. Chronic HCV infection, diagnosed by positive anti-HCV antibodies and detected HCV RNA at screening in addition to at least one of the following:
    a) positive anti-HCV antibodies or detected HCV RNA at least 6 months prior to screening
    b) liver biopsy consistent with chronic HCV infection
    c) history of elevated ALT levels at least 6 months prior to screening
2. HCV infection, confirmed by genotypic testing at screening, of GT1, sub-GT1b virus.
3. HCV viral load ≥ 1,000 IU/ml at screening
4. Previous treatment status must be one of the following:
    a) Treatment naïve: defined as patients who have never been previously treated with any interferon, with an investigational/approved DAA or any other HCV treatment regimen.
    b) Treatment experienced to an approved PegIFN/RBV regimen with prior relapse: defined as HCV RNA level > 25 IU/mL during the post-treatment period in patients who had plasma HCV RNA level undetected at end of all treatment
    c) Treatment experienced interferon intolerant: defined as patients who had to stop a previous PegIFN/RBV regimen before week 12 of treatment due to safety or tolerability issues.

Proprietary confidential information.
© 2014 Boehringer Ingelheim International GmbH or one or more of its affiliated companies. All rights reserved.
This document may not - in full or in part - be passed on, reproduced, published or otherwise used without prior written permission.

Proprietary confidential information © 2015 Boehringer Ingelheim International GmbH or one or more of its affiliated companies

d) ~~(Allowed in Cohort A only)~~ Treatment experienced to an approved PegIFN/RBV regimen with prior partial response: defined as HCV RNA level > 25 IU/mL at end of treatment in patients who had HCV RNA drop by ≥2 log10 from baseline at Week 12.

e) Treatment experienced to interferon not as part of an approved PegIFN/RBV regimen.

5. Liver cirrhosis defined as at least one of the following:
   f) Metavir Grade =4 or Ishak Grade ≥5 on liver biopsy
   g) liver stiffness of ≥13 kPa on fibroscan
6. No evidence of liver cancer in an appropriate imaging study (e.g., ultrasound, CT scan, or magnetic resonance imaging [MRI]) within last 3 months prior to randomisation.
7. No active variceal bleeding or large varices in an upper GI endoscopy, as part of standard of care in patients with liver cirrhosis, performed within 12 months prior to randomization. Note: Patients may be included if they have a history of variceal bleeding or a history of large varices, if properly treated and monitored.
8. Age 18 to 75 years (inclusive)
9. Female patients with a negative urine pregnancy test (dipstick) at Visit 2 prior to randomization:
   a) with documented hysterectomy,
   b) who have had both ovaries removed,
   c) with documented tubal ligation,
   d) who are post-menopausal with last menstrual period at least 12 months prior to screening, or
   e) of childbearing potential with a negative serum pregnancy test at screening and Day 1, who, if sexually active, agree to use two non-hormonal methods of birth control from the date of screening until 7 months after the last dose of RBV. Patients must agree not to breast-feed at any time from the date of screening until 7 months after the last dose of RBV. Accepted methods of contraception in the study include diaphragm with spermicidal substance, cervical caps, intrauterine devices and condoms.

      Note: Systemic hormonal contraceptives may not be as effective in women taking BI 207127/FDV combination therapy and are not accepted methods of contraception in the study.

   Male patients:
   a) who are documented to be sterile, or
   b) who are without pregnant female partner(s) and consistently and correctly use a condom while their female partner(s) (if of child-bearing potential) use one of the appropriate medically accepted methods of birth control from the date of screening until 7 months after the last dose of RBV. It is in the responsibility of the male patient to ensure that his partner(s) is not pregnant prior to screening into the study or becomes pregnant during the treatment and the observation phase. Female partners of childbearing potential should

Proprietary confidential information.
© 2014 Boehringer Ingelheim International GmbH or one or more of its affiliated companies. All rights reserved.
This document may not - in full or in part - be passed on, reproduced, published or otherwise used without prior written permission.

Proprietary confidential information © 2015 Boehringer Ingelheim International GmbH or one or more of its affiliated companies

perform monthly pregnancy tests from the date of screening until 7 months after the last dose of RBV (tests will be provided by the sponsor).

10. Signed informed consent form prior to trial participation

3.3.3 Exclusion criteria

1. HCV infection of mixed genotype (1/2, 1/3, and 1/4) or mixed sub-GT1a/1b or undefined diagnosed by genotypic testing at screening
2. Liver disease due to causes other than chronic HCV infection which may include but is not limited to hemochromatosis, Wilson's disease, or autoimmune liver diseases.
3. HIV infection
4. Hepatitis B virus infection based on presence of HBs-Ag
5. Confirmed or suspected active malignancy or history of malignancy within the last 5 years prior to screening (with an exception of appropriately treated basal cell carcinoma of the skin or in situ carcinoma of the uterine cervix).
6. History of chronic alcohol abuse or illicit drug use other than cannabis within 12 months prior to randomisation, in the opinion of the investigator.
7. Patient is not willing to comply with the restriction of no alcohol consumption.
8. Patient is not willing to comply with the precautionary measures to prevent photosensitivity (avoid excessive sun exposure and use sun block on a daily basis).
9. A condition that is insufficiently diagnosed, treated or clinically unstable which, in the opinion of investigator, may put the patient at risk because of participation in this study, influence the results of this study, or limit the patient's ability to participate in this study, including but is not limited to severe chronic obstructive pulmonary disease and uncontrolled psychiatric disease.
10. Total bilirubin > 3 mg/dL with ratio of direct/indirect > 1
11. Serum albumin < 2.4 g/dL
12. Prothrombin time International Normalised Ratio (INR) > 2.3
13. Active poorly controlled ascites
14. Encephalopathy graded as other than minimal (graded > 1 with West Haven Criteria)
15. Clinical evidence of unstable cardiovascular disease which may further decompensate due to anemia, including unstable angina, recent myocardial infarction, cardiomyopathy, congestive heart failure, uncontrolled hypertension or significant arrhythmia.
16. Red blood cell (RBC) disorders, including thalassemia major, sickle cell anemia or G6PD deficit. Patients with traits or minor diseases (e.g. sickle cell trait or thalassemia minor) may be enrolled if the disease did not result in anemia according to the investigator's clinical judgement.
17. Body weight < 40 or > 125 kg
18. Usage of any investigational drugs within 28 days prior to randomisation, or planned usage of an investigational drug during the course of this study
19. Received concomitant immunomodulatory treatment within 28 days prior to screening

Proprietary confidential information.
© 2014 Boehringer Ingelheim International GmbH or one or more of its affiliated companies. All rights reserved.
This document may not - in full or in part - be passed on, reproduced, published or otherwise used without prior written permission.

Proprietary confidential information © 2015 Boehringer Ingelheim International GmbH or one or more of its affiliated companies

20. Received silymarin (milk thistle), glycyrrhizin, Sho-saiko-to (SST) or any medication listed in a restricted medication list provided in ISF within 28 days prior to randomisation, with the exception of parenteral analgesics used during liver biopsy procedure.
21. Known hypersensitivity to any ingredient of the study drugs
22. Hb < 11 g/dL for women and men
23. Absolute neutrophil count < 1,000 cells/mm^3
24. Creatinine clearance ≤ 50 ml/min
26. MELD score > 20
27. Platelet count < 40,000 cells/mm^3
28. Patients who have been previously treated with an investigational or approved DAA.
29. Patients classified as Child Pugh C (score 10-15 points)

3.3.4 Removal of patients from therapy or assessments

3.3.4.1 Removal of individual patients

Patients have the right to withdraw from the study at any time without the need to justify the decision. The investigator has the right to remove patients from the study for non-adherence. Furthermore, patients' treatment should be discontinued early due to lack of viral response or other reasons (as described below). The sponsor reserves the right to terminate a patient from the trial for non-adherence. It is understood that an excessive rate of withdrawals can render the study results uninterpretable; therefore unnecessary withdrawal of patients should be avoided. Patients who discontinue their treatment early will be followed throughout the course of the study and undergo all study required procedures as defined in Section 6.2.3.

If a patient is suspected to be, or becomes, pregnant during treatment (including rescue treatment), the patient must inform the investigator immediately and stop taking all study drugs. For the reporting, follow up and documentation of pregnancy cases refer to Section 5.2.2.2. These patients will be followed throughout the course of the study as defined in Section 6.2.3. ~~If a female partner of a male patient becomes pregnant during treatment (including rescue treatment), the patient should inform the investigator immediately and investigator must inform the sponsor.~~ **If a male subject reports the pregnancy of his female partner to the investigator, the pregnancy must be reported to the sponsor AND followed up. Part B of the pregnancy monitoring form should be submitted upon outcome of the pregnancy.** The decision on whether to discontinue the patient treatment must be made by physician investigator based on the local RBV label and his clinical judgment. ~~The details of female partner's pregnancy will neither be tracked nor recorded in the trial documentation.~~

Patients will discontinue their assigned treatment earlier than planned due to lack of on-treatment viral response:

a) If they have virologic breakthrough defined as:

Proprietary confidential information.
© 2014 Boehringer Ingelheim International GmbH or one or more of its affiliated companies. All rights reserved.
This document may not - in full or in part - be passed on, reproduced, published or otherwise used without prior written permission.

Proprietary confidential information © 2015 Boehringer Ingelheim International GmbH or one or more of its affiliated companies

1) Increase of ≥1 log10 in plasma HCV RNA from a quantifiable nadir* confirmed by a second consecutive plasma HCV RNA measurement as soon as possible and within 2 weeks time; or,

2) HCV RNA ≥25 IU/mL after previous plasma HCV RNA <25 IU/mL, which must be confirmed by a second consecutive plasma HCV RNA measurement as soon as possible and within 2 weeks time.

    - If the retest (second consecutive plasma HCV RNA) is ≥ 500 IU/mL the patient will discontinue the treatment.
    - If the retest is lower than 500 IU/mL, and more than the first HCV RNA measurement of HCV RNA ≥25 IU/mL the patient will discontinue the treatment.
    - If the retest is lower than 500 IU/mL, and less than the first HCV RNA measurement of HCV RNA ≥25 IU/mL, a third HCV RNA testing should be performed and must be ≥25 IU/mL for the patient to discontinue the treatment.

b) If they never achieve plasma HCV RNA <25 IU/mL and have plasma HCV RNA ≥25 IU/mL at any time from Week 12 of active treatment onwards (Note: Patients with virologic breakthrough as defined above are not included)

* HCV RNA nadir refers to the lowest HCV RNA measurement while on treatment.

These rules will be used only while patients are on-treatment (i.e. in order to stop treatment due to lack on-treatment viral response). Once patients stop treatment, the retest requirements as described in Section 5.1.1 for SVR endpoints will apply.

Patients may discontinue their assigned treatment earlier than planned due to reasons other than lack of viral response, as listed below and based on the clinical judgement of investigator:

- Development of a toxicity or AE which warrants drug discontinuation.
- Development of any potentially life-threatening toxicity
    - Development of adverse skin reactions concomitant with systemic symptoms such as persistent fever (≥ 38.5°C lasting more than 24 hours), lymphadenopathy and/or involvement of mucous membranes and internal organs.
- Patient has to take any concomitant drug interfering with the study medications (list provided in ISF: this should be discussed with the BI Clinical Monitor prior to discontinuation).
- Patient is no longer able to participate for other medical reasons (e.g., surgery, pregnancy or medical contraindication to BI 207127, FDV or RBV).
- Concurrent increase in total bilirubin and ALT or aspartate aminotransferase (AST): bilirubin >5x upper limit normal (ULN) with a ratio of direct/indirect bilirubin >1 and

Proprietary confidential information.
© 2014 Boehringer Ingelheim International GmbH or one or more of its affiliated companies. All rights reserved.
This document may not - in full or in part - be passed on, reproduced, published or otherwise used without prior written permission.

Proprietary confidential information © 2015 Boehringer Ingelheim International GmbH or one or more of its affiliated companies

ALT or AST >2x baseline with an absolute increase of at least 200 IU/L over the ALT or AST value at baseline.

- Concurrent increase in total bilirubin and prolongation of INR: bilirubin >5x ULN with a ratio of direct/indirect bilirubin >1 and INR >2.3.
- Concurrent increase in ALT or AST and prolongation of INR: ALT or AST >2x baseline with an absolute increase of at least 200 IU/L over the ALT or AST value at baseline, and INR >2.3.

Clinical judgment needs to be exercised for stopping patients' treatment due to life-threatening toxicities. With regard to an occurrence of a potentially life-threatening neutropenia (DAIDS grade IV - absolute neutrophil count ≤ 500 cells/mm^3), patients must stop all study drugs immediately. Other clinical conditions and concomitant medications should be considered and thoroughly evaluated in an urgent manner and appropriate clinical therapies provided. Consultation with a hematologist may be warranted and should be documented.

3.3.4.2 Discontinuation of the trial by the sponsor

Boehringer Ingelheim reserves the right to discontinue the trial overall or at a particular trial site at any time for the following reasons:

1. Failure to meet expected enrolment goals overall or at a particular trial site,
2. Emergence of any efficacy/safety information that could significantly affect continuation of the trial,
3. Violation of Good Clinical Practice (GCP), the clinical trial protocol (CTP), or the contract by a trial site or investigator, disturbing the appropriate conduct of the trial.

The investigator / the trial site will be reimbursed for reasonable expenses incurred in case of trial termination (except in case of the third reason).

Proprietary confidential information.
© 2014 Boehringer Ingelheim International GmbH or one or more of its affiliated companies. All rights reserved.
This document may not - in full or in part - be passed on, reproduced, published or otherwise used without prior written permission.

Proprietary confidential information © 2015 Boehringer Ingelheim International GmbH or one or more of its affiliated companies

4. TREATMENTS

4.1 TREATMENTS TO BE ADMINISTERED

4.1.1 Identity of BI investigational products and comparator products

The following tables summarise the information about the investigational, non-investigational medicinal products, and comparator products as used in this trial.

Table 4.1.1: 1 Characteristics of the test products and comparator products

Substance	Pharma-ceutical form	Source	Unit strength	Daily dose	Posology	Route of admin.
**BI 207127**	tablet	Boehringer Ingelheim Pharma GmbH & Co. KG	200 mg	1200 mg; 800 mg	twice daily	oral
~~**BI 207127 matching placebo**~~	~~tablet~~	~~Temmler Werke GmbH, Munich, Germany~~	~~N/A~~	~~NA~~	~~twice daily~~	~~oral~~
**FDV**	soft gelatin capsule	Catalent Pharma solutions	120 mg	120 mg	once daily	oral
~~**FDV matching placebo**~~	~~soft gelatin capsule~~	~~Catalent Pharma solutions~~	~~N/A~~	~~NA~~	~~once daily~~	~~oral~~
**Ribavirin (Copegus®)**	tablet	F. Hoffmann-LaRoche Ltd	200 mg	1000 (<75 kg body weight); 1200mg (≥ 75 kg body weight)	twice daily	oral
~~**Ribavirin (Copegus®)**~~	~~tablet over-encapsulated~~	~~F. Hoffmann-LaRoche Ltd; encapsulation by Almac Clinical Services~~	~~200 mg~~	~~1000 (<75 kg body weight); 1200mg (≥ 75 kg body weight)~~	~~twice daily~~	~~oral~~
~~**Ribavirin matching placebo**~~	~~tablet over-encapsulated~~	~~Almac Clinical Services~~	~~N/A~~	~~NA~~	~~twice daily~~	~~oral~~

4.1.2 Method of assigning patients to treatment groups

After assessment of all entry criteria, the investigator or designee at the site will follow the process outlined in the ISF to assign the patient to a treatment group at visit 2. This will involve the use of an IRT system which will assign eligible patients to a treatment arm according to current screening stage of the trial (cf. Figure 3.1: 2). Treatment group assignment will be recorded on the eCRF and source document. For statistical features of the treatment allocation process refer to Section 7.5.

~~IRT will assign the first 48 patients randomised in Cohort B to participate the PK sub-study (cf. Section 5.5 and Appendix 10.1).~~

Proprietary confidential information.
© 2014 Boehringer Ingelheim International GmbH or one or more of its affiliated companies. All rights reserved.
This document may not - in full or in part - be passed on, reproduced, published or otherwise used without prior written permission.

Proprietary confidential information © 2015 Boehringer Ingelheim International GmbH or one or more of its affiliated companies

4.1.3 Selection of doses in the trial

The doses selected to be evaluated in this trial are BI 201727 400 mg b.i.d. or BI 201727 600 mg b.i.d. along with FDV 120 mg q.d. and weight-based RBV.

In SOUND-C2, the doses of BI 207127 600 mg b.i.d. in combination with FDV 120 mg q.d. and RBV demonstrated a favourable safety profile with a low rate of discontinuations due to AEs, and the absence of moderate or severe skin events or discontinuations due to skin events (rash or photosensitivity).

Safety and efficacy results from SOUND-C2 in patients with compensated liver cirrhosis were favourable (cf. Section 2.3). However, a 2-fold increase in exposure of both FDV and BI 207127 in cirrhotic patients compared to non-cirrhotic patients was observed. As such, it is expected that FDV and BI 207127 exposure in CPB patients will be at least 2-fold higher than that of non-cirrhotic patients. Therefore, a 400 mg b.i.d. dose of BI 207127 in combination with 120 mg FDV q.d. and RBV in CPB cirrhotic patients is expected to achieve similar or higher exposure than 600 mg b.i.d. dose group in non-cirrhotic patients. ~~If this is not the case and exposure is lower, Arm 3 will be conducted to test the 600 mg b.i.d. dose in CPB patients before the start of Cohort B. The dose for Cohort B will be selected and released by the internal data review committee.~~

PK / pharmacodynamic (PD) analyses performed in SOUND-C1 and 2 indicate that a PK exposure higher than the one achieved with BI 207127 600 mg b.i.d. does not provide an efficacy benefit in target patient population but rather negatively affects the safety profile. In particular, PK trough levels achieved with the 600 mg b.i.d. dose of BI 207127 were already beyond the exposure-response threshold for GT1b virus. BI 207127 doses higher than 600 mg b.i.d. did not increase viral response but impacted negatively on the safety profile (more severe skin and GI events).

No BI 207127 induction dose will be used (cf. Section 2.3).

Based on the current understanding that cirrhotic patients require longer treatment durations and the lack of substantial clinical data in cirrhotic patients treated for 16 weeks in phase 2, patients in this trial are planned to receive treatment for 24 weeks only.

4.1.4 Drug assignment and administration of doses for each patient

For Cohort A (Arm 2 ~~and Arm 3~~ only), once an investigator establishes that a CPB patient meets all entry criteria, the investigator will send electronically to the TCM a written request for assignment of the treatment visit date. The investigator will be required to receive written approval from the sponsor before confirming the appointment for the treatment visit with the patient (cf. ISF for request/approval form to be used).

The investigator will make a randomisation call to IRT to randomise/assign an eligible patient. Dispensing of study drugs will begin at Visit 2.

Proprietary confidential information.
© 2014 Boehringer Ingelheim International GmbH or one or more of its affiliated companies. All rights reserved.
This document may not - in full or in part - be passed on, reproduced, published or otherwise used without prior written permission.

Proprietary confidential information © 2015 Boehringer Ingelheim International GmbH or one or more of its affiliated companies

IRT will assign the medication number(s). The assigned medication number(s) must be entered in the eCRF, and the corresponding medication unit(s) must be given to the patient. Each medication unit will have a unique medication number and more than one medication unit may be assigned by IRT dependent on the visit schedule. The visits at which medication is dispensed to patients are specified in the Flow Chart. For details please refer to the ISF.

FDV ~~(or matching placebo)~~ loading dose on Day 1 will include an additional 120 mg capsule for a total of 240 mg for the first active dose. FDV ~~(or matching placebo)~~ should be taken q.d. in the morning together with the morning doses of BI 207127 and RBV ~~(or matching placebo)~~, and together with food. The evening doses of BI 207127 and RBV ~~(or matching placebo)~~ should be taken together with food 12 hours after the morning dose.

Patients in all groups are planned to receive study drugs (active ~~or placebo~~) for a total of 24 weeks. Early treatment discontinuation rules for lack of viral response or other reasons are defined for all groups (cf. Section 3.3.4.1).

If either of FDV or BI 207127 needs to be discontinued early all three trial medications need to be discontinued. Dose reductions of FDV or BI 207127 are not allowed in this trial. The dose of RBV may be reduced or RBV may be temporarily discontinued due to anaemia (cf. Section 4.2.1) while still continuing FDV and BI 207127 treatment. However, RBV should be reintroduced even at a reduced dose as soon as clinically possible.

Treatment interruptions must be avoided as much as possible. If the combination therapy is interrupted due to an unavoidable reason, it should restart no later than 3 days after the treatment was interrupted. Treatment interruptions for more than 3 days are not allowed and in these cases, treatment has to be stopped permanently and an EOT visit should be performed (cf. Section 6.2.3).

4.1.5 Blinding and procedures for unblinding

4.1.5.1 Blinding

Cohort A

Treatment Arm 1~~,~~ **and** Arm 2~~, and Arm 3~~ will be open-label.

~~Cohort B~~

~~Study medications will be administered double blinded in treatment Arm 4 and Arm 5.~~

~~It is recognised that this blinding will be challenging because of multiple laboratory test changes, specifically viral load, bilirubin, liver enzymes and hematologic changes that are related to the administration of active triple therapy *vs.* no such changes in patients with placebo. However, it is important to maintain the blinding through the blinding of specific laboratory assessments (cf. Table 4.1.5.1: 1) to minimize assessment bias as well as to ensure compliance with study procedures, retain the patients in the study, and control for AEs inherent in the target population.~~

Proprietary confidential information.
© 2014 Boehringer Ingelheim International GmbH or one or more of its affiliated companies. All rights reserved.
This document may not - in full or in part - be passed on, reproduced, published or otherwise used without prior written permission.

Proprietary confidential information © 2015 Boehringer Ingelheim International GmbH or one or more of its affiliated companies

~~In order to mitigate the risk of indirect unblinding and to maintain the integrity of the blinded assessments, Arm 4 and Arm 5 results of plasma HCV RNA levels and a series of safety laboratory tests after start of the treatment will be blinded to investigators, patients and sponsor for up to Week 24 visit (non-inclusive, i.e., Week 24 results will be reported to investigators) (cf. Table 4.1.5.1: 1). These safety laboratory results will be made available to the investigator if any of the criteria are met as described in Table 4.1.5.1: 1.~~

~~Central laboratory service will disclose the results of safety laboratory parameters when the unblinding criteria for those laboratory tests are met. If any of ALT or AST meets the criteria for unblinding, results for both laboratory tests will be disclosed from that time-point forward. If Hb meets the criteria for unblinding results for RBC count, Hb, hematocrit will be disclosed from that time point forward. If for the patient care management the investigator needs to know the blinded laboratory results even prior meeting the criteria established in Table 4.1.5.1: 1, the investigator will be able to request these laboratory tests (except HCV RNA) from central laboratory service after consultation with BI Clinical Monitor.~~

~~Central laboratory service will inform the investigator whether the patient meets lack of on treatment viral response criteria based on HCV RNA, without disclosing the actual results of HCV RNA. If any of these stopping rules are met, the investigator will receive a notification for repeat testing or treatment discontinuation.~~

~~Note that treatment code will be only broken upon investigator's request as per procedures described in Section 4.1.5.2.~~

~~Table 4.1.5.1: 1 Blinded laboratory tests for Arm 4 and Arm 5 from after start of treatment to up to Week 24 visit (non-inclusive) and criteria for unblinding~~

~~**Blinded laboratory parameters**~~	~~**Criteria for unblinding the laboratory parameters**~~
~~HCV RNA~~	~~All stopping rules (cf. Section 3.3.4.1)~~
~~ALT~~	~~> 2x of baseline with an absolute increase of at least 100 IU/L compared to baseline~~
~~AST~~	~~> 2x of baseline with an absolute increase of at least 100 IU/L compared to baseline~~
~~Bilirubin (total, direct and indirect)~~	~~Bilirubin > 3 mg/dL with a ratio of direct/indirect bilirubin >1~~
~~RBC laboratory assessment (RBC count, Hb, hematocrit)~~	~~Hb <10.5 g/dL; or, a Hb drop ≥ 2 g/dL during any 4 week period~~

~~For Cohort B, the sponsor, patient, and investigator will be unblinded to all laboratory values and treatment (active or placebo) for each patient at their EOT visit.~~

Proprietary confidential information.
© 2014 Boehringer Ingelheim International GmbH or one or more of its affiliated companies. All rights reserved.
This document may not - in full or in part - be passed on, reproduced, published or otherwise used without prior written permission.

Proprietary confidential information © 2015 Boehringer Ingelheim International GmbH or one or more of its affiliated companies

~~According to BI's SOPs, suspected unexpected serious adverse reactions (SUSARs) will be unblinded for reporting to competent authorities. In such cases, access to the code will only be permitted by authorized drug safety representatives via IRT.~~

4.1.5.2 Procedures for emergency unblinding

Not applicable.

~~An emergency code break will be available to the investigator/pharmacist/investigational drug storage manager via IRT. Details are included in the ISF. This code break may only be used in emergency situations when the identity of the trial drug must be known to the investigator in order to provide appropriate medical treatment or if required to assure safety of trial participants.~~

~~If the code break for a patient is used, the sponsor must be informed immediately. The reason for using the code break, the date when the code was broken and initials of person who broke the code must be documented on the appropriate electronic case report form (eCRF) page.~~

4.1.6 Packaging, labelling, and re-supply

BI 207127 ~~(or matching placebo)~~ are packaged in a carton box containing 2 bottles of 90 tablets each, for a total of 180 tables per medication unit.

FDV (also known as BI 201335~~, or matching placebo~~) are packaged in a bottle of 60 soft gelatine capsules per medication unit.

RBV (Copegus®) are packaged in a carton box containing 3 bottles of 60 tablets each, for a total of 180 tablets per medication unit.

~~RBV (Copegus®) over-encapsulated (or matching placebo) are packaged in a carton box containing 3 bottles of 60 tablets each, for a total of 180 tablets per medication unit.~~

Supply and re-supply will be managed by IRT.

For details of packaging and the description of the label, refer to the ISF.

4.1.7 Storage conditions

Drug supplies will be kept in their original packaging under the recommended storage conditions indicated on the label. Storage temperature should be monitored and documented.

The trial medication must be stored securely, e.g. in a locked cupboard or at a pharmacy. It may only be dispensed to trial patients according to the protocol by authorised personnel as documented in the form "Investigator's Trial Staff".

All unused medication must be returned to the sponsor. Receipt, usage and return must be documented on the respective forms. Account must be given for any discrepancies.

Proprietary confidential information.
© 2014 Boehringer Ingelheim International GmbH or one or more of its affiliated companies. All rights reserved.
This document may not - in full or in part - be passed on, reproduced, published or otherwise used without prior written permission.

Proprietary confidential information © 2015 Boehringer Ingelheim International GmbH or one or more of its affiliated companies

4.1.8 Drug accountability

Drug supplies, which will be provided by the sponsor or a CRO appointed by the sponsor, must be kept in a secure, limited access storage area under the storage conditions defined by the sponsor. Where necessary, a temperature log must be maintained to make certain that the drug supplies are stored at the correct temperature.

The investigator/pharmacist/investigational drug storage manager will receive the investigational drugs delivered by the sponsor when the following requirements are fulfilled:

- approval of the study protocol by the Institutional Review Board (IRB) / Independent Ethics Committee (IEC),
- availability of a signed and dated clinical trial contract between the sponsor and the Head of Trial Centre,
- approval/notification of the regulatory authority, e.g. competent authority (CA),
- availability of the Principal Investigator's (PI's) CV,
- availability of a signed and dated CTP or immediately imminent signing of the CTP (in exceptional cases, medication could already be sent to the site, before its activation via IRT),
- availability of the proof of a medical licence for the PI, if applicable
- availability of the Form 1572 (for USA).

The investigator/pharmacist/investigational drug storage manager must maintain records of the product's delivery to the trial site, the inventory at the site, the use by each patient, and the return to the sponsor or alternative disposition of unused product(s).

These records will include dates, quantities, batch/serial numbers, expiry ('use by') dates, and the unique code numbers assigned to the investigational product(s) and trial patients. The investigator/pharmacist/investigational drug storage manager will maintain records that document adequately that the patients were provided the doses specified by the CTP and reconcile all investigational product(s) received from the sponsor. At the time of return to the sponsor or appointed CRO, the investigator/pharmacist/investigational drug storage manager must verify that all unused or partially used drug supplies have been returned by the clinical trial patient and that no remaining supplies are in the investigator's possession.

4.2 CONCOMITANT THERAPY AND RESTRICTIONS, AND RESCUE TREATMENT

4.2.1 Rescue medication, emergency procedures, and additional treatments

If either of FDV or BI 207127 needs to be discontinued early, all three trial medications need to be discontinued (for further details cf. Section 4.1.4). If RBV-related severe adverse reactions or laboratory abnormalities develop during combination therapy, the dose of RBV may be reduced or RBV may be temporarily discontinued until the adverse reactions abate while still continuing FDV or BI 207127 treatment.

Proprietary confidential information.
© 2014 Boehringer Ingelheim International GmbH or one or more of its affiliated companies. All rights reserved.
This document may not - in full or in part - be passed on, reproduced, published or otherwise used without prior written permission.

Proprietary confidential information © 2015 Boehringer Ingelheim International GmbH or one or more of its affiliated companies

All dose adjustments of RBV, discontinuations and rescue medications must be documented in the patient source documentation and corresponding eCRF.

Management of drug-related skin events:

In case of drug-related skin events, the investigator should follow the dermatologic management plan, as specified in Appendix 10.2.

Management of gastrointestinal (GI) events:

GI AEs such as nausea, vomiting or diarrhoea have been reported during treatment with BI 207127 and FDV. The highest onset rate of these AEs was during the first week of treatment, particularly Day 1 and 2, followed by a significant decrease over time.

The following general measures are strongly recommended in order to prevent and manage these AEs and maintain adequate hydration:

- BI 207127 doses should be taken along with food avoiding intake in fasting conditions. Patients should be instructed to take their medications with food, preferably during breakfast and dinner.
- Early symptomatic treatment is recommended particularly during the first days of treatment and should be initiated for GI AEs as early as possible.
- Antiemetic treatments such as metoclopramide 10 mg tablets (orally dissolved) one tablet b.i.d. or q8h are recommended for nausea and vomiting. These agents may be taken approximately 30 minutes before BI 207127 intake if needed, and continued depending upon response. An antiemetic should be applied as soon as the first symptoms of nausea appear, even if mild. Re-taking study medications after vomiting is not recommended; patients should follow their regular schedule.
- Antimotility agents such as loperamide 2 mg caplets (or capsules) are recommended for non-infectious diarrhea; starting with 4 mg dose (2 caplets) and continue with 2 mg after each subsequent loose stool but no more than 4 caplets in 24 hours.
- Dietary and oral re-hydration measures are indicated based on investigator's clinical judgment, with special attention on patients 65 years old or older and with concomitant diuretic treatment

Management of anaemia

For RBV dose modification guidance for management of anaemia cf. to Table 4.2.1: 1. ~~Note that even though Hb values are blinded for Cohort B, investigator will be receiving laboratory reports if a patients experience a Hb <10.5 g/dL or, a Hb drop ≥ 2 g/dL during any 4 week period (cf. Table 4.1.5.1: 1).~~

Proprietary confidential information.
© 2014 Boehringer Ingelheim International GmbH or one or more of its affiliated companies. All rights reserved.
This document may not - in full or in part - be passed on, reproduced, published or otherwise used without prior written permission.

Proprietary confidential information © 2015 Boehringer Ingelheim International GmbH or one or more of its affiliated companies

Table 4.2.1: 1 Ribavirin hemoglobin dosage modification guidelines

Laboratory Values	Reduce only Ribavirin dose to 600mg/day[1], if:	Discontinue Ribavirin[1], if:
Hb in patients with no cardiac disease	<10 g/dL	<8.5 g/dL
Hb in patients with history of stable cardiac disease	≥2 g/dL decrease in Hb during any 4-week period treatment	<12 g/dL despite 4 weeks at reduced dose

[1] If the abnormality is reversed, Ribavirin should be restarted at 600 mg daily, and further increased to 800 mg daily at the discretion of the treating physician. However, a return to higher doses is not recommended.

Of note, other ribavirin dosage modification guidelines due to anaemia may apply according to local approved Copegus® label.

The use of eyrthropoiesis stimulating agents (ESA), is permitted at the discretion of the investigator. If ESA are being prescribed, the following guidelines have to be followed:

- ESA should not be initiated until haemoglobin levels falls below 10g/dL
- Iron studies should be obtained prior to and during ESA treatment
- Iron supplementation should be initiated for deficient patients and to maintain transferring saturation at levels supporting erythropoiesis
- Once an ESA is initiated, haemoglobin levels and blood pressure must be monitored weekly until stabilisation of haemoglobin
- Treatment should target a haemoglobin level sufficient to avoid transfusion
- ESA dose should be titrated to treatment response
- ESA dose should be reduced if the haemoglobin rises by more than 1 g/dL in a 2-week period
- ESA dose should not exceed those recommended for currently approved indications
- ESA should not be used in patients at increased risk for thromboembolic events, cardiovascular events, including those with inadequately controlled hypertension, and patients with diagnosed malignancies
- Patients must be informed about the risks associated with ESA use, as described in the patient information
- Dose and duration of treatment must be documented in the eCRF

Proprietary confidential information.
© 2014 Boehringer Ingelheim International GmbH or one or more of its affiliated companies. All rights reserved.
This document may not - in full or in part - be passed on, reproduced, published or otherwise used without prior written permission.

Proprietary confidential information © 2015 Boehringer Ingelheim International GmbH or one or more of its affiliated companies

~~Treatment option for patients that complete Arm 4~~

~~Eligible patients who accept the offer to receive active treatment (corresponding to Arm 5) will follow the visit order and procedures specified in Table 4.2.1: 2 as well as the treatment discontinuation criteria as defined in Section 3.3. Refer to ISF for further details.~~

~~Table 4.2.1: 2 Arm 4 treatment option schedule~~

	Treatment Period						A-FU[2]	
Visit	A1	A2	A3	A4	A5	A-EOT	A-FU1	A-FU2
Week	0	4	8	12	16	24	A-EOT +12	A-EOT +24
Day	1	29 ±2	57 ±2	85 ±2	113 ±3	169 ±3	A-EOT +84 -2/+6	A-EOT +168 -2/+6
Eligibility assessment	X							
Treatment assignment via IRT	X							
Pregnancy screening in females of childbearing potential	X	X	X	X	X	X	X	X
Physical examination[5]	X[T]	X[T]	X[T]	X[T]	X[T]	X[T]	X[T]	X[T]
HCV RNA	X	X	X	X	X	X	X	X
Virology samples	X	X	X	X	X	X	X	X
Laboratory tests[1]	X	X	X	X	X	X	X	X
Assess liver disease progression[6]	X	X	X	X	X	X	X	X
SAEs[4]	X	X	X	X	X	X	X	X
Dispense medications	X[3]	X	X	X	X			

~~1 Selected laboratory tests will be performed (cf. Laboratory Manual)~~
~~2 FU (follow-up) visits are counted from EOT (end of treatment).~~
~~3 Loading dose of FDV is needed.~~
~~4 All AEs with onset date up to 4 weeks after A-EOT will be recorded. Only related to FDV or related to BI 207127 or fatal SAEs will need to be reported through SAE form if onset date is 4 weeks after A-EOT.~~
~~5 "X[T]" is a targeted physical examination including measurements of vital signs, and evaluation of organ systems particularly associated with AE(s) symptoms.~~
~~6 For details on assessment of liver disease progression refer to Section 5.3.2.2.~~

4.2.2 Restrictions

4.2.2.1 Restrictions regarding concomitant treatment

Concomitant use of medications should be guided by the '*restricted*' and '*use with caution*' drug lists provided in the ISF.

Concomitant immunomodulatory treatment, including the chronic administration of systemic corticosteroids, is discouraged and if clinically possible should be postponed until EOT. Note that topical, nasal, or pulmonary steroids may be used (if not listed as restricted in ISF).

Systemic antiviral treatment will not be allowed during treatment period this trial, with the exception of oral antiviral such as acyclovir, famiclovir or valacyclovir for mild, localised recurrent herpes simplex infection, or oseltamivir or zanamivir for Influenza A.

Proprietary confidential information.
© 2014 Boehringer Ingelheim International GmbH or one or more of its affiliated companies. All rights reserved.
This document may not - in full or in part - be passed on, reproduced, published or otherwise used without prior written permission.

Proprietary confidential information © 2015 Boehringer Ingelheim International GmbH or one or more of its affiliated companies

The use of silibinin or other medications extracted from the milk thistle is not allowed during the treatment period of this trial.

The co-administration of drugs which may cause photosensitivity reactions should be avoided throughout the treatment duration and up until 28 days after all treatment discontinuation, unless the benefit outweighs the risk based on the investigators' clinical judgment. The restriction of drugs with photosensitivity potential includes and is not limited to tetracyclines (e.g. tetracycline, doxycycline), fluoroquinolones (e.g. ciprofloxacin, ofloxacine), sulfonamides, sulfonylureas (e.g. glipizide), psoralens, oral retinoids (e.g. isotretinoin, acitretin), phenothiazines and amiodarone.

All concomitant and/or rescue therapies will be recorded on the appropriate pages of the eCRFs.

4.2.2.2 Restrictions on diet and life style

Patients must be willing to protect any uncovered skin area (including hands, face and lips) from sun- or UV-light exposure using sun-blocker cream with an SPF ≥50 providing UV-A and -B protection during treatment period on a daily basis. Lip balm with the highest SPF locally available should be used. Patients should avoid unnecessary or prolonged exposure to sunlight and wear protective clothing, sunglasses and hats in addition to sun-block. It is recommended to avoid direct sun exposure as much as possible. Tanning booths must be avoided during treatment period.

No alcohol consumption during screening, treatment, and follow-up periods of the trial.

4.3 TREATMENT COMPLIANCE

Study medication will be dispensed to the patient at the study site by responsible site personnel. Details regarding dispensing of the study medication to each participating patient, including patient identification, the amount of study drug dispensed, the date the drug was dispensed, and the numbers of capsules/tablets/vials returned to the site will be recorded in the drug dispensing log. All dispensed study drug should be recorded in the drug-dispensing log in the ISF.

Patients must return to the site drug packaging including unused or empty medication packages at each visit. Study medication usage and return must be documented on the respective form and account must be given for any discrepancies.

Treatment compliance for all three drugs will be strictly monitored and assessed at each relevant visit through pill-count by Investigator (or designated site personnel) according to worksheets provided in ISF. For more information regarding FDV pill-count procedure, please cf. ISF. Compliance worksheets must be kept as a source documents. BI Clinical Monitor should be contacted for cases of suspected non-compliance.

Compliance will be calculated according to the formula:

Proprietary confidential information.
© 2014 Boehringer Ingelheim International GmbH or one or more of its affiliated companies. All rights reserved.
This document may not - in full or in part - be passed on, reproduced, published or otherwise used without prior written permission.

Proprietary confidential information © 2015 Boehringer Ingelheim International GmbH or one or more of its affiliated companies

- Compliance (%) = Number of medications actually taken since last count / Number of medications which should have been taken in the same period ×100%

Planned interruptions and dose reductions in treatment (applicable for RBV) in accordance with the protocol and prescribed by the investigator should be taken into account when calculating compliance.

If the treatment compliance cannot be assessed in a specific visit (e.g., bottle is lost or forgotten), the eCRF compliance page at that visit should be left blank.

Compliance to the BI 207127, FDV, RBV and matching placebos should be between 80% and 120%. Patients who are non-compliant according to this definition will be treated as protocol violations. The identification, handling and inclusion/exclusion of these and other protocol violations will be discussed in the trial statistical analysis plan (TSAP).

Patients who are non-compliant (e.g., have low treatment compliance, do not attend trial visits or violate the restrictions) may be withdrawn from the trial and the eCRF will be completed accordingly (for further procedures cf. Section 3.3.4), following discussion with BI Clinical Monitor.

Patients should take their medication regularly at the same time every day (BI 207127 and RBV every 12-hour and FDV every 24-hour). Excursions ± 1 hour outside from the regular times of intake should be avoided since this may lead to viral resistance and treatment failure.

If a patient misses a scheduled BI 207127 dose, and discovers this error:

- Less than 6 hours after the scheduled dose time, the patient must: take the missed dose; take all remaining doses at scheduled time; and, report the error at the next study visit.
- 6 hours or more after the scheduled dose time, the patient must: take the next scheduled dose at the next scheduled time; not take the missed dose; and, report the error at the next study visit.

If a patient misses a scheduled FDV dose, and discovers this error:

- Less than 12 hours after the scheduled dose time, the patient must: take the missed dose; take all remaining doses at scheduled time; and, report the error at the next study visit.
- 12 hours or more after the scheduled dose time, the patient must: take the next scheduled dose at the next scheduled time; not take the missed dose; and, report the error at the next study visit.

If a patient misses a scheduled RBV dose, and discovers this error:

- Less than 6 hours after the scheduled dose time, the patient must: take the missed dose; take all remaining doses at scheduled time; and, report the error at the next study visit.

Proprietary confidential information.
© 2014 Boehringer Ingelheim International GmbH or one or more of its affiliated companies. All rights reserved.
This document may not - in full or in part - be passed on, reproduced, published or otherwise used without prior written permission.

Proprietary confidential information © 2015 Boehringer Ingelheim International GmbH or one or more of its affiliated companies

- 6 hours or more after the scheduled dose time, the patient must: take the next scheduled dose at the next scheduled time; not take the missed dose; and, report the error at the next study visit.

Proprietary confidential information.
© 2014 Boehringer Ingelheim International GmbH or one or more of its affiliated companies. All rights reserved.
This document may not - in full or in part - be passed on, reproduced, published or otherwise used without prior written permission.

Proprietary confidential information © 2015 Boehringer Ingelheim International GmbH or one or more of its affiliated companies

5. VARIABLES AND THEIR ASSESSMENT

5.1 EFFICACY - CLINICAL PHARMACOLOGY

5.1.1 Endpoints of efficacy

Primary efficacy endpoint:

- SVR at Week 12 post-treatment (SVR12): Plasma HCV RNA level <25 IU/mL at 12 weeks after EOT

Secondary efficacy endpoints:

- SVR4: Plasma HCV RNA level <25 IU/mL at 4 weeks after EOT
- ~~SVR24: Plasma HCV RNA level <25 IU/mL at 24 weeks after EOT~~

Note: Patients with *HCV RNA level <25 IU/mL, detected* during the FU period should have a HCV RNA retest as soon as possible (within two weeks). Further retests are needed until *HCV RNA ≥25 IU/mL* (relapse) or *HCV RNA <25 IU/mL, undetected* is obtained.

Other efficacy endpoints:

- Virological Response (VR) at Week 4:

  -Plasma HCV RNA undetected at Week 4 ($W4U_{TND}$)

  -Plasma HCV RNA level <25 IU/mL at Week 4 (W4U)

- Change in Child-Turcotte-Pugh or MELD score by ≥1 point 12 weeks after EOT
- Plasma HCV undetected at Week 12 ($W12U_{TND)}$
- End of Treatment Response: Plasma HCV RNA level undetected at end of all therapy (ETR_{TND})
- Time to achieving HCV RNA undetected
- Time on treatment with HCV RNA undetected
- Virologic breakthrough (as defined in Section 3.3.4.1)
- Lack of on-treatment viral response (as defined in Section 3.3.4.1)
- Time to virologic breakthrough (from first HCV RNA undetected)
- Relapse: HCV RNA level > 25 IU/mL during the post-treatment period in patients who had ETR_{TND} at the end of the planned treatment
- Biochemical response

Proprietary confidential information.
© 2014 Boehringer Ingelheim International GmbH or one or more of its affiliated companies. All rights reserved.
This document may not - in full or in part - be passed on, reproduced, published or otherwise used without prior written permission.

Proprietary confidential information © 2015 Boehringer Ingelheim International GmbH or one or more of its affiliated companies

-ALT and aspartate aminotransferase (AST) in normal range at EOT

-ALT and AST in normal range post-treatment

- Events of liver disease progression (cf. Section 5.3.2.2)

5.1.2 Assessment of efficacy

A plasma sample will be obtained from all patients at each visit for the measurement of HCV RNA. The plasma HCV RNA level will be measured using the quantitative Roche COBAS® Taqman HCV/HPS assay (Version 2), a test that utilises real-time reverse transcription polymerase chain reaction (PCR) technology. The assay has a limit of detection between 10 and 20 IU/mL and a linear range of 25 IU/mL to 3.91×10^8 IU/mL.

The results below the linear range, HCV RNA levels <25 IU/mL, will be reported as being *undetected* or *detected*. For assessment of HCV RNA related endpoints, HCV RNA level <25 IU/mL is regardless whether HCV RNA is detected or undetected. HCV RNA undetected will be reported as *<25 IU/mL, undetected*.

Detailed instructions for obtaining, handling, and shipping HCV RNA measurement samples are provided in the ISF. Samples will be processed by a central laboratory service.

5.2 SAFETY

5.2.1 Endpoints of safety

The following safety endpoints will be assessed:

- AEs, including Division of Acquired Immunodeficiency Syndrome (DAIDS) grades
- AEs leading to treatment discontinuation of all trial medications
- SAEs
- Laboratory test abnormalities by DAIDS grades
- Change in laboratory test values over time

5.2.2 Assessment of adverse events

5.2.2.1 Definitions of adverse events

Adverse event

An AE is defined as any untoward medical occurrence, including an exacerbation of a pre-existing condition, in a patient in a clinical investigation who received a pharmaceutical product. The event does not necessarily have to have a causal relationship with this treatment.

Proprietary confidential information.
© 2014 Boehringer Ingelheim International GmbH or one or more of its affiliated companies. All rights reserved.
This document may not - in full or in part - be passed on, reproduced, published or otherwise used without prior written permission.

Proprietary confidential information © 2015 Boehringer Ingelheim International GmbH or one or more of its affiliated companies

Serious adverse event

An SAE is defined as any AE which results in death, is immediately life-threatening, results in persistent or significant disability / incapacity, requires or prolongs patient hospitalisation, is a congenital anomaly / birth defect, or is to be deemed serious for any other reason if it is an important medical event when based upon appropriate medical judgement which may jeopardise the patient and may require medical or surgical intervention to prevent one of the other outcomes listed in the above definitions.

Intensity of adverse event

All AEs with the exception of photosensitivity reaction will be graded according to DAIDS grading scale (see DAIDS grading scale in ISF (R10-1332)). Photosensitivity reactions will be graded according to a scale provided in the Dermatology Management Plan in Appendix 10.2.

The intensity of the AE should be judged based on the DAIDS Grading Scale:

- Mild: Grade 1
- Moderate: Grade 2
- Severe: Grade 3
- Potentially Life Threatening: Grade 4

Causal relationship of adverse event

Medical judgment should be used to determine the relationship, considering all relevant factors, including pattern of reaction, temporal relationship, de-challenge or re-challenge, confounding factors such as concomitant medication, concomitant diseases and relevant history. Assessment of causal relationship should be recorded in eCRFs.

Yes: There is a reasonable causal relationship between the investigational product administered and the AE.
No: There is no reasonable causal relationship between the investigational product administered and the AE.

If a SAE is reported from a still blinded trial, the causal relationship must be provided by the investigator for all potential trial drugs, i.e. the BI trial drug and for all other trial drugs (i.e. any active comparator or placebo according to the trial design).

Worsening of the underlying disease or other pre-existing conditions

Worsening of the underlying disease or of other pre-existing conditions will be recorded as an (S)AE in the eCRF.

Changes in vital signs, ECG, physical examination, and laboratory test results

Changes in vital signs, ECG, physical examination and laboratory test results will be recorded as an (S)AE in the (e)CRF , if they are judged clinically relevant by the investigator.

Proprietary confidential information.
© 2014 Boehringer Ingelheim International GmbH or one or more of its affiliated companies. All rights reserved.
This document may not - in full or in part - be passed on, reproduced, published or otherwise used without prior written permission.

Proprietary confidential information © 2015 Boehringer Ingelheim International GmbH or one or more of its affiliated companies

~~As described in Section 4.1.5.1 selected laboratory tests results in Arm 4 and 5 will be blinded to investigator, patients and sponsor. Therefore, it is understood that by design investigator will not be able to report laboratory related AEs, unless they are in the range that require unbinding of the investigator to the results (cf. Table 4.1.5.1: 1). The blinding ranges were set to exclude values that may be clinically relevant to avoid an effect on the AE reporting rates. In the Clinical Trial Report (CTR), all laboratory tests will be analyzed and presented to demonstrate any potential effect of laboratory test blinding on AE reporting rates.~~

Protocol-specified significant events

The following are considered as protocol-specified significant events:

- Skin rash cases of Grade ≥2 severity or photosensitivity reactions of moderate or greater severity.

Protocol-specified significant events are to be reported in an expedited manner similar to SAEs, even if they do not meet any of the seriousness criteria. For details please see Section 5.2.2.2.

5.2.2.2 Adverse event and serious adverse event reporting

All AEs, serious and non-serious, with onset date from signing the informed consent until 28 days after all treatment discontinuation (FU1 visit), and all SAEs with onset date after FU1 visit up to EOO will be collected, documented and reported to the sponsor by the investigator on the appropriate eCRFs/SAE reporting forms. The period from EOT to 28 days after after EOT is called “residual effect period”. Reporting will be done according to the specific definitions and instructions detailed in the 'Adverse Event Reporting' section of the ISF.

For each AE, the investigator will provide the onset date, end date, intensity, treatment required, outcome, seriousness, and action taken with the investigational drug. The investigator will determine the relationship of the investigational drug to all AEs as defined in Section 5.2.2.1. For details refer to the subsections of this Section.

If not stipulated differently in the ISF, the investigator must report the following events 1) if using paper process SAE form via telephone/fax or 2) if available for the trial, using the electronic submission process (RDC) immediately (within 24 hours to the sponsor: SAEs and non-serious AEs which are relevant for a reported SAE(s), and protocol-specified significant events.

~~BI has set up a list of AEs which are defined to be always serious. In order to support the investigator with the identification of these “always serious adverse events”, if a non serious AE is identified to be serious per BI definition, a query will be raised. The investigator must verify the description and seriousness of the event. If the event description is correct, the item “serious” needs to be ticked and an SAE has to be reported in expedited fashion~~

Proprietary confidential information.
© 2014 Boehringer Ingelheim International GmbH or one or more of its affiliated companies. All rights reserved.
This document may not - in full or in part - be passed on, reproduced, published or otherwise used without prior written permission.

Proprietary confidential information © 2015 Boehringer Ingelheim International GmbH or one or more of its affiliated companies

~~following the same procedure as above. The list of these AEs can be found via the RDC-system.~~

The SAE form is to be forwarded to the defined unique entry point identified for the BI OPU (country-specific contact details will be provided in the ISF) or by using the electronic submission process. This immediate report is required irrespective of whether the investigational product has been administered or not and irrespective of causal relationship. It also applies if new information to existing SAEs or protocol-specified significant events becomes available.

Reporting AEs occurring up to 28 days after all treatment discontinuation (FU1 visit):

The investigator has the responsibility to report all AEs occurring up to 28 days after all treatment discontinuation (FU1 visit), regardless whether they are serious, non-serious, related or not-related to the trial medication. Any AEs persisting after FU1 visit must be followed until they have resolved, or as deemed reasonable by the investigator in consultation with BI Clinical Monitor. These AEs will be considered "on-treatment"~~, except if the onset date was after the start of the active treatment option (for Arm 4 patients only~~).

Reporting AEs occurring later than 28 days after all treatment discontinuation (FU1 visit):

AEs that occur after FU1 visit will be reported only if serious. These events will be attributed to the "post-treatment period."

The investigator does not need to actively monitor patients for adverse events once the clinical trial has ended (after EOO). However, if the investigator becomes aware of an SAE(s) that occurred after the patient has completed the clinical trial, it should be reported by the investigator to the sponsor if considered relevant by the investigator. These events will be attributed to the "post-study period."

Pregnancy

In rare cases, pregnancy might occur in clinical trials. Once a female subject has been enrolled into the clinical trial, after having taken study medication, the investigator must report immediately any drug exposure during pregnancy to the sponsor. Drug exposure during pregnancy has to be reported immediately (within 24 hours) to the defined unique entry point for SAE forms of the respective BI OPU (country-specific contact details will be provided in the ISF). The outcome of the pregnancy associated with the drug exposure during pregnancy must be followed up. In the absence of an (S)AE, only the Pregnancy Monitoring Form for Clinical Trials and not the SAE form is to be completed. The ISF will contain the Pregnancy Monitoring Form for Clinical Trials (Part A and Part B).

Reporting protocol-defined significant AEs

Skin AEs of moderate or greater severity deemed related to the trial medications should be reported as protocol-defined significant events. If not stipulated differently in the ISF, protocol-defined significant AEs (cf. Section 5.2.2.1) should be reported on the SAE/Significant AE form immediately (within 24 hours even if not serious) as well as on the

Proprietary confidential information.
© 2014 Boehringer Ingelheim International GmbH or one or more of its affiliated companies. All rights reserved.
This document may not - in full or in part - be passed on, reproduced, published or otherwise used without prior written permission.

Proprietary confidential information © 2015 Boehringer Ingelheim International GmbH or one or more of its affiliated companies

AE eCRF page and the skin eCRF page. Please note, the event should NOT be reported as serious on the SAE form unless it meets the regulatory definitions of a SAE defined in the "Adverse Event Reporting" section of the ISF. It is recommended that all patients with protocol-defined significant AEs should see be referred to a local dermatologist for diagnosis, confirmation of severity, and treatment advice. All patients with protocol-defined significant AEs should have a photo-documentation of their lesions. For instructions on photo-documentation, please refer to the ISF. For specific management and grading guidelines for patients developing rash and photosensitivity reactions, please cf. Appendix 10.2 and Dermatologic Operations Manual in the ISF.

Grade 1/mild skin AEs are not considered *"protocol-defined significant AEs"* and should be reported in the AE eCRF and skin eCRF. Skin AEs with a known etiology (e.g. viral exanthema, collagen vascular disease, neoplasia, bacterial infection, psoriasis, fungal skin infection, or autoimmune blistering disease) deemed not related to the trial medications are not considered *"protocol-defined significant AEs"* and should be reported in the AE eCRF page only. A skin eCRF page is not required.

Protocol-defined significant AEs will be subject to review by the dermatological adjudication committee.

BI has set up a list of AEs which are defined to be always serious. In order to support the investigator with the identification of these "always serious adverse events", if a non serious AE is identified to be serious per BI definition, a query will be raised. The investigator must verify the description and seriousness of the event. If the event description is correct, the item "serious" needs to be ticked and an SAE has to be reported in expedited fashion following the same procedure as above. The list of these AEs can be found via the RDC-system.

5.2.3 Assessment of safety laboratory parameters

The safety laboratory will be performed at the central laboratory service provider. Reference ranges and instructions on sample collection, handling/processing, and shipping will be provided in the ISF.

Patients should be fasting for at least 6 hours prior to the blood sample being taken (except screening visit). For time points of sampling refer to the Table 5.2.3: 1 and Flow Chart. For blinding of selected laboratory results cf. Section 4.1.5.1.

Clinically relevant laboratory values should be commented on lab report print-outs. A clinically relevant value may be either in- or outside the reference range. Clinically relevant abnormal laboratory test results must be confirmed using an unscheduled visit lab kit and should be repeated until normalisation or stabilisation or until an alternative explanation has been found.

The following laboratory results will be flagged by the central laboratory for further assessment of potential DILI cases, if observed at same time point:

- ALT or AST 2x baseline that is at least 100 IU/L higher than baseline, AND

Proprietary confidential information.
© 2014 Boehringer Ingelheim International GmbH or one or more of its affiliated companies. All rights reserved.
This document may not - in full or in part - be passed on, reproduced, published or otherwise used without prior written permission.

Proprietary confidential information © 2015 Boehringer Ingelheim International GmbH or one or more of its affiliated companies

- Total bilirubin >3x ULN combined with direct /indirect bilirubin >1

Table 5.2.3: 1 Laboratory tests

**Category**	**Test name**	
**Haematology**	Hematocrit (Hct)<br>Hb<br>RBC Count/Erythrocytes<br>Reticulocyte Count<br>WBC Count/Leukocytes<br>Platelet Count/Thrombocytes	
	Diff. Automatic (manual if diff. automatic is abnormal)	- Neutrophils<br>- Eosinophils<br>- Basophils<br>- Monocytes<br>- Lymphocytes
**Coagulation**	Partial Thromboplastin Time (aPTT)<br>Prothrombin time (Quick and INR)	
**Chemistry**	AST (GOT)<br>ALT (GPT)<br>Alkaline Phosphatase (AP)<br>Albumin<br>Creatine Kinase (CK)<br>CK-MB, only if CK is elevated<br>Gamma-Glutamyl Transferase (GGT/γ-GT)<br>Lactic Dehydrogenase (LDH)<br>Lipase<br>Amylase<br>Calcium<br>Sodium<br>Potassium<br>Chloride<br>Bicarbonate<br>Glucose<br>Creatinine<br>Creatinine clearance (calculated)<br>Bilirubin Total[4]<br>Bilirubin Direct[4]<br>Bilirubin Indirect[4]<br>Protein, Total<br>C-Reactive Protein<br>Cholesterol, total<br>Triglycerides<br>Haptoglobin, , only if Hb <10.5g/dL<br>Bile acids<br>Urea	

1 At Visit 1 only
2 At liver disease progression assessment time-points
3 Results will not be captured in the sponsor's database
4 DAIDS grading does not apply
5 IgE only in case of mild, moderate or severe skin events

Proprietary confidential information.
© 2014 Boehringer Ingelheim International GmbH or one or more of its affiliated companies. All rights reserved.
This document may not - in full or in part - be passed on, reproduced, published or otherwise used without prior written permission.

Proprietary confidential information © 2015 Boehringer Ingelheim International GmbH or one or more of its affiliated companies

Table 5.2.3: 1 (continued) Laboratory tests

Category	Test name
**Pregnancy test**	Human serum chorionic gonadotropin (up to 24-week FU)<br>Human urine chorionic gonadotropin (at Visit 2 only before the first study drugs intake)
**Urine-Sediment (microscopic examination), (only if urine analysis abnormal)**	Urine Sediment Bacteria<br>Urine Cast in Sediment<br>Urine Squamous Epithelial Cells<br>Urine Sed. Crys., Unspecified Urine Sediment RBC/Erythrocytes<br>Urine Sediment WBC/Leucocytes
**Tumour markers**[2]	Alpha-fetoprotein
**Autoimmune serum markers**[1]	Anti-nuclear antibodies (ANA)
**Other laboratory parameters**[1]	IP-10 (IFN-gamma inducible protein-10)<br>Glycosylated Hb (HbA1c)<br>Homeostatic model assessment of insulin resistance (HOMA-IR)<br>Parathyroid hormone<br>Vitamin D measurement<br>LDL, VLDV and HDL
**Drug screening (urine)**[1,3]	Cannabis<br>Benzodiazepine<br>Barbiturates<br>Opiates<br>Cocaine<br>Amphetamines<br>Methadone
**HCV, HBV, HIV testing**	Hepatitis B Surface Antigen (qualitative) [1,3]<br>Hepatitis C Antibodies (qualitative) [1,3]<br>HIV-1, and HIV-2 Antibody (qualitative) [1,3]<br>HCV RNA PCR (quantitative)
**Specific immunoglobulin (Ig) measurement**	Total serum IgE[5]

1 At Visit 1 only
2 At liver disease progression assessment time-points
3 Results will not be captured in the sponsor's database
4 DAIDS grading does not apply
5 IgE only in case of mild, moderate or severe skin events

5.2.3.1 Pregnancy tests

Serum pregnancy tests will be performed at screening (Visit 1) and at every visit up to 24-week follow-up for all female patients of childbearing potential. In addition to serum pregnancy test, a urine (dipstick) pregnancy test will be performed for all females before receiving the study drugs at Day 1 (Visit 2). Only female patients with negative urine pregnancy test will receive study medication at Day 1 (Visit 2). Urine (dipstick) pregnancy tests will not be captured in the database.

When visits are more than 4 weeks apart and/or due to early treatment termination, female patients of childbearing potential will be provided with urine pregnancy tests to perform monthly pregnancy tests at home between visits until 7 months after EOT. It is strongly recommended that female partners of childbearing potential of male patients also perform

Proprietary confidential information.
© 2014 Boehringer Ingelheim International GmbH or one or more of its affiliated companies. All rights reserved.
This document may not - in full or in part - be passed on, reproduced, published or otherwise used without prior written permission.

Proprietary confidential information © 2015 Boehringer Ingelheim International GmbH or one or more of its affiliated companies

monthly pregnancy test at home and the sponsor will provide urine (dipstick) pregnancy tests for this purpose.

5.2.4 Electrocardiogram

The 12-lead ECGs will be performed as scheduled in the Flow Chart.

It will be recorded after the patients have rested for at least 5 minutes in supine position at Visit 1. Six limb leads, as specified by Einthoven (I, II and III) and Goldberger (aVR, aVL, aVF), and six pre-cordial leads (V1–V6), according to Wilson, will be used. The investigator or a designate will evaluate whether the ECG is normal or abnormal and, if abnormal, whether it is clinically significant. Additionally, any occurrence of re- or depolarization disorders, arrhythmic disorders or other abnormalities will be assessed.

The electronic version of the ECG is regarded as source data. Dated and signed printouts will be stored in the patient's medical file.

ECGs may be repeated for quality reasons and the repeat used for analysis. Additional ECGs may be collected by the investigator for safety reasons, included but not limited to follow up of patients who experience palpitations, fainting, sudden loss of consciousness or syncope. Clinically relevant abnormal findings will be reported as AEs. The ECGs may be sent to the ECG laboratory to undergo central assessment.

5.2.5 Physical examination

Complete and target physical examinations will be carried out as described in the Flow Chart.

A complete physical exam consists of an evaluation of organ systems with vital signs (including respiratory rate, temperature, pulse rate, and systolic/diastolic blood pressure). Respiratory rate, temperature, pulse rate, and blood pressure will be measured after patients have been sitting comfortably for at least five minutes.

A targeted physical examination includes an evaluation of organ systems only associated with an AE symptom(s) (as needed,and particularly focused on hepatic-related events) and evaluation of vital signs.

Clinically relevant abnormal findings will be reported as AEs.

5.2.6 FibroSURE™

Non-invasive method for lived fibrosis assessment, FibroSURE™, will be assessed at the time-points specified in the Flow Chart. FibroSURE™ is an algorithm based on a composite of five serum biochemical markers (alpha-2-macroglobulin, apolipoprotein A1, haptoglobin, GGT and bilirubin) that is used to assess liver fibrosis with its different degrees. The algorithm generates a score that correlates with the level of fibrosis and the presence of cirrhosis.

Proprietary confidential information.
© 2014 Boehringer Ingelheim International GmbH or one or more of its affiliated companies. All rights reserved.
This document may not - in full or in part - be passed on, reproduced, published or otherwise used without prior written permission.

Proprietary confidential information © 2015 Boehringer Ingelheim International GmbH or one or more of its affiliated companies

5.3 OTHER

5.3.1 Other endpoints

5.3.1.1 Health Care Resource Utilization (HCRU) and Work Productivity

For the purpose of a separate health economic analysis (such as cost-utility analysis), HCRU data will be collected throughout the study. Resource use data collected for calculating direct costs will include unscheduled hospitalisations, healthcare provider visits, and emergency room/ICU use. Information on work productivity will also be collected.

The economic evaluation of the HCRU data will not be part of the CTR but reported separately.

5.3.1.2 EQ-5D

Health related quality of life will be assessed using the EQ-5D at the visits indicated on the Flow Chart. EQ-5D is a standardized instrument for use as a measure of health outcome. It is a generic measure, rather than disease-specific, and is therefore applicable to a wide range of health conditions and treatments. It provides a simple descriptive profile and a single index value for health status. EQ-5D is designed for self completion by patients.

The EQ-5D self-report questionnaire (EQ-5D) essentially consists of 2 pages comprising:

- The descriptive system (five dimensions of health; namely mobility, self care, usual activities, pain/discomfort, anxiety/depression). Each dimension comprises three levels (no problems, some/moderate problems, extreme problems).

- The EQ-VAS (visual analogue scale) which records the patient's self-rated health status on a vertical graduated (0 – 100) VAS.

The paper-and-pen version in the required native language of the patient will be used. If the required language is not available then the patient is not required to complete the questionnaire.

A patient can self-administer the EQ-5D in a few minutes, and the Investigator (or designated site-personnel) should ensure that the patient has access to a quiet area at the site where he can be left alone to record a response to the descriptive system and VAS. In instances where a patient cannot give or decide upon a response, no response should be recorded. The Investigator (or designated site-personnel) should check that all items have been completed by the patient, but the response to each item should not be scrutinized. Instructions to patients are included in the questionnaire.

Proprietary confidential information.
© 2014 Boehringer Ingelheim International GmbH or one or more of its affiliated companies. All rights reserved.
This document may not - in full or in part - be passed on, reproduced, published or otherwise used without prior written permission.

Proprietary confidential information © 2015 Boehringer Ingelheim International GmbH or one or more of its affiliated companies

5.3.2 Other assessments

5.3.2.1 Genotypic and Phenotypic Resistance

5.3.2.1.1 Genotypic Resistance

In this study of FDV and BI 207127 combination therapy, genotypic resistance monitoring (sequencing) will focus on the NS3/4A and NS5B genomic segments. Samples for genotyping the HCV NS3/4A protease and NS5B polymerase will be collected at screening (Visit 1) and at all patient visits. Viral genotyping will be performed for patients who discontinue study treatment. HCV RNA plasma viral load data will guide which additional samples are examined; genotyping will be performed on samples in which the HCV RNA plateaus above the lower limit of quantification or in which the HCV RNA rebounds during trial period (before or at EOO visit) and compared to baseline sequence. These include FU isolates (as detailed in Flow Chart) from individuals whose on- and post-treatment viral samples are genotyped to assess the persistence of resistance mutations during the outgrowth of wild-type virus.

The viral RNA will be extracted, cDNA synthesized and amplified using reverse transcriptase-PCR. The length of amplified product potentially limits the detection to samples with HCV RNA $>10^3$ IU/mL. The NS3/4A protease and NS5B polymerase nucleotide sequences will be obtained by direct DNA sequencing of the amplified product that allows for the detection of variants present at approximately ≥30%.

5.3.2.1.2 Phenotypic Resistance

The virology samples obtained for genotypic resistance monitoring are also suitable for phenotyping. NS3 protease or NS5B polymerase replicon-based phenotyping will be performed on samples in which the HCV RNA plateaus above the limit of quantification or in which the HCV RNA rebounds during trial period (before or at EOO visit) and which are associated with unique resistant mutants that have not been previously phenotyped. Representatives of the most common and well characterized pathways in assays will be included as references. In addition to patient-derived NS3 or NS5B samples, novel amino acid substitutions will also be characterized phenotypically using site-directed mutagenesis if they are observed repeatedly in genotyped subjects.

5.3.2.1.3 Methods and timing of sample collection

At least 4 mL of plasma from 8 mL of blood sample will be collected for virology samples (genotyping and phenotyping analyses) at each visit. These will be divided into two 2 mL aliquots: one primary and one back-up. Samples will be shipped to the central laboratory on dry ice. The central laboratory will store samples at -80°C until processing. Detailed instructions for obtaining, handling, and shipping virology samples are provided in ISF.

5.3.2.2 Assessment of liver disease progression

Markers of liver disease progression will be collected as occurrence of any of the following clinical outcomes and assessed at the time points specified in the Flow Chart:

Proprietary confidential information.
© 2014 Boehringer Ingelheim International GmbH or one or more of its affiliated companies. All rights reserved.
This document may not - in full or in part - be passed on, reproduced, published or otherwise used without prior written permission.

Proprietary confidential information © 2015 Boehringer Ingelheim International GmbH or one or more of its affiliated companies

- Child-Turcotte-Pugh score in points (cf. Appendix 10.3)
- MELD score in points
- New onset of ascites or change in severity from baseline
- New onset of hepatic encephalopathy or change in severity from baseline
- Liver cancer
- Esophageal variceal haemorrhage
- Spontaneous bacterial peritonitis
- Liver transplant
- Liver-related death

5.3.3 Pharmacogenomic evaluation

The first sample, a mandatory part of the protocol, collected at Visit 2 will be used for DNA extraction and subsequent genotyping of genotyping of IL-28b rs12979860, as well as other genes involved in the activity and/or efficacy of the drug within the context of the clinical trial. In addition, genes influencing assimilation and/or metabolism like UGT1A1, HLA class I and II molecules, CYP450 enzyme and drug transporter genes, like OATPs, genes influencing the therapeutic outcome like interferon signalling pathway genes and/or interferon stimulated genes may be evaluated. After successful analysis, the remaining material will be destroyed.

The second sample collected at Visit 2 sample is voluntary and will be taken and processed or stored only after informed consent is given in accordance with local ethical and regulatory requirements. This sample will be completely anonymised. The anonymization procedure will guarantee a very high level of data protection for the donor. Once the anonymization has been carried out, there will be no way to trace back to the identity of the donor through the coding keys. The anonymised DNA may be analysed at a later time to identify whether there are genetic factors that could contribute to a better therapeutic outcome or a higher risk of developing treatment-related adverse drug reactions. These analyses may include genes related to efficacy, safety and PK. After anonymization, the third sample (or the DNA derived thereof) will be stored at Boehringer Ingelheim for 15 years after the end of the clinical trial or until there is no more material available for tests.

5.3.3.1 Methods and timing of sample collection

One mandatory and one optional 8.5mL blood sample will be taken at Visit 2 for pharmacogenetic testing with PaxGene DNA blood sampling tubes. The Paxgene Blood DNA tubes can be stored and shipped at room temperature within 14 days. If a longer storage and shipment period for Paxgene Blood DNA tubes is necessary, the blood samples have to be stored at a temperature of -20° C or below. Once frozen, thawing of the samples should be avoided. Detailed instructions for pharmacogenetic sampling, handling and shipment of samples are provided in the ISF.

Proprietary confidential information.
© 2014 Boehringer Ingelheim International GmbH or one or more of its affiliated companies. All rights reserved.
This document may not - in full or in part - be passed on, reproduced, published or otherwise used without prior written permission.

Proprietary confidential information © 2015 Boehringer Ingelheim International GmbH or one or more of its affiliated companies

5.3.3.2 Analytical determinations

Genomic DNA will be extracted from blood samples according to standard molecular genetics methods and analyzed by TaqMan® or other standard genotyping technologies.

5.4 APPROPRIATENESS OF MEASUREMENTS

All measurements performed during this trial are standard measurements in HCV treatment trials and will be performed in order to monitor safety aspects or assess treatment response in an appropriate way.

The scheduled measurements are appropriate to see drug induced changes in vital signs, standard laboratory values and ECG. These primary and secondary endpoints are standard and accepted for evaluation of safety and tolerability of an oral drug, and they are widely used in this kind of studies.

Therefore, the appropriateness of all measurements applied in this trial is given.

5.5 DRUG CONCENTRATION MEASUREMENTS AND PHARMACOKINETICS

5.5.1 Pharmacokinetic endpoints

The following primary PK parameters will be calculated for patients participated in Cohort A:

- C_{max} (maximum measured concentration of BI 207127 & metabolites, FDV and RBV)
- AUC_{0-12} (area under the concentration-time curve of BI 207127 & metabolites and RBV in plasma over the time interval from 0 to 12 hour)
- AUC_{0-24} (area under the concentration-time curve of FDV in plasma over the time interval from 0 to 24 hour)
- C_{12} (plasma concentration of BI 207127 & metabolites and RBV at 12 hour)
- C_{24} (plasma concentration of FDV at 24 hour)

~~Other PK parameters will be determined for the analyte if feasible in Cohort A:~~

- t_{max} (time to reach maximum concentration C_{max} in plasma)
- ~~λ_z (terminal rate constant in plasma)~~
- ~~$t_{1/2}$ (terminal half-life of the analyte in plasma)~~
- ~~MRT_{po} (mean residence time of the analyte in the body)~~

Proprietary confidential information.
© 2014 Boehringer Ingelheim International GmbH or one or more of its affiliated companies. All rights reserved.
This document may not - in full or in part - be passed on, reproduced, published or otherwise used without prior written permission.

Proprietary confidential information © 2015 Boehringer Ingelheim International GmbH or one or more of its affiliated companies

- ~~CL/F (apparent clearance of the analyte in the plasma after extravascular administration)~~
- ~~V_z/F (apparent volume of distribution during the terminal phase λ_z)~~

~~In addition plasma protein binding of BI 207127 & metabolites and FDV will be determined in Cohort A only.~~

5.5.2 Methods of sample collection

The date and clock time of the most recent drug intakes, and date and clock time of the blood sample collection will be entered in the eCRF. Further details on collection, preparation, and shipping will be included in ISF.

5.5.2.1 Pharmacokinetic samples

For Cohort A, intensive PK blood samples will be collected at Day 1, 8 and Week 4 ~~and 16~~ for all patients. At visits with intensive PK sample collection, PK blood samples are collected at -0.05 (pre-dose), 0.5, 1, 1.5, 2, 3, 4, 5, 6, 8, 10, 12 and 24 hours after dosing (cf. Appendix 10.1.2, Table 10.1.2: 1).

~~For Cohort B, an intensive PK sub-study will be conducted to assess pre-dose and 4 post-dose samples at Day 1, 8 and Week 4 and 16 (cf. Appendix 10.1.2, Table 10.1.2: 2). A total of 48 patients will participate and 16 patients will be assigned to each of the following collection schedules via IRT: Day 1 and Day 8; Day 1 and Week 4; or Day 1 and Week 16 (cf. Section 4.1.2). The 4 post-dose samples will be taken at specific time period post-morning dose according to a pseudo-randomised scheme described in Appendix 10.1.2, Table 10.1.2: 2. The data from intensive PK sub-study for Cohort B will be tranferred to Pharmacometrics group in Translationational Medicine department for population pharmacokinetic analysis.~~

For all patients ~~in Cohort A and Cohort B~~, pre-dose PK blood samples will be collected according to the Flow Chart. If patient is subjected to intensive PK on same visit day, only collect intensive PK samples. Patients should not take study medication the mornings of treatment period visits. Administration of the study drug will be done under the supervision of the investigator or his designee after the pre-dose sample has been collected. The time elapsed since the last dose of FDV should be as close to 24 hours as possible and the last dose of BI 207127 should be as close to 12 hours as possible.

For quantification of FDV and RBV plasma concentration, 2.0 mL of blood will be drawn from a forearm vein in an EDTA-anticoagulant blood drawing tube. For quantification of BI 207127, CD 6168, BI 208333 (BI 207127 acylglucuride) and CD 6168-acylglucuronide, 4.5 mL of blood will be drawn from a forearm vein in a blood drawing tube containing citrate or citrate and citric acid.

~~5.5.2.2 Protein binding~~

Proprietary confidential information.
© 2014 Boehringer Ingelheim International GmbH or one or more of its affiliated companies. All rights reserved.
This document may not - in full or in part - be passed on, reproduced, published or otherwise used without prior written permission.

Proprietary confidential information © 2015 Boehringer Ingelheim International GmbH or one or more of its affiliated companies

~~For determination of protein binding of BI 207127 & metabolites and FDV (Arm 1, 2 and 3 in Cohort A only), two 10 mL blood samples will be drawn from a forearm vein in EDTA-anticoagulant blood drawing tubes at 2.5 h post dose at Day 1,8 and Week 4 and 16. Due to the resource capacity, only samples collected at Day 8 will be first used for protein binding assay. Remaining samples will be analyzed only if needed.~~

5.5.3 Analytical determinations

5.5.3.1 Pharmacokinetics

All analytes will be determined by a validated HPLC-MS/MS assay (high performance liquid chromatography, tandem mass spectrometry). Bioanalytical determinations for all the analytes will be performed by Tandem Labs (Salt Lake City, UT 84124).

FDV ZW: The concentration of FDV ZW in EDTA plasma will be determined by a validated HPLC/MS/MS (high performance liquid chromatography, tanden mass spectrometry) method.

BI 207127: The concentrations of BI 207127 and its metabolites in citrated/citric acid plasma will be determined by a validated HPLC/MS/MS (high performance liquid chromatography, tandem mass spectrometry) method.

RBV: The concentrations of RBV in EDTA plasma will be determined by a validated HPLC/MS/MS (high performance liquid chromatography, tandem mass spectrometry) method.

Proprietary confidential information.
© 2014 Boehringer Ingelheim International GmbH or one or more of its affiliated companies. All rights reserved.
This document may not - in full or in part - be passed on, reproduced, published or otherwise used without prior written permission.

Proprietary confidential information © 2015 Boehringer Ingelheim International GmbH or one or more of its affiliated companies

6. INVESTIGATIONAL PLAN

6.1 VISIT SCHEDULE

All patients are to adhere to the visit schedule as specified in the Flow Chart. Each visit date (with its window) is to be counted from Day 1. Additional visits for the purpose of re-testing of laboratory parameters or AE monitoring may be included as deemed necessary by the investigator.

6.2 DETAILS OF TRIAL PROCEDURES AT SELECTED VISITS

For detailed description of the trial procedures to be performed at each visit, refer to the Flow Chart and respective protocol sections. Explanations of procedures are provided in Section 5. Additional details regarding visit procedures are provided below.

All visits, except Visit 1 (screening), must be performed in fasted state.

6.2.1 Screening and run-in periods

No procedures should be done unless the patient has provided written informed consent in accordance with GCP and local legislation to taking part in the trial.

Once consented, a patient is considered to be enrolled in the trial and have started screening. The patient should be assigned a patient number, recorded on the enrolment log, registered in IRT, and recorded on the eCRF as a screened patient.

Screening (Visit 1) should normally take place no more than 42 days before Visit 2; however the time window for Visit 1 may be extended at the discretion of the CML in conjunction with the TCM on a case by case basis.

Re-screening a patient will not be permitted. Patients who have a laboratory test value outside the range specified by the entry criteria may have the test repeated once to determine eligibility and the result must be available prior to Visit 2 (Day 1). Screening visit (Visit 1) activities may be extended to two physical visits if needed.

Prior liver biopsy or fibroscan results will be recorded in the eCRF as medical history.

Patients who fail screening following Visit 1 procedures should be registered as a screen failure in IRT.

6.2.2 Treatment period

The treatment period is from Visit 2 to EOT Visit. Patients will be dispensed medication at visits according to the Flow Chart and new medication unit number(s) will be provided through the IRT on each occasion.

Instruct patient NOT to take study medication on the morning of all visits.

Proprietary confidential information.
© 2014 Boehringer Ingelheim International GmbH or one or more of its affiliated companies. All rights reserved.
This document may not - in full or in part - be passed on, reproduced, published or otherwise used without prior written permission.

Proprietary confidential information © 2015 Boehringer Ingelheim International GmbH or one or more of its affiliated companies

Administration of the morning dose of study drug will be done under the supervision of the investigator or his designee after the pre-dose PK sample has been collected. Note that the routine laboratory samples are to be collected in fasted condition and only afterwards patients must take their medication with food.

6.2.2.1 Randomization/treatment assignment visit (Visit 2)

Patients will begin treatment as assigned by the IRT at Visit 2 with the first dose administered in the office (Day 1) including an additional 120 mg loading dose of FDV ~~(or placebo)~~ (cf. Section 4.1.4).

~~For Cohort B, IRT will assign mandatory PK sub-study participation (cf. Section 5.5).~~

~~6.2.2.2 Visit 3 to Visit 12~~

~~For Cohort B, results of plasma HCV RNA levels and a series of safety laboratory tests after start of the treatment will be blinded to investigators, patients and sponsor (cf. Section 4.1.5.1). The Investigator will be informed by the central laboratory when a criteria for treatment discontinuation due to lack of virologic response (cf. Section 3.3.4.1) are met for a given patient during this period.~~

6.2.2.~~3~~2 EOT Visit

Planned EOT visit will occur at Week 24 according to the planned treatment duration. These patients should be registered as completed in IRT.

Patients who discontinue treatment early due to either lack of efficacy or safety reasons will be required to have an EOT visit (if the patient is at the research site), or have EOT visit scheduled within 2 weeks from the last intake of study medication. These patients should be registered in IRT as early discontinuation of trial medication. Please note that if either BI 207127 or FDV is permanently discontinued, all study medication including RBV must be discontinued immediately (cf. Section 4.2.1).

~~For Cohort B, the sponsor, patient, and investigator will be unblinded to all laboratory values and treatment (active or placebo) for each patient at their EOT visit.~~

6.2.3 End of trial and follow-up period

Termination of trial medication and trial completion must be recorded on the corresponding eCRFs for all randomised/treatment assigned patients.

6.2.3.1 Follow-up (FU)

If HCV RNA is detected during a FU visit, a subsequent confirmatory testing should be performed.

If a patient receives any other HCV treatment outside this protocol during the FU period the patient will need to be discontinued from the trial and have EOO visit prior to the start of that

Proprietary confidential information.
© 2014 Boehringer Ingelheim International GmbH or one or more of its affiliated companies. All rights reserved.
This document may not - in full or in part - be passed on, reproduced, published or otherwise used without prior written permission.

Proprietary confidential information © 2015 Boehringer Ingelheim International GmbH or one or more of its affiliated companies

treatment. Efficacy data collected after the start of such treatment will not be attributed to the trial treatment. Any subsequent end-points will be considered as not achieving treatment response.

Patients with EOT at Week 24

Patients who successfully complete the 24-week treatment period should return to the clinic for FU visits according to the Flow Chart. FU1 visit will occur 4 weeks after the discontinuation of trial treatment. This is the last at which the investigator has the responsibility to report every AE. During the next FU visits, only SAEs will be reported. Refer to Flow Chart and Section 5.2.2.

Patient with early treatment termination

Patients who stop study treatment prior to completion of the treatment period should return to the clinic for all FU visits per the Flow Chart starting with a FU1 visit 4 weeks after EOT.

It is understood that a patient may withdraw consent at any time and decide not to complete FU, but such FU is to be encouraged.

6.2.3.2 End of Observation

EOO will be performed for all patients at ~~96~~ **12** weeks after EOT.

Patients who discontinue the FU period early will be required to have an EOO visit (if the patient is at the research site), or have EOO visit scheduled within 3 weeks from discontinuation of the FU.

~~6.2.3.3 Treatment Option for Arm 4~~

~~For patients that participated in Arm 4, an option to received trial treatment according to Arm 5 may be offered, if patient is still eligible for treatment (cf. Section 3.1). The trial Flow Chart will not be followed; instead these patients will follow the treatment visit schedule and procedures as listed in Table 4.2.1: 2. Refer to ISF for further details on the procedures to follow and unblinding.~~

Proprietary confidential information.
© 2014 Boehringer Ingelheim International GmbH or one or more of its affiliated companies. All rights reserved.
This document may not - in full or in part - be passed on, reproduced, published or otherwise used without prior written permission.

Proprietary confidential information © 2015 Boehringer Ingelheim International GmbH or one or more of its affiliated companies

7. STATISTICAL METHODS AND DETERMINATION OF SAMPLE SIZE

7.1 STATISTICAL DESIGN - MODEL

7.1.1 Design

Cohort A:

This design is a multi-national, ~~three~~ **two**-arm, open label trial. Fifteen CPA patients will be treated with 600 mg BI 207127 b.i.d. in combination with 120 mg FDV q.d. and RBV (Arm 1); 15 CPB patients will be treated with 400 mg BI 207127 b.i.d. in combination with 120 mg FDV q.d. and RBV (Arm 2).~~; If patients in Arm 2 do not reach the target exposure, Arm 3 of 15 CPB patients treated with 600 mg BI 207127 b.i.d. in combination with 120 mg FDV q.d. and RBV.~~

~~Cohort B:~~

~~This design is a multi-national, randomised, double-blind, placebo controlled, parallel-group trial (cf. Section 3.1). Moderate hepatic impairment (CPB) patients with HCV genotype 1b infection will be randomised in a 1:3 ratio to placebo:active treatment.~~

7.2 NULL AND ALTERNATIVE HYPOTHESES

Cohort A:

No hypothesis testing will be performed. The results will be presented descriptively.

~~Cohort B:~~

~~The null hypothesis is that the incidence of SVR12 in the active treated group is equal to that in the placebo group. The alternative hypothesis is that the two incidences are unequal, with the goal to demonstrate a higher SVR12 rate in the active-treatment group than in the placebo group. The hypothesis will be tested using alpha=0.05 (2-sided) with at least 80% of power.~~

7.3 PLANNED ANALYSES

The primary analyses of efficacy will be carried out on an intent-to-treat basis including all patients who are randomised and receive at least one dose of assigned therapy during the trial.

~~The interim analysis for Cohort A to determine BI 207127 dose in Cohort B will be performed after all Arm 1 and 2 patient reach Week 4 and, if needed, after all Arm 3 patients reach Week 4.~~

~~The primary analysis for Cohort A and B will be performed after all patients complete FU2 (assessment of SVR12, the primary efficacy endpoint).The final analysis~~ **All analyses** will be performed after all patients complete their last planned visit~~, including data from Cohort B placebo arm patients that received active treatment~~.

Proprietary confidential information.
© 2014 Boehringer Ingelheim International GmbH or one or more of its affiliated companies. All rights reserved.
This document may not - in full or in part - be passed on, reproduced, published or otherwise used without prior written permission.

Proprietary confidential information © 2015 Boehringer Ingelheim International GmbH or one or more of its affiliated companies

7.3.1 Primary analyses

Cohort A:

For the primary endpoint SVR12, ninety-five percent confidence intervals (CIs) will be presented.

PK analysis will be performed after Day 1, 8, **and** Week 4 ~~and 16~~ intensive PK sample collection for Cohort A. For details of the PK **endpoints** ~~analysis~~, see Section 5.5.1 ~~7.3.4~~.

~~Cohort B:~~

~~For the primary analysis, Fisher's exact test will be used for the hypothesis testing.~~

~~SVR12 will be assessed based on the observed HCV RNA result taken at least 8 weeks (to allow an appropriate visit window) after treatment discontinuation. This definition will also hold for patients that discontinue treatment early, i.e. if the patient has HCV RNA undetected at least 8 weeks after stop of all treatment, they will be considered a responder in the primary analysis.~~

~~If a patient has detectable HCV RNA at the FUP2 visit but had undetectable HCV RNA at the previous visit, a confirmation sample will be used to determine SVR12. This procedure will also be implemented for patients who have multiple observations in the SVR12 window (≥10 weeks after the planned treatment period) – that is, if a patient has a detectable HCV RNA after being undetectable, confirmation will be required before classifying the patient as a SVR12 failure. Patients that take other HCV therapies after stopping all trial medication will also be considered SVR12 failure in the primary analyses.~~

7.3.2 Secondary analyses

~~Sensitivity analyses of the primary endpoint will be performed for the per-protocol set and completers set to assess the impact of important protocol violations and premature discontinuation on the primary endpoint.~~

~~A further sensitivity analysis will be performed imputing missing SVR data by last available post-treatment result (SVR12).~~

For ~~T~~**t**he secondary endpoints SVR4 and SVR24**,** ~~will be evaluated with the same statistical method that is described for the primary endpoint in Section 7.3.1~~ **ninety-five percent CIs will be presented**.

~~Ninety-five percent confidence intervals (CIs) will be reported for the differences in response rates between treatment groups for SVR12, SVR4, and SVR24.~~

7.3.3 Safety analyses

All safety data will be displayed and analysed using descriptive statistical methods. No formal inferential analysis is planned for safety comparison

Proprietary confidential information.
© 2014 Boehringer Ingelheim International GmbH or one or more of its affiliated companies. All rights reserved.
This document may not - in full or in part - be passed on, reproduced, published or otherwise used without prior written permission.

Proprietary confidential information © 2015 Boehringer Ingelheim International GmbH or one or more of its affiliated companies

AEs will be coded using the Medical Dictionary for Drug Regulatory Activities (MedDRA) coding dictionary. All events with an onset after the first dose of study medication up to a period of 28 days after the last dose of study medication will be assigned to the treatment phase for evaluation. Other AEs will be assigned either to the screening or FU phase as appropriate. Frequency tables of patients who experience AEs leading to discontinuation will be provided. Kaplan-Meier curves might be used to investigate the time to an event for considerations of special interest, for example rash event. More details of these analyses will be included in the TSAP.

Laboratory values taken after the first dose of randomised treatment up to a period of 7 days after the last intake of treatment will be assigned to the treatment phase for evaluation. Frequency tables with abnormal laboratory values defined by DAIDS grades (R10-1332) and, if necessary, the proportion exceeding multiples (2*, 3* …) of the upper normal range limit will be provided. Changes in bilirubin (direct, indirect, total) values will be described in the context of being associated with clinical events. The absolute value and the change from baseline in laboratory values will be summarized by treatment group. Baseline is defined as the last observed measurement prior to administration of any randomised study medication.

A DMC will be set up with primary responsibility to review the safety database on an ongoing basis. Refer to Section 3.1.1 for more details.

7.3.4 Interim analyses

For safety monitoring, trough plasma concentrations of BI 207127 and FDV up to Week 4 will be analysed for Arm 1 and Arm 2 patients after all patients have completed visit 6 and, as needed, until all patients have completed EOT. Geometric mean of difference, sd, 90% CI will be calculated between treatment arms. ~~For Cohort A, PK parameters AUC_{0-12}, Cmax, C_{12} of BI 207127 and AUC_{0-24}, Cmax, C_{24} of FDV will be calculated and provided to evaluate the systemic exposure of BI 207127 and FDV in CPB patients after receiving 400 mg BI 207127 compared to BI 207127 and FDV in CPA patients after receiving 600 mg BI 207127. Geometric mean of difference, sd, 90% CI will be calculated for these parameters between treatment arms. The results will be presented descriptively and no statistical testing will be performed. The lower bound of the 90% CI for the geometric mean test arm (Arm 1): control arm (Arm 2) of C_{12} will be compared to 0.5 while the upper bound will be compared to 2. The boundaries were derived based on SOUND-C2 results. Whether the lower BI 207127 dose treatment in Arm 1 will provide proper exposure to CPB patients will be decided by assessment of the descriptive statistics listed above along with the efficacy and safety profile of the patients by an independent internal review committee (cf. Section 3.1.1). This committee will either select a dose for Cohort B or determine that Arm 3 should be conducted and the same analysis performed comparing Arm 1 to Arm 3.~~

Proprietary confidential information.
© 2014 Boehringer Ingelheim International GmbH or one or more of its affiliated companies. All rights reserved.
This document may not - in full or in part - be passed on, reproduced, published or otherwise used without prior written permission.

Proprietary confidential information © 2015 Boehringer Ingelheim International GmbH or one or more of its affiliated companies

7.3.5 Pharmacokinetic analyses

Cohort A:

Refer to Section 5.5.1 ~~7.3.4~~.

~~Cohort B:~~

~~The PK parameters AUC_{0-12}, C_{max}, C_{12} of BI 207127 and AUC_{0-24}, C_{max}, C_{24} of FDV will be calculated after Day 1, 8, Week 4 and 16 of intensive PK sample collection.~~

7.3.6 Other analyses

Ninety-five percent CIs will be reported for the differences in response rates between treatment groups for $W4U_{TND}$, W4U, $W12U_{TND}$, and ETR_{TND}.

Time to achieving HCV RNA undetected and time to virologic failure will be analyzed using Cox model.

~~The relationship between patient response and exposure will be investigated. HCV RNA change from baseline over time will be regressed on corresponding BI 201335 and BI 207127 concentrations to assess saturation of the exposure-response relationship.~~

Biochemical response and liver disease progression will be presented descriptively.

7.4 HANDLING OF MISSING DATA

All randomised patients who discontinue from the trial without reaching the SVR time points will be counted as treatment failures. Patients with missing SVR4 will be imputed by SVR12, if available. Patients with missing SVR12 will be imputed by SVR24, if available. Likewise, patients with missing SVR24 will be left as missing at the time of the interim database lock. Sensitivity analyses for missing SVR12 values due to early discontinued patients will be described in the TSAP.

It is not planned to impute missing values for safety data with the exception that missing AE start and end dates will be estimated (details to be written up in the TSAP).

7.5 RANDOMISATION

Cohort A:

Eligible patients will be assigned to a treatment arm according the patient's Child Pugh score and the current screening stage of the trial. No randomisation is needed. All treatment arms will require a minimum of 8 females per arm.

~~Cohort B:~~

Proprietary confidential information.
© 2014 Boehringer Ingelheim International GmbH or one or more of its affiliated companies. All rights reserved.
This document may not - in full or in part - be passed on, reproduced, published or otherwise used without prior written permission.

Proprietary confidential information © 2015 Boehringer Ingelheim International GmbH or one or more of its affiliated companies

~~Eligible patients will be randomly assigned to one of the two treatment regimens to avoid possible biases due to subjective patient selection. An allocation ratio of 1:3 for Arm 4 and Arm 5 is planned.~~

~~The randomisation list will be generated using a validated system, which involves a pseudorandom number generator to guarantee the reproducibility of the assignments. This randomisation list will be checked by an independent statistician and used by a third-party IRT system to assign randomisation numbers to eligible patients.~~

7.6 DETERMINATION OF SAMPLE SIZE

Cohort A:

Aiming at equal trough between Arms 1 and 2, 15 patients per arm will provide reasonable power to detect differences when alpha = 0.1 and the lower equivalence bound of the trough ratio is set at 0.5 and upper bound set at 2 (Table 7.6: 1).

Table 7.6: 1 Power calculation for Cohort A (alpha = 0.1)

CV	Power
80%	83%
100%	67%
120%	52%

Computed with proc power of SAS version 9.2 (assuming log normal and using exact method)

~~Cohort B:~~

~~Thirty patients will be recruited for the placebo group and 90 will be recruited for the active treatment group. The response rate for the placebo group is expected to be zero. For calculation purpose, it is set to 1%. The reference response rate for the active treatment group has not been set yet because no such treatment combination was tested on such patient population. The trial will have a power of 81% to detect, with a 2-sided alpha=0.05, an SVR12 incidence rate of 20% on active treatment group using Fisher's exact test. If the incidence rate reaches 30%, the power will be greater than 99% (Table 7.6: 2).~~

~~Table 7.6: 2 Power calculation for Cohort B (incidence rate in placebo group is set at 1%, 2-sided alpha = 0.05)~~

~~Power~~	~~Incidence rate of active treatment group~~

Proprietary confidential information.
© 2014 Boehringer Ingelheim International GmbH or one or more of its affiliated companies. All rights reserved.
This document may not - in full or in part - be passed on, reproduced, published or otherwise used without prior written permission.

Proprietary confidential information © 2015 Boehringer Ingelheim International GmbH or one or more of its affiliated companies

~~81%~~	~~20%~~
~~95%~~	~~25%~~
~~>99%~~	~~30%~~

~~Computed with NQuery version 6.01(routine PTT2U)~~

Proprietary confidential information.
© 2014 Boehringer Ingelheim International GmbH or one or more of its affiliated companies. All rights reserved.
This document may not - in full or in part - be passed on, reproduced, published or otherwise used without prior written permission.

Proprietary confidential information © 2015 Boehringer Ingelheim International GmbH or one or more of its affiliated companies

8. INFORMED CONSENT, DATA PROTECTION, TRIAL RECORDS

The trial will be carried out in compliance with the protocol, the principles laid down in the Declaration of Helsinki, in accordance with the ICH Harmonised Tripartite Guideline for GCP and relevant BI SOPs. Standard medical care (prophylactic, diagnostic and therapeutic procedures) remains in the responsibility of the treating physician of the patient.

The investigator should inform the sponsor immediately of any urgent safety measures taken to protect the study subjects against any immediate hazard, and also of any serious breaches of the protocol/ICH GCP.

The rights of the investigator and of the sponsor with regard to publication of the results of this trial are described in the investigator contract. As a general rule, no trial results should be published prior to finalisation of the CTR.

Insurance Cover: The terms and conditions of the insurance cover are made available to the investigator and the patients via documentation in the ISF.

8.1 STUDY APPROVAL, PATIENT INFORMATION, AND INFORMED CONSENT

This trial will be initiated only after all required legal documentation has been reviewed and approved by the respective IRB / IEC and CA according to national and international regulations. The same applies for the implementation of changes introduced by amendments.

Prior to patient participation in the trial, written informed consent must be obtained from each patient (or the patient's legally accepted representative) according to ICH GCP and to the regulatory and legal requirements of the participating country. Each signature must be personally dated by each signatory and the informed consent and any additional patient-information form retained by the investigator as part of the trial records. A signed copy of the informed consent and any additional patient information must be given to each patient or the patient's legally accepted representative.

The patient must be informed that his/her personal trial-related data will be used by Boehringer Ingelheim in accordance with the local data protection law. The level of disclosure must also be explained to the patient.

The patient must be informed that his / her medical records may be examined by authorised monitors (CML/CRA) or Clinical Quality Assurance auditors appointed by Boehringer Ingelheim, by appropriate IRB / IEC members, and by inspectors from regulatory authorities.

8.2 DATA QUALITY ASSURANCE

A quality assurance audit/inspection of this trial may be conducted by the sponsor or sponsor's designees or by IRBs / IECs or by regulatory authorities. The quality assurance auditor will have access to all medical records, the investigator's trial-related files and correspondence, and the informed consent documentation of this clinical trial.

Proprietary confidential information.
© 2014 Boehringer Ingelheim International GmbH or one or more of its affiliated companies. All rights reserved.
This document may not - in full or in part - be passed on, reproduced, published or otherwise used without prior written permission.

Proprietary confidential information © 2015 Boehringer Ingelheim International GmbH or one or more of its affiliated companies

8.3 RECORDS

Case Report Forms (CRFs) for individual patients will be provided by the sponsor, either on paper or via remote data capture (RDC). See Section 4.1.5.2 for rules about emergency code breaks. For drug accountability, refer to Section 4.1.8.

8.3.1 Source documents

Source documents provide evidence for the existence of the patient and substantiate the integrity of the data collected. Source documents are filed at the investigator's site.

Data entered in the eCRFs that are transcribed from source documents must be consistent with the source documents or the discrepancies must be explained. The investigator may need to request previous medical records or transfer records, depending on the trial; also current medical records must be available.

For eCRFs all data must be derived from source documents.

8.3.2 Direct access to source data and documents

The investigator / institution will permit trial-related monitoring, audits, IRB / IEC review and regulatory inspection, providing direct access to all related source data / documents. CRFs/eCRFs and all source documents, including progress notes and copies of laboratory and medical test results must be available at all times for review by the sponsor's clinical trial monitor, auditor and inspection by health authorities (e.g. FDA). The CRA / on site monitor and auditor may review all CRFs/eCRFs, and written informed consents. The accuracy of the data will be verified by reviewing the documents described in Section 8.3.1.

8.4 LISTEDNESS AND EXPEDITED REPORTING OF ADVERSE EVENTS

8.4.1 Listedness

To fulfill the regulatory requirements for expedited safety reporting, the sponsor evaluates whether a particular AE is "listed", i.e. is a known side effect of the drug or not. Therefore a unique reference document for the evaluation of listedness needs to be provided. For the BI 207127 and FDV this is the current version of the IB (U06-1740 and U04-3332). For RBV this is EU SmPC. The current versions of these reference documents are to be provided in the ISF. No AEs are classified as listed for matching placebo, study design, or invasive procedures.

8.4.2 Expedited reporting to health authorities and IECs / IRBs

Expedited reporting of SAEs, e.g. SUSARs, to health authorities and IECs / IRBs, will be done according to local regulatory requirements. Further details regarding this reporting procedure are provided in the ISF.

Proprietary confidential information.
© 2014 Boehringer Ingelheim International GmbH or one or more of its affiliated companies. All rights reserved.
This document may not - in full or in part - be passed on, reproduced, published or otherwise used without prior written permission.

Proprietary confidential information © 2015 Boehringer Ingelheim International GmbH or one or more of its affiliated companies

8.5 STATEMENT OF CONFIDENTIALITY

Individual patient medical information obtained as a result of this trial is considered confidential and disclosure to third parties is prohibited with the exceptions noted below. Patient confidentiality will be ensured by using patient identification code numbers.

Treatment data may be given to the patient's personal physician or to other appropriate medical personnel responsible for the patient's welfare. Data generated as a result of the trial need to be available for inspection on request by the participating physicians, the sponsor's representatives, by the IRB / IEC and the regulatory authorities.

Proprietary confidential information.
© 2014 Boehringer Ingelheim International GmbH or one or more of its affiliated companies. All rights reserved.
This document may not - in full or in part - be passed on, reproduced, published or otherwise used without prior written permission.

Proprietary confidential information © 2015 Boehringer Ingelheim International GmbH or one or more of its affiliated companies

9. REFERENCES

9.1 PUBLISHED REFERENCES

P97-2059 Fattovich G, et al. Morbidity and mortality in compensated cirrhosis type C: a retrospective follow-up study of 384 patients. Gastroenterology 1997;112(2):463-472.

R99-1243 Pugh RNH, Murray-Lyon IM, Dawson JL, Pietroni MC, Williams R. Transsection of the oesophagus for bleeding oesophageal varices. Br J Surg 1973;60(8):646-649.

P00-15027 Heathcote J. Antiviral therapy for patients with chronic hepatitis C. Semin Liver Dis. 2000. 20(2): 185-199.

R10-1332 Division of AIDS Regulatory Support Center (DAIDS RSC) Division of AIDS table for grading the severity of adult and pediatric adverse events (version 1.0, December, 2004 (clarification August 2009)). http://rsc.tech-res.com/safetyandpharmacovigilance/ (accessed June 22, 2010) (2009).

R10-2440 Child CG, Turcotte JG. Surgery and portal hypertension. In: Child CG, editor. The liver and portal hypertension. Philadelphia: Saunders; 1964. p. 50-64.

R12-0262 Incivek (telaprevir) film coated tablets (Vertex Pharmaceuticals) (US prescribing information, revised: 5/2011). 2011

R12-0263 Victrelis (boceprevir) capsules (Merck & Co) (US prescribing information, revised: 05/2011). 2011

9.2 UNPUBLISHED REFERENCES

U04-3332 Steinmann G. Investigator's Brochure FDV NA, Chronic Hepatitis C Infection, 1220.P1. Current Version 12 10 Oct 2011.

U06-1740 Kukolj G, Boecher W, Wu K, Stachlewitz R, Sane R, Hinderliter J. Investigator´s Brochure BI 207127 NA, Chronic Hepatitis C Virus Infection 1241.P1. Current Version 7. 08 Dec 2011.

U11-3471-01 Mensa F, Nehmiz G, Sabo J. Summary report of interim analysis at Week 12, Trial 1241.21 Part 2. 12 Sep 2011.

U11-3472-01 Sabo J, Lehr T, Mensa F. Clinical pharmacology report. Trial 1241.21 Part 1. Snapshot archive version No. 1.4. 7 Dec 2011.

Proprietary confidential information.
© 2014 Boehringer Ingelheim International GmbH or one or more of its affiliated companies. All rights reserved.
This document may not - in full or in part - be passed on, reproduced, published or otherwise used without prior written permission.

Proprietary confidential information © 2015 Boehringer Ingelheim International GmbH or one or more of its affiliated companies

10. APPENDICES

10.1 PHARMACOKINETIC METHODS

10.1.1 Evaluation of Pharmacokinetic Parameters

For the calculation of PK parameters, only concentrations within the validated concentration range will be used. The actual sampling times will be used. For pre-dose samples, the actual sampling time will be set to zero.

Noncompartmental PK parameters will be determined using WinNonlin or another validated program.

C_{max} and t_{max}: Individual C_{max} and t_{max} values will be directly determined from the plasma concentration time profiles of each subject. If the same C_{max} concentration occurs at different time points, t_{max} is assigned to the first occurrence of C_{max}.

AUCs**:** The areas under the curve spanning various time intervals will be calculated using the linear up/log down algorithm. If a drug concentration is equal to or higher than the preceding concentration, the linear trapezoidal method will be used. If the drug concentration is smaller than the preceding concentration, the logarithmic method will be used.

Linear trapezoidal rule ($t_2 > t_1$ and $C_2 \geq C_1$):

The area of the trapezoid between the two data points (t_1, C_1) and (t_2, C_2) will be computed by:

$$AUC_{t1\text{-}t2} = 0.5 \times (t_2 - t_1) \times (C_1 + C_2)$$

Logarithmic trapezoid rule ($t_2 > t_1$ and $C_2 < C_1$):

The area of the trapezoid between the two data points (t_1, C_1) and (t_2, C_2) will be computed by:

$$AUC_{t1-t2} = \frac{(t_2 - t_1) \times (C_2 - C_1)}{\ln(C_2 / C_1)}$$

~~Estimation of λ_z: The apparent terminal rate constant λ_z will be estimated from a regression of ln(C) versus time over the terminal log-linear drug disposition portion of the concentration-time profiles. The log-linear profiles, which include the regression line through the terminal points, will be checked via visual inspection, and it will be determined whether the regression appropriately represents the terminal slope. Only data points that describe the terminal log-linear decline will be included in the regression. A minimum of three points will be used in the determination of λ_z. If the last concentration-time point increases, this time point may be included if the $t_{1/2}$ estimate is reasonable. If λ_z is not determinable then consequently only parameters not requiring λ_z will be reported. In addition, the lower ($t_{\lambda z,start}$) and upper ($t_{\lambda z,end}$) limit on time for values to be included in the calculation of λ_z will be listed.~~

Proprietary confidential information.
© 2014 Boehringer Ingelheim International GmbH or one or more of its affiliated companies. All rights reserved.
This document may not - in full or in part - be passed on, reproduced, published or otherwise used without prior written permission.

Proprietary confidential information © 2015 Boehringer Ingelheim International GmbH or one or more of its affiliated companies

~~$t_{1/2}$: The terminal half-life will be calculated from the terminal rate constant using the equation:~~

$$t_{1/2} = \frac{\ln 2}{\lambda_z}$$

~~MRT_{po}: The mean residence time after extravascular administration (MRT_{po}) will be calculated as follows:~~

$$MRT_{po} = \frac{AUMC_{0-\infty}}{AUC_{0-\infty}}$$

~~The area under the first moment curve from time 0 to infinity ($AUMC_{0-\infty}$) is calculated according to:~~

$$AUMC_{0-\infty} = AUMC_{0-tz} + \frac{C'_{tz} \times t_z}{\lambda_z} + \frac{C'_{tz}}{\lambda_z^2}$$

~~CL/F: The apparent clearance after extravascular administration will be determined according to the following equation:~~

$$CL \text{ or } CL/F = \frac{Dose}{AUC_{0-\infty}}$$

~~(F = absolute bioavailability factor)~~

~~V_z/F: The apparent volume of distribution during the terminal phase after extravascular administration (at steady state) will be determined according to the following equation:~~

$$V_z/F = \frac{CL/F}{\lambda_z}$$

The geometric mean (gMean) and coefficient of variation, gCV (given in %), will be calculated by the formulae:

$$gMean = \exp\left[\tfrac{1}{n}\sum_{i=1}^{n}\ln(x_i)\right] = \exp\left[\overline{\ln(x_i)}\right]$$

$$gCV(\%) = 100 \cdot \sqrt{\exp\left[Var(\ln(x_i))\right] - 1}$$

where

$$Var(\ln(x_i)) = \frac{1}{n-1}\sum_{i=1}^{n}\left[\ln(x_i) - \overline{\ln(x_i)}\right]^2$$

Proprietary confidential information.
© 2014 Boehringer Ingelheim International GmbH or one or more of its affiliated companies. All rights reserved.
This document may not - in full or in part - be passed on, reproduced, published or otherwise used without prior written permission.

Proprietary confidential information © 2015 Boehringer Ingelheim International GmbH or one or more of its affiliated companies

10.1.2 Blood sampling scheme for Pharmacokinetics

Table 10.1.2: 1 Blood sampling scheme for intensive PK at Day 1, 8 and Week 4 ~~and 16~~ in Cohort A (Arms 1, 2 ~~and 3~~)

Visit	Planned Time [h:min]	Clock Time (of the actual day) [h:min]	PK Plasma Sample	~~Protein Binding sample~~	Administration of the study drug
2, 3, 6, ~~11~~	-0:05	07:55	X[2]		
	0:00	08:00			X[1]
	0:30	08:30	X		
	1:00	09:00	X		
	1:30	09:30	X		
	2:00	10:00	X		
	2:30	10:30		~~X~~	
	3:00	11:00	X		
	4:00	12:00	X		
	5:00	13:00	X		
	6:00	14:00	X		
	8:00	16:00	X		
	10:00	18:00	X		
	12:00	20:00	X		
	24:00	08:00	X		

Footnotes:

[1] Administration of the study drug will be done under the supervision of the investigator or his designee after the pre-dose sample has been collected. The time elapsed since the last dose of FDV should be as close to 24 hours as possible and the last dose of BI 207127 should be as close to 12 hours as possible.

[2] Pre-dose blood sample is collected within 10 minutes prior to the first drug administration of the day (nominal time 08:00).

Proprietary confidential information.
© 2014 Boehringer Ingelheim International GmbH or one or more of its affiliated companies. All rights reserved.
This document may not - in full or in part - be passed on, reproduced, published or otherwise used without prior written permission.

Proprietary confidential information © 2015 Boehringer Ingelheim International GmbH or one or more of its affiliated companies

~~Table 10.1.2: 2 Blood sampling scheme for PK sub-study visits in Cohort B (Arm 4 and Arm 5)~~

~~Patient month of birth~~[1]	~~Blood sample collection time window~~[2] ~~(hours relative to first drug administration of the day)~~[3]				
	~~Sample 1~~[4]	~~Sample 2~~[5]	~~Sample 3~~[5]	~~Sample 4~~[5]	~~Sample 5~~[5]
~~January~~	~~-0.05 (pre-dose)~~	~~0.5 – 1.5~~	~~3 – 4~~	~~5.5 – 6.5~~	~~8 – 9~~
~~February~~	~~-0.05 (pre-dose)~~	~~1 – 2~~	~~3.5 – 4.5~~	~~6 – 7~~	~~8.5 – 9.5~~
~~March~~	~~-0.05 (pre-dose)~~	~~1.5 – 2.5~~	~~4 – 5~~	~~6.5 – 7.5~~	~~9 – 10~~
~~April~~	~~-0.05 (pre-dose)~~	~~0.5 – 1.5~~	~~3 – 4~~	~~5.5 – 6.5~~	~~8 – 9~~
~~May~~	~~-0.05 (pre-dose)~~	~~1 – 2~~	~~3.5 – 4.5~~	~~6 – 7~~	~~8.5 – 9.5~~
~~June~~	~~-0.05 (pre-dose)~~	~~1.5 – 2.5~~	~~4 – 5~~	~~6.5 – 7.5~~	~~9 – 10~~
~~July~~	~~-0.05 (pre-dose)~~	~~0.5 – 1.5~~	~~3 – 4~~	~~5.5 – 6.5~~	~~8 – 9~~
~~August~~	~~-0.05 (pre-dose)~~	~~1 – 2~~	~~3.5 – 4.5~~	~~6 – 7~~	~~8.5 – 9.5~~
~~September~~	~~-0.05 (pre-dose)~~	~~1.5 – 2.5~~	~~4 – 5~~	~~6.5 – 7.5~~	~~9 – 10~~
~~October~~	~~-0.05 (pre-dose)~~	~~0.5 – 1.5~~	~~3 – 4~~	~~5.5 – 6.5~~	~~8 – 9~~
~~November~~	~~-0.05 (pre-dose)~~	~~1 – 2~~	~~3.5 – 4.5~~	~~6 – 7~~	~~8.5 – 9.5~~
~~December~~	~~-0.05 (pre-dose)~~	~~1.5 – 2.5~~	~~4 – 5~~	~~6.5 – 7.5~~	~~9 – 10~~

~~**Footnotes:**~~

1 ~~Pseudo-randomised scheme based on patient's birth month. PK samples are collected at different time windows depending on birth month of patient as specified in the table above.~~

2 ~~For collection of PK sub-study samples, patients will be assignment via IRT at Visit 2 to one of the following collection schedules: Day 1 and Day 8; Day 1 and Week 4; or Day 1 and Week 16.~~

3 ~~Administration of the study drug will be done under the supervision of the investigator or his designee after the pre-dose sample has been collected. The time elapsed since the last dose of FDV should be as close to 24 hours as possible and the last dose of BI 207127 should be as close to 12 hours as possible.~~

4 ~~Pre-dose blood sample (Sample 1) is collected within 10 minutes prior to the first drug administration of the day (nominal time 08:00).~~

5 ~~Post-dose blood samples (Sample 2 to Sample 5) are collected at any time within the collection window and are relative to the first drug administration of the day (nominal time 08:00).~~

Proprietary confidential information.
© 2014 Boehringer Ingelheim International GmbH or one or more of its affiliated companies. All rights reserved.
This document may not - in full or in part - be passed on, reproduced, published or otherwise used without prior written permission.

Proprietary confidential information © 2015 Boehringer Ingelheim International GmbH or one or more of its affiliated companies

10.2 DERMATOLOGY MANAGEMENT PLAN

10.2.1 Introduction and scope

Drug-induced skin reactions (rash and photosensitivity reactions) have been reported with the use of FDV and BI 207127 combination therapy. The aim of this plan is to provide guidance for the management of suspected or confirmed drug-induced skin reactions. This section is not intended to provide guidance for skin events with known aetiologies in which a drug-relatedness has been ruled out (e.g. measles, skin cancer, scabies, etc).

For the management of skin rash cases refer to Section 10.2.3 and of photosensitivity reactions refer to Section 10.2.4. Skin rash cases may be reported as rash, or other specific diagnoses, e.g. urticaria, exanthema, etc. Photosensitivity reactions may be reported as phototoxic reaction, sunburn, etc.

10.2.2 General measures for skin protection

Patients should be instructed to protect any uncovered skin area (including hands, face and lips) from sun- or UV-light exposure using sun-blocker cream and lip balm with an SPF ≥50 providing UV-A and -B protection during treatment period on a daily basis. Patients should avoid unnecessary or prolonged exposure to sunlight and wear protective clothing, sunglasses and hats in addition to sun-block. It is recommended to avoid direct sun exposure as much as possible. Tanning booths must be avoided during treatment period.

All patients should be instructed to report all skin reactions and be seen by study medical staff as soon as possible. If a patient has photosensitivity reaction, the patient should be instructed to be even more consistent with the measures described earlier and stay away from windows as much as possible.

10.2.3 Drug-induced skin rash

These skin reactions may manifest with a multitude of clinical presentations and variable severity, ranging from mild rashes to life-threatening reactions such as DRESS, Stevens-Johnson Syndrome (SJS), toxic epidermal necrolysis (TEN) or acute generalized exanthematous pustulosis (AGEP). Patients need to be monitored for the appearance of systemic symptoms such as persistent fever, lymphadenopathy and/or involvement of mucous membranes and internal organs.

10.2.3.1 Definition of severity for skin rash*

* definition of severity based on the DAIDS grading of rash (Division of AIDS Table for Grading the Severity of Adult and Pediatric Adverse Events), version 1.0 as of December 2004 – Clarification AUGUST 2009.

Mild (Grade I):

Localized macular or maculopapular rash.

Moderate (Grade II):

Proprietary confidential information.
© 2014 Boehringer Ingelheim International GmbH or one or more of its affiliated companies. All rights reserved.
This document may not - in full or in part - be passed on, reproduced, published or otherwise used without prior written permission.

Proprietary confidential information © 2015 Boehringer Ingelheim International GmbH or one or more of its affiliated companies

Diffuse macular, maculopapular, or morbilliform rash; or, target lesions.

Severe (Grade III):

Diffuse macular, maculopapular, or morbilliform rash with vesicles; limited number of bullae; or, superficial ulcerations of mucous membrane limited to one site.

Potentially Life-Threatening (Grade IV):

Extensive or generalized bullous lesions; SJS; ulceration of mucous membrane involving two or more distinct mucosal sites; or, TEN. Note that DRESS is not listed in DAIDs grading table but is considered potentially life threatening.

If a severe or potentially life-threatening cutaneous event is suspected, it is important that a dermatologist who is experienced in these reactions should be promptly consulted to assist in the diagnosis and management of the patient.

10.2.3.2 Management guidelines of skin rash

Mild:

Medium to high potency topical corticosteroids (potentially combined with oral antihistamines) should be prescribed as early as possible. Other non-prescription remedies such as oatmeal baths may be used to provide temporary relief of skin sensitivity and pruritus.

The need for UV light protection should be reinforced (Section 10.2.2).

Treatment with study medication may be continued without interruption.

Moderate:

The patient should have a detailed photo-documentation of the skin, preferably prior to the administration of any therapy. The subject should be seen by a dermatologist as soon as possible, but no later than 72 hours for diagnosis, confirmation of severity and treatment. A non-progressive moderate skin rash, confirmed by the dermatologist should be closely and frequently monitored at the investigator's discretion taking into account recommendation by dermatologist.

Medium to high potency topical corticosteroids combined with oral antihistamines should be prescribed as early as possible, but preferably after detailed photo-documentation of the skin lesions. Other non-prescription remedies such as oatmeal baths may be used to provide temporary relief of skin sensitivity and pruritus. Systemic corticosteroids may be applied if deemed clinically necessary.

The need for UV light protection should be reinforced (Section 10.2.2).

These reactions do not require discontinuation of study treatment.

Severe:

Proprietary confidential information.
© 2014 Boehringer Ingelheim International GmbH or one or more of its affiliated companies. All rights reserved.
This document may not - in full or in part - be passed on, reproduced, published or otherwise used without prior written permission.

Proprietary confidential information © 2015 Boehringer Ingelheim International GmbH or one or more of its affiliated companies

Medium to high potency topical corticosteroids combined with oral antihistamines should be prescribed as early as possible. Systemic corticosteroids may be applied if deemed clinically necessary. Subjects should have a detailed photo-documentation of the skin and referred to a dermatologist immediately but no later than 24 hours for diagnosis, confirmation of severity, treatment and skin biopsy. These patients must be closely monitored.

The decision to stop or continue the study treatment should be based on the clinical judgement of the investigator taking into account the recommendation by the dermatologist.

Potentially Life-Threatening

Patients with potentially life-threatening skin reactions such as DRESS or SJS/TEN should be referred immediately for emergency care. Subjects should stop all study medications. Patients should be seen by a dermatologist for diagnosis, confirmation of severity, treatment and skin biopsy. The patient should be followed up as appropriate.

10.2.4 Photosensitivity reactions

Photosensitivity reactions typically resemble exaggerated sunburn with erythema and edema often occurring within minutes to hours of sun exposure, and may involve vesiculation and desquamation in some cases. These photosensitivity reactions affect exclusively the sun-exposed skin areas; therefore, a clear delimitation of the erythema by garments is typically observed.

10.2.4.1 Definition of severity of photosensitivity reactions

Mild: Any localized superficial 1st degree burn: erythema and edema, peeling.

Moderate: Extensive 1st degree sunburn (erythema, edema, peeling), or localized 2nd degree sunburn with blistering, serous exudates.

Severe: Extensive or full face 2nd degree sunburn with blistering, serous exudates.

10.2.4.2 Management guidelines for photosensitivity reactions

Symptomatic management of photosensitivity reaction of any severity includes cool compresses, soothing lotions, topical corticosteroids and/or systemic non-steroidal anti-inflammatory drugs (NSAID) or antipruritics. The need for UV light protection should be reinforced (Section 10.2.2).

Mild:

Treatment with study medication may be continued without interruption.

Moderate:

The patient should have a detailed photo-documentation of the skin and should be seen by a dermatologist as soon as possible but no later than 72 hours for diagnosis, confirmation of

Proprietary confidential information.
© 2014 Boehringer Ingelheim International GmbH or one or more of its affiliated companies. All rights reserved.
This document may not - in full or in part - be passed on, reproduced, published or otherwise used without prior written permission.

Proprietary confidential information © 2015 Boehringer Ingelheim International GmbH or one or more of its affiliated companies

severity, and treatment. Confirmed moderate photosensitivity reactions should be closely and frequently monitored at the investigator's discretion taking into account recommendation by dermatologist. These reactions do not require study treatment discontinuation.

Severe:

The subject should have a detailed photo-documentation of the skin and referred to a dermatologist immediately and within 24-48 hours for diagnosis, confirmation of severity, treatment and skin biopsy. These patients must be closely monitored. The decision to stop or continue the study treatment should be based on the clinical judgement of the investigator taking into account the recommendation by the dermatologist.

Proprietary confidential information.
© 2014 Boehringer Ingelheim International GmbH or one or more of its affiliated companies. All rights reserved.
This document may not - in full or in part - be passed on, reproduced, published or otherwise used without prior written permission.

Proprietary confidential information © 2015 Boehringer Ingelheim International GmbH or one or more of its affiliated companies

10.3 CHILD-TURCOTTE-PUGH CLASSIFICATION

Child-Turcotte-Pugh class and score (R99-1243 and R10-2440) employ five clinical measures of liver disease in the setting of liver cirrhosis: bilirubin, albumin, blood coagulation (as determined by prothrombin time [PT] or by INR), evidence of ascites, and evidence of encephalopathy.

To calculate the score, the limits presented in the table below are used.

Measure	1 Point	2 Points	3 Points
Bilirubin (mg/dL)*	<2*	2-3*	>3*
Albumin (g/dL)	>3.5	2.8-3.5	<2.8
Blood coagulation<br>PT: seconds prolonged<br>INR	 1-3<br><1.7	 4-6<br>1.7-2.3	 >6<br>>2.3
Ascites	None	Slight, or controlled medically	Moderate or severe
Encephalopathy	None	Grade 1-2	Grade 3-4

*For assessment of liver disease progress at Visit 6, Visit 8, Visit 10 and EOT, an increase in points due to increase in bilirubin value should only be added to the Child-Turcotte-Pugh score if the patient has a ratio of direct bilirubin/total bilirubin > 0.5.

For Child-Turcotte-Pugh class determination the scores are added and classified as:

- A: 5-6 points
- B: 7-9 points
- C: 10-15 points

Proprietary confidential information.
© 2014 Boehringer Ingelheim International GmbH or one or more of its affiliated companies. All rights reserved.
This document may not - in full or in part - be passed on, reproduced, published or otherwise used without prior written permission.

Proprietary confidential information © 2015 Boehringer Ingelheim International GmbH or one or more of its affiliated companies

11. DESCRIPTION OF GLOBAL AMENDMENTS

**Number of global amendment**		3
**Date of CTP revision**		11 Jun 2014
**EudraCT number**		2012-003534-17
**BI Trial number**		1241.30
**BI Investigational Product(s)**		BI 207127 (deleobuvir) in combination with faldaprevir
**Title of protocol**		A phase IIb open label study of BI 207127 in combination with faldaprevir and ribavirin in patients with moderate hepatic impairment (Child-Pugh B) with genotype 1b chronic hepatitis C infection
**To be implemented only after approval of the IRB / IEC / Competent Authorities**		☒
**To be implemented immediately in order to eliminate hazard – IRB / IEC / Competent Authority to be notified of change with request for approval**		☐
**Can be implemented without IRB / IEC / Competent Authority approval as changes involve logistical or administrative aspects only**		☐
**Section to be changed**		Title Page, Synopsis, Flow Chart, 2.2, 2.3, 3.1, 3.1.1.3, 3.2, 4.1, 4.2, 5.2.2, 6.2.2, 7.1, 7.2, 7.3, 7.5, 7.6, 10.1.2: 1, 10.1.2: 2
**Description of change**		Sections revised to remove Arm 3 and Cohort B from the trial design as well as all supporting text intended only for these treatment groups.
**Rationale for change**		Trial design modified due to sponsor decision to halt the development of deleobuvir (1241 program).
**Section to be changed**		Synopsis, Flow Chart, 3.1, 3.2, 5.5.1, 5.5.2, 6.2.3, 7.3, 10.1.1, 10.1.2: 1
**Description of change**		Week 16 intensive PK sampling and analysis removed. PK parameters edited. Protein binding sampling and analysis removed. Follow up period shortened from 96 to 12 weeks. Analyses

Proprietary confidential information.
© 2014 Boehringer Ingelheim International GmbH or one or more of its affiliated companies. All rights reserved.
This document may not - in full or in part - be passed on, reproduced, published or otherwise used without prior written permission.

Proprietary confidential information © 2015 Boehringer Ingelheim International GmbH or one or more of its affiliated companies

**Number of global amendment**		3
		for all endpoints to be done after last patient last visit.
**Rationale for change**		With the closing of the deleobuvir and faldaprevir programs, the sponsor will continue full courses of treatment for ongoing patients in Trial 1241.30, including through the post-treatment evaluation period. The standard for the post-treatment evaluation has become 12 weeks after completion of treatment (SVR12). In order to minimize the burden for the patients' remaining trial participations, while still providing current standard of care assessments for post-treatment relapse, the trial is being modified to make the SVR12 follow-up as the last trial follow-up visit. Additionally, for these reasons, exploratory observations and analyses have been removed.
**Section to be changed**		3.3.4
**Description of change**		Revised wording on reporting pregnancy for a female partner of a male patient.
**Rationale for change**		Clarification to ensure compliance with sponsor SOP.
**Section to be changed**		5.2.2.2
**Description of change**		Adminstrative edit.
**Rationale for change**		Removed duplicate paragraph in text.

**Number of global amendment**		2
**Date of CTP revision**		13 November 2013
**EudraCT number**		2012-003534-17
**BI Trial number**		1241.30
**BI Investigational Product(s)**		BI 207127 (deleobuvir) in combination with faldaprevir
**Title of protocol**		A phase III randomised, double-blind and placebo-controlled study of BI 207127 in combination with faldaprevir and ribavirin in patients with moderate hepatic impairment (Child-Pugh B) with genotype 1b chronic hepatitis C infection
**To be implemented only after approval of the IRB / IEC / Competent Authorities**		☒
**To be implemented**		☐

Proprietary confidential information.
© 2014 Boehringer Ingelheim International GmbH or one or more of its affiliated companies. All rights reserved.
This document may not - in full or in part - be passed on, reproduced, published or otherwise used without prior written permission.

Proprietary confidential information © 2015 Boehringer Ingelheim International GmbH or one or more of its affiliated companies

**Number of global amendment**		2
**immediately in order to eliminate hazard – IRB / IEC / Competent Authority to be notified of change with request for approval**		
**Can be implemented without IRB / IEC / Competent Authority approval as changes involve logistical or administrative aspects only**		☐
**Section to be changed**		2.3
**Description of change**		Potential risk of agranulocytosis/neutropenia is added.
**Rationale for change**		Cases of Grade IV neutropenia were recently observed and were added to benefit-risk assessment.
**Section to be changed**		2.3
**Description of change**		Deleted text that referenced dose reduction instructions.
**Rationale for change**		There are no instructions in the current dermatology management plan to reduce dose or interrupt treatment of BI 207127 or faldaprevir and these actions are also prohibited by section 4.1.4 of the protocol.
**Section to be changed**		3.3.2
**Description of change**		Corrected typo.
**Rationale for change**		The word "No" was mistakenly removed from an inclusion criterion when modified by protocol amendment 1.
**Section to be changed**		3.3.4.1
**Description of change**		To add treatment discontinuation if absolute neutrophil count is ≤ 500 cells/mm^3.
**Rationale for change**		To ensure treatment discontinuation in case of life-threatening neutropenia.

Proprietary confidential information.
© 2014 Boehringer Ingelheim International GmbH or one or more of its affiliated companies. All rights reserved.
This document may not - in full or in part - be passed on, reproduced, published or otherwise used without prior written permission.

Proprietary confidential information © 2015 Boehringer Ingelheim International GmbH or one or more of its affiliated companies

**Number of global amendment**		2
**Section to be changed**		Flow Chart, 4.2.1, 5.2.2.2, 6.2.3
**Description of change**		To record all SAEs with onset date after 28 days post-EOT until EOO, to define residual effect period, and to clarify SAE reporting requirements after trial completion..
**Rationale for change**		Update required as per sponsor's SOP.
**Section to be changed**		4.2.1
**Description of change**		Updated text on the management of gastrointestinal events.
**Rationale for change**		To clarify and provide additional details.
**Section to be changed**		5.2.3
**Description of change**		Inserted the word "AND".
**Rationale for change**		To further clarify central laboratory's flagging criteria for potential DILI cases.

**Number of global amendment**		1
**Date of CTP revision**		27 August 2013
**EudraCT number**		2012-003534-17
**BI Trial number**		1241.30
**BI Investigational Product(s)**		BI 207127 (deleobuvir) in combination with faldaprevir
**Title of protocol**		A phase III randomised, double-blind and placebo-controlled study of BI 207127 in combination with faldaprevir and ribavirin in patients with moderate hepatic impairment (Child-Pugh B) with genotype 1b chronic hepatitis C infection
**To be implemented only after approval of the IRB / IEC / Competent Authorities**		☒
**To be implemented immediately in order to eliminate hazard – IRB / IEC / Competent Authority to be notified of change with request for approval**		☐
**Can be implemented without**		☐

Proprietary confidential information.
© 2014 Boehringer Ingelheim International GmbH or one or more of its affiliated companies. All rights reserved.
This document may not - in full or in part - be passed on, reproduced, published or otherwise used without prior written permission.

Proprietary confidential information © 2015 Boehringer Ingelheim International GmbH or one or more of its affiliated companies

**Number of global amendment**		1
**IRB / IEC / Competent Authority approval as changes involve logistical or administrative aspects only**		
**Section to be changed**		Title Page
**Description of change**		Deleobuvir name is added for BI 207127.
**Rationale for change**		New name is added for completeness.
**Section to be changed**		Title Page and Synopsis
**Description of change**		Phase of trial changed from Ib/IIb to IIb/III.
**Rationale for change**		Updated to reflect intended classification by BI.
**Section to be changed**		Flow Chart, 2.3, 4.2.1, 5.2.4
**Description of change**		Increase frequency of ECG measurements. Add preliminary results from 1241.25 trial. Remove ondansetron as a recommended treatment of vomiting because of its QTc prolongation potential per updated List of Restricted and Use with Caution Concomitant Drugs, Version 3.0 in ISF. Add clarification that additional ECGs may be collected by the investigator for safety reasons, included but not limited to follow up of patients who experience palpitations, fainting, sudden loss of consciousness or syncope. Add that ECGs may be sent to the ECG laboratory to undergo central assessment.
**Rationale for change**		Based on the trial 1241.25 preliminary results suggesting a QTc increase and regardless of its methodological shortcomings, an increased ECG monitoring is warranted in the studies using FDV and BI 207127 combination until confirmatory data from the thorough QT study are available.
**Section to be changed**		Flow Chart, 3.3.2, Table 4.2.1: 2, Table 5.2.3:1, 5.2.3.1
**Description of change**		Add clarification that serum pregnancy tests will be performed for females of childbearing potential only at all marked visits on Flow Chart. Add clarification that urine (dipstick) pregnancy tests will be performed at Visit 2 for all females as part of eligibility assessment.
**Rationale for change**		This is not a change in the process, but a clarification.

Proprietary confidential information.
© 2014 Boehringer Ingelheim International GmbH or one or more of its affiliated companies. All rights reserved.
This document may not - in full or in part - be passed on, reproduced, published or otherwise used without prior written permission.

Proprietary confidential information © 2015 Boehringer Ingelheim International GmbH or one or more of its affiliated companies

**Number of global amendment**		1
**Section to be changed**		Abbreviations, 2.3, 3.3.4.1, Table 5.2.3:1, 10.2.3
**Description of change**		Add that DRESS has been reported in FDV program (in combination with PegIFN/RBV) at 120 and 240 mg QD doses. Refer to Section 10.2.3 for treatment discontinuation in case of potentially life-threatening skin reactions. Add DRESS to potentially life-threatening reaction examples. Add that patients need to be monitored for the appearance of systemic symptoms such as persistent fever, lymphadenopathy and/or involvement of mucous membranes and internal organs.
**Rationale for change**		Addition of new safety data from FDV project (PegIFN/RBV plus FDV) related to the occurrence of DRESS cases.
**Section to be changed**		3.3.2, 3.3.3
**Description of change**		Clarify definitions of treatment experienced patients eligible to enter the trial. Clarify exclusion of patients that have taken DAA. Clarify that Child Pugh C patients should be excluded.
**Rationale for change**		FDA and Investigators have requested clarification on these items.
**Section to be changed**		3.3.2, 3.3.3
**Description of change**		Modification of entry criteria to be more adapted to the Child Pugh B patient population.
**Rationale for change**		Child Pugh A patient recruitment is completed and several entry criteria were not suitable to recruit Child Pugh B patients.
**Section to be changed**		3.3.4.1
**Description of change**		Clarify treatment discontinuation if a female patient becomes pregnant and add guidance if a female partner of a male patient becomes pregnant.
**Rationale for change**		This is not a change in the process, but a clarification to ensure consistent management of potential pregnancy cases.
**Section to be changed**		3.3.4.1
**Description of change**		Explicitly add second confirmation in case of virologic breakthrough. Clarify the virologic breakthrough confirmation process.

Proprietary confidential information.
© 2014 Boehringer Ingelheim International GmbH or one or more of its affiliated companies. All rights reserved.
This document may not - in full or in part - be passed on, reproduced, published or otherwise used without prior written permission.

Proprietary confidential information © 2015 Boehringer Ingelheim International GmbH or one or more of its affiliated companies

**Number of global amendment**		1
**Rationale for change**		To streamline the assessment of virologic failure and clarify the criteria.
**Section to be changed**		Table 4.2.1: 2
**Description of change**		Treatment option for Arm 4 patients Flow Chart updated to include visit schedule listed in days and add liver disease progression assessment.
**Rationale for change**		To clarify time in days and to ensure a regular assessment of liver progression is done also for the treatment option patients.
**Section to be changed**		5.2.2.2
**Description of change**		Reporting of SAE, protocol-specified significant AE and relevant AE reporting within 24 hours is clarified.
**Rationale for change**		SAE reporting requirements were revised as per updated BI SOP.
**Section to be changed**		5.1.1, 5.2.3, Table 5.2.3:1, 5.5.2.2, 10.3
**Description of change**		Correct minor inconsistencies, typos, omissions and add minor clarifications.
**Rationale for change**		Minor administrative changes.

Proprietary confidential information.
© 2014 Boehringer Ingelheim International GmbH or one or more of its affiliated companies. All rights reserved.
This document may not - in full or in part - be passed on, reproduced, published or otherwise used without prior written permission.

Proprietary confidential information © 2015 Boehringer Ingelheim International GmbH or one or more of its affiliated companies

APPROVAL / SIGNATURE PAGE

Document Number: c02347351 **Version Number**: 1.0

Document Name: 1241-0030--protocol--revision 3

Title: A phase III randomised, double-blind and placebo-controlled study of BI 207127 in combination with faldaprevir and ribavirin in patients with moderate hepatic impairment (Child-Pugh B) with genotype 1b chronic hepatitis C infection

Signatures (obtained electronically)

Meaning of Signature	Signed by	Date Signed
Author-Trial Clinical Monitor	Kaste,Dr.,Renee	11 Jun 2014 20:03 CEST
Author-Statistician	Vinisko,Richard	12 Jun 2014 01:04 CEST
Author-Clinical Pharmacokineticist	Wu,Jing	12 Jun 2014 16:41 CEST
Approval-Team Member Medicine	Stern,Dr.,Jerry	14 Jun 2014 05:26 CEST
Approval-Therapeutic Area Head	Stern,Dr.,Jerry	14 Jun 2014 05:28 CEST
Verification-Paper Signature Completion	Kaste,Dr.,Renee	14 Jun 2014 17:40 CEST

Proprietary confidential information © 2015 Boehringer Ingelheim International GmbH or one or more of its affiliated companies

(Continued) Signatures (obtained electronically)

Meaning of Signature	Signed by	Date Signed

Proprietary confidential information © 2015 Boehringer Ingelheim International GmbH or one or more of its affiliated companies

CO-ORDINATING INVESTIGATOR SIGNATURE

Trial Title: **A phase** IIb open label **study of BI 207127 in combination with faldaprevir and ribavirin in patients with moderate hepatic impairment (Child-Pugh B) with genotype 1b chronic hepatitis C infection**

Trial Number: **1241.30**

Protocol Version: **4**

I herewith certify that I agree to adhere to the trial protocol and to all documents referenced in the trial protocol.

Date: 12 June 2014

Name: Michael P. Manns, Ph.D.,M.D. Signature: ____________

Affiliation:

Zentrum Innere Medizin
Medizinische Hochschule Hannover
Carl-Neuberg-Straße 1
30625 Hannover

Signed signature page is located in the electronic Clinical Trial Master File

Proprietary confidential information © 2015 Boehringer Ingelheim International GmbH or one or more of its affiliated companies

CO-ORDINATING INVESTIGATOR SIGNATURE

Trial Title: **A phase II randomised, double-blind and placebo-controlled study of BI 207127 in combination with faldaprevir and ribavirin in patients with moderate hepatic impairment (Child-Pugh B) with genotype 1b chronic hepatitis C infection**

Trial Number: **1241.30**

Protocol Version: **3**

I herewith certify that I agree to adhere to the trial protocol and to all documents referenced in the trial protocol.

Date: 22.11.13

Name: Michael P. Manns, Ph.D.,M.D. Signature: [signature]

Affiliation:

Zentrum Innere Medizin
Medizinische Hochschule Hannover
Carl-Neuberg-Straße 1
30625 Hannover

Signed signature page is located in the electronic Clinical Trial Master File

Proprietary confidential information © 2015 Boehringer Ingelheim International GmbH or one or more of its affiliated companies

CO-ORDINATING INVESTIGATOR SIGNATURE

Trial Title: **A phase III randomised, double-blind and placebo-controlled study of BI 207127 in combination with faldaprevir and ribavirin in patients with moderate hepatic impairment (Child-Pugh B) with genotype 1b chronic hepatitis C infection**

Trial Number: **1241.30**

Protocol Version: **2**

I herewith certify that I agree to adhere to the trial protocol and to all documents referenced in the trial protocol.

Date: 28-08-2013

Name: Michael P. Manns, Ph.D.,M.D. Signature: [signature]

Affiliation:

Zentrum Innere Medizin
Medizinische Hochschule Hannover
Carl-Neuberg-Straße 1
30625 Hannover

Signed signature page is located in the electronic Clinical Trial Master File

Clinical Research Germany
03. Sep. 2013
Gruppe Resp./Immun./Virol.
